# A Dietary Supplement Containing Cinnamon, Chromium and Carnosine Decreases Fasting Plasma Glucose and Increases Lean Mass in Overweight or Obese Pre-Diabetic Subjects: A Randomized, Placebo-Controlled Trial

**DOI:** 10.1371/journal.pone.0138646

**Published:** 2015-09-25

**Authors:** Yuejun Liu, Aurélie Cotillard, Camille Vatier, Jean-Philippe Bastard, Soraya Fellahi, Marie Stévant, Omran Allatif, Clotilde Langlois, Séverine Bieuvelet, Amandine Brochot, Angèle Guilbot, Karine Clément, Salwa W. Rizkalla

**Affiliations:** 1 Institute of Cardiometabolism and Nutrition, ICAN, Assistance Publique—Hôpitaux de Paris, Heart and Nutrition Department, Pitié-Salpêtrière Hospital, and Human Nutrition Research Center—Ile de France, 75013, Paris, France; 2 INSERM, UMR S U1166, Nutriomics, 75013, Paris, France; 3 Sorbonne University, Pierre and Marie Curie University, Paris 06, UMR_S 1166 I, Nutriomics Team, Paris, France; 4 Assistance Publique-Hôpitaux de Paris, Biochemistry and Hormonology Department, Tenon Hospital, 75970, Paris, France; 5 AdipoPhYt, 75013, Paris, France; 6 Groupe PiLeJe, 75015, Paris, France; Indiana University Richard M. Fairbanks School of Public Health, UNITED STATES

## Abstract

**Background:**

Preventing or slowing the progression of prediabetes to diabetes is a major therapeutic issue.

**Objectives:**

Our aim was to evaluate the effects of 4-month treatment with a dietary supplement containing cinnamon, chromium and carnosine in moderately obese or overweight pre-diabetic subjects, the primary outcome being change in fasting plasma glucose (FPG) level. Other parameters of plasma glucose homeostasis, lipid profile, adiposity and inflammatory markers were also assessed.

**Methods:**

In a randomized, double-blind, placebo-controlled study, 62 subjects with a FPG level ranging from 5.55 to 7 mmol/L and a body mass index ≥25 kg/m^2^, unwilling to change their dietary and physical activity habits, were allocated to receive a 4-month treatment with either 1.2 g/day of the dietary supplement or placebo. Patients were followed up until 6 months post-randomization.

**Results:**

Four-month treatment with the dietary supplement decreased FPG compared to placebo (-0.24±0.50 *vs* +0.12±0.59 mmol/L, respectively, p = 0.02), without detectable significant changes in HbA1c. Insulin sensitivity markers, plasma insulin, plasma lipids and inflammatory markers did not differ between the treatment groups. Although there were no significant differences in changes in body weight and energy or macronutrient intakes between the two groups, fat-free mass (%) increased with the dietary supplement compared to placebo (p = 0.02). Subjects with a higher FPG level and a milder inflammatory state at baseline benefited most from the dietary supplement.

**Conclusions:**

Four-month treatment with a dietary supplement containing cinnamon, chromium and carnosine decreased FPG and increased fat-free mass in overweight or obese pre-diabetic subjects. These beneficial effects might open up new avenues in the prevention of diabetes.

**Trial Registration:**

ClinicalTrials.gov NCT01530685

## Introduction

Type 2 diabetes (T2D) constitutes a growing global epidemic worldwide and is associated with numerous disabling and life-threatening complications, as well as a major economic burden [1]. Individuals with impaired fasting glucose homeostasis and/or impaired glucose tolerance (referred to as pre-diabetic) are at high risk for the future development of T2D, especially if they are also overweight [1–4]. Moreover, micro- and macrovascular damage is already present in pre-diabetic individuals [5–9]. Preventing or delaying progression to T2D in this population is therefore a major public health issue and therapeutic goal [8–10]. Recommendations include lifestyle interventions (i.e. switch to a healthier diet and increased physical activity), and use of various medications [3–10]. However, lifestyle interventions are difficult to maintain in the long term and medications may be associated with side effects [7–9].

Nutraceuticals may represent a valuable alternative or adjunct to conventional prescription drugs in such a context. Among these, cinnamon has been shown to possess anti-diabetic and anti-inflammatory properties in experimental studies [11–14]. However, despite promising initial findings in T2D patients [15], subsequent clinical studies have generated conflicting results, probably owing to differences in the population included, dose range, dosage form, treatment duration, confounding concomitant treatments and quality of study design [16–20]. To the best of our knowledge, only one study has evaluated the effect of cinnamon in pre-diabetic subjects, showing that this was effective in lowering fasting plasma glucose (FPG) and oxidative stress [21], and might therefore be able to prevent or delay progression to T2D [21;22]. Chromium, an essential mineral, has been proposed to act as a regulator of glycemic homeostasis with an effect on HbA1c [23–25]. Another micronutrient candidate that could also be considered in this context is carnosine, a naturally occurring compound with antiglycation and anti-aging activities [26], which might potentiate any favorable effect of cinnamon on glycemic control in pre-diabetic subjects. Finally, both cinnamon and chromium have been suggested to have favorable effects on body composition [21;27].

We therefore conducted a randomized, double-blind, placebo-controlled study to evaluate the effects of a 4-month treatment with a micronutrient dietary supplement containing cinnamon, chromium and carnosine in overweight or obese pre-diabetic subjects, with change in FPG level as the primary outcome. Glycemic control, insulin sensitivity, adiposity, lipid profile and inflammatory markers were also explored.

## Subjects and Methods

### Subjects

Subjects were enrolled in the study between November 2011 and April 2012. The last subject completed the study on August 2012. Subjects aged between 25 and 65 years, overweight (body mass index ≥25 kg/m^2^), presenting a FPG level between 5.55 mmol/L and 7 mmol/L [2] and unwilling to change their usual dietary and physical activity habits were eligible for randomization.

Subjects with overt diabetes, any abnormality in renal, hepatic or thyroid function, hypogonadism, history of musculoskeletal, autoimmune or neurologic disease, or human immunodeficiency or hepatitis C virus infection, or having experienced weight loss of more than 5% within the previous six months, were excluded. Other exclusion criteria were consumption of any supplement containing cinnamon or chromium, or any current treatment potentially interfering with plasma glucose homeostasis or body weight control. Finally, women of childbearing age were excluded if they were pregnant, breast-feeding, or not using a reliable contraceptive method.

### Study design

This was a single center, randomized, double-blind, placebo-controlled, two-parallel group study conducted at the outpatient clinic of the Nutrition Department at the Pitié-Salpêtrière Hospital, Institute of Cardiometabolism and Nutrition (ICAN), Paris, France.

Participants were randomized on the basis of a computer-generated list provided by PiLeJe Institute (Saint-Laurent-des-Autels, France) to receive either a micronutrient dietary supplement or placebo for 4 months. Face-to-face follow-up visits were scheduled at 2, 4 (end of treatment period) and 6 (end of study) months from randomization. Participants were instructed to maintain their usual lifestyle during the experimental period.

The study was conducted according to the ethical principles of the Declaration of Helsinki and local regulations. The protocol was approved by the independent ethics committee of Pitié-Salpêtrière Hospital on December 1^st^, 2010, and obtained the authorization of the French agency for the security of medications and health products–Afssaps changed to ANSM on 2 November 2010. A written informed consent was obtained from all patients before inclusion. This clinical trial was registered before enrollment of participants in the EU Clinical trials Register under the identification number: ID RCB 2010-A00776-33. It was also registered at ClinicalTrials.gov under the identification number: NCT01530685 (URL: clinicaltrials.gov/show/NCT01530685?displayxml=true) later due to some technical problems with the sponsor of the study. The authors confirm that all ongoing and related trials for this drug/intervention are registered.

### Treatments

Participants received orally, during lunch, two capsules per day of either a dietary supplement, containing: cinnamon, chromium and carnosine (Glycabiane^®^, PiLeJe, Saint-Laurent-des-Autels, France) or placebo (Table 1). The capsules of the placebo and the dietary supplement were identical in colour, form and smell. There was a 100 mg difference in weight between the two capsules, which could not be detected by handling. Therefore, the double-blinded treatment was correctly respected for the subjects and the medical staff. The cinnamon used (ChalCinn^®^, PiLeJe, Saint-Laurent-des-Autels, France) was an extract of cinnamon bark (*Cinnamomum cassia*), rich in polyphenol type-A polymers (oligomeric proanthocyanidins-A: OPC-A). Chromium was used in the form of chromium chloride (Guanylor^®^, PiLeJe, Saint-Laurent-des-Autels, France).

**Table 1:** Composition of the dietary supplement and placebo capsules. HPMC: hydroxy-propyl-methylcellulose.

Ingredient	Dietary supplement (Quantity per capsule)	Placebo (Quantity per capsule)
**Extract of cinnamon**	228.00 mg	-
**L-carnosine**	100.00 mg	-
**Chromium guanylate**	1.25 mg (10 μg chromium chloride)	-
**Excipients**		
Silica	16.00 mg	-
Talc	7.00 mg	7.00 mg
Magnesium stearate	6.00 mg	6.00 mg
Hydrated silica	5.00 mg	5.00 mg
Silicon dioxide	-	16.00 mg
Microcristalline cellulose	-	230.25 mg
**Clear transparent HPMC capsule**	95.00 mg	95.00 mg
**Total**	**595 mg**	**496 mg**

Treatment compliance was assessed by means of a diary in which all capsules taken were to be recorded by the participants, and by counting the number of capsules returned by the participants at the end of the treatment period. Any treatment likely to interfere with glycemic control was prohibited throughout the study.

### Outcome measures

The primary efficacy outcome was the change in FPG level at 4 months. Secondary outcomes were considered as exploratory and included changes at 4 months in other parameters of glucose homeostasis (fasting plasma insulin, plasma glycated hemoglobin [HbA1c], and markers of insulin resistance/sensitivity), parameters of lipid homeostasis (plasma triglycerides, total cholesterol, high-density [HDL] and low-density [LDL] lipoprotein cholesterol, free fatty acids), adiposity markers (body weight, body mass index, fat mass and fat-free mass, adipocyte diameter), adipokines (plasma leptin, adiponectin), and markers of inflammation (high-sensitivity C-reactive protein [hs-CRP], interleukin-6 [IL-6]) and cardiovascular risk (plasminogen activator inhibitor type 1 [PAI-1]). Metabolic activity of adipose tissue *in vitro* (secretion of adiponectin and IL-6, insulin sensitivity estimated by the measurement of serine/threonine protein kinase B [PKB, also known as Akt]) was also explored, and dietary intakes and lifestyle were assessed. The safety outcome was the incidence of adverse events, whether serious or not, during the study period.

Participants underwent a series of explorations after a 12-hour overnight fast.

Adipose tissue samples and body composition were analyzed at baseline (Day 0) and at the end of the treatment period (Month 4). A sample of subcutaneous abdominal adipose tissue (SCAT) in the periumbilical area was obtained from each patient on arrival, by needle aspiration (using a 14-gauge needle) under local anesthesia with 10% lidocaine hydrochloride solution not containing epinephrine (Xylocaine^®^, AstraZeneca, Rueil Malmaison, France). Fresh aliquots of this sample were used to measure adipocyte diameter and to explore *in vitro* adipose tissue metabolism. For adipocyte diameter measurement, a portion of each SCAT biopsy sample was immediately isolated by collagenase digestion and cell size measurements were performed as previously described [28]. Another portion was cultured for 24 hours, the secretion medium then being collected and frozen at -80°C for determination of IL-6 and adiponectin levels [29]. The adipose tissue samples were also used to evaluate insulin sensitivity by measuring insulin stimulation of Akt [30].

Body composition (fat and fat-free mass distribution) was determined using dual-energy X-ray absorptiometry (Lunar Prodigy, General Electric Medical Systems, Madison, WI, USA).

At Day 0, Month 4 and Month 6 (end of the follow-up period), blood samples were withdrawn after an overnight fast. Plasma and serum were rapidly separated and frozen at -80°C for measurement of the variables of interest.

We used several surrogates to estimate pancreatic β cell function (insulin secretion) and insulin sensitivity using the Homeostasis Model Assessment—Continuous Infusion Glucose Model Assessment (HOMA-CIGMA) [31] and Disse indices [32]. The revised quantitative insulin sensitivity check index (QUICKI) was also calculated. Plasma glucose was measured by the hexokinase method (ARCHITECT^®^ system, Abbott Diagnostics, Abbott Park, IL, USA). Plasma insulin was determined by chemiluminescence (ARCHITECT^®^ system). Plasma triglycerides and free fatty acids were measured using Biomerieux kits (Biomerieux, Marcy l’Etoile, France), and total, HDL, and LDL cholesterol using Labintest kits (Labintest, Aix-en-Provence, France). Enzyme-linked immunosorbent assay (ELISA) kits were used to assay leptin, IL-6 (Quantikine^®^, R&D Systems, Oxford, UK) and adiponectin (Bühlman, Basle, Switzerland). High-sensitivity C-reactive protein (hs-CRP) was measured by immuno-nephelometry using IMMAGE Immunochemistry Systems (Beckman Coulter, Villepinte, France). Plasma levels of PAI-1 were determined using Chromolize/PAI-1 kits (Biopool International, CA, USA).

Dietary intakes were monitored by a registered dietician according to the information obtained from each subject’s 3-day dietary record. All records were collected using the computer software MXS program (Medical Expert System, www.mxs-sante.fr). Lifestyle was evaluated using the Three-Factor Eating Questionnaire [33] and Baecke physical activity questionnaire [34].

### Power calculation

The trial was designed to demonstrate superiority of the dietary supplement over placebo. Assuming a 7% change in FPG in the active treatment group based on internal unpublished data and a previous study [21], i.e. 0.39 mmol/L for changes in FPG between the dietary supplement and placebo groups during the treatment periods, with a standard deviation (SD) of 0.41 mmol/L (for changes between 4 months and baseline), a sample of 25 evaluable subjects per group was considered to have ≥90% power to detect a between-treatment difference in FPG change with a type I error rate of 5%. Assuming that 15 to 20% of subjects would be non-evaluable for the primary efficacy outcome, at least 60 subjects (30 per group) were to be included.

### Statistical analysis

The principal efficacy analysis was a per-protocol analysis performed on subjects who had respected the stipulated interval between visits (±10 days) and who had received at least 85% of the scheduled study treatment (compliers). A second efficacy analysis, performed for confirmatory purposes, and the safety analysis were performed on the intention-to-treat population (i.e. all randomized subjects).

Values are expressed as the mean±SD in the tables and as the mean±SEM (standard error of the mean) in the figures. Variables were tested for normality using the Shapiro-Wilk test, and subjected to logarithmic or Anscombe transformation, where appropriate, to conform to the normality assumptions (Table A in S1 File). A paired Student’s t-test was used for comparisons between the different time points in each group, an unpaired Student’s t-test being used to compare the treatment effect between the two groups. An ANCOVA adjusted for variations of fat-free mass (%) was used to check whether the observed difference in FPG level between the two groups was independent of fat-free mass.

Several exploratory *post-hoc* analyses were performed in the dietary supplement and placebo groups. In an attempt to understand the effects of the dietary supplement on FPG responses, we searched for factors potentially predictive of decreased FPG level. Correlations between changes in FPG level during the dietary supplement treatment and various clinical and biological variables at baseline were investigated using Pearson correlations.

Furthermore, in the dietary supplement group in order to classify subjects according to their ability to secrete insulin and the expected impact on FPG, the correlation between changes in FPG level and changes in insulin secretion (HOMA-B%) was tested. The baseline values of the identified two groups of subjects (separated by the correlation line) were compared using the nonparametric Mann Whitney test to identify the two different phenotypes. Correlations between changes in fat-free mass, changes in insulin sensitivity (revised QUICKI), and changes in free fatty acids were also explored.

Statistical analyses were performed using R software version 2.13 (http://www.r-project.org), with the following R packages: plotrix 3.4–5 [35] and nlme 3.1–105 [36]. Two-tailed *p-*values of less than 0.05 were considered statistically significant.

## Results

### Study population

**Fig 1:**
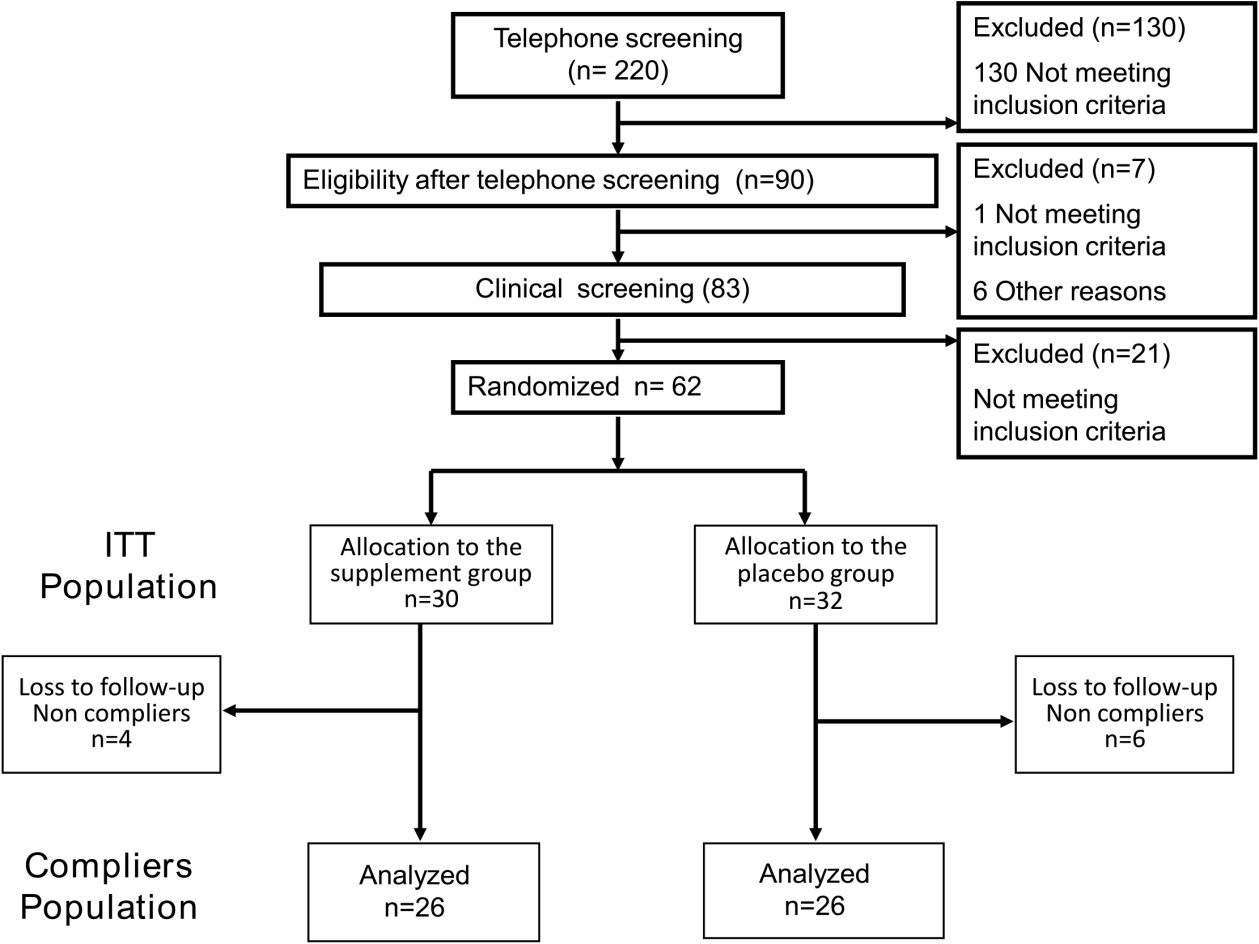
Consort flow diagram. ITT: intention-to-treat. One subject in the dietary supplement group was lost to follow-up at Month 2, and one subject in the placebo group was withdrawn from the study at Month 2 due to a serious adverse event not related to study treatment (new condition with a need for hormonal treatment).

Sixty-two subjects (40 women, 22 men) were selected from an initial panel of 220 screened subjects and randomized (intention-to-treat population) (Fig 1). Two subjects, one in each treatment group, lacked efficacy data at Month 4. Eight subjects showed non-compliance with the stipulated supplement dosage (less than 85% of the allocated treatment) or study treatment duration (10 days less or more than the allowed interval). Baseline characteristics did not differ significantly between subjects not considered in the per-protocol efficacy analysis (n = 10) and those included in this analysis (n = 52), although numerically the proportion of men was higher and mean age was lower in the former population (Table B in S1 File).

Baseline characteristics did not differ between the two treatment groups, in either the intention-to-treat or the per-protocol population (Table 2). According to self-reported data, the subjects’ lifestyle and physical activity remained unchanged throughout the study in both groups (Table C in S1 File).

**Table 2:** Baseline characteristics of subjects in the intention-to-treat and per-protocol populations. Values are mean±SD; ITT: intention-to-treat; PP: per-protocol; F: female; M: male. The baseline data in the dietary supplement and placebo groups were compared using Student’s t-tests for quantitative variables and Fisher's exact test for sex. No statistically significant difference was found between the two treatment groups in either of the two study populations.

	ITT population			PP population		
	Placebo	Glycabiane	Changes in Placebo vs. Glycabiane	Placebo (n = 32)	Glycabiane	Changes in Placebo vs. Glycabiane
	Baseline (day 0)	Baseline (day 0)	P value	Baseline (day 0)	Baseline (day 0)	P value
**Dietary control**						
Energy (kcal/d)	2037.7 ± 591.4	1883.3 ± 703.8	0.28	1989.5±448.5	1883.8±735.9	0.38
Carbohydrates (%)	41.9 ± 7.1	40.9 ± 8.3	0.61	42.8 ±6.7	39.6±7.8	0.13
Protein (%)	17.6 ± 3.5	17.6 ± 3.8	0.90	17.6±3.6	17.7±3.9	0.96
Lipid (%)	37.5 ± 5.1	37.4 ± 5.8	0.90	37.1±5.5	37.9±5.8	0.59
**Adiposity markers**						
Body weight (kg)	87.6 ± 14.4	86.1 ± 9.8	0.63	87.5±13.7	85.8±10.2	0.59
BMI (kg/m2)	31.6 ± 4.7	31.2 ± 3.1	0.82	31.6 ± 4.5	31.4 ± 3.1	0.95
Fat mass (kg)	33.7 ± 8.7	33.5 ± 6.8	0.92	33.8±9.0	34.04±7.1	0.92
Fat mass (%)	39.00 ± 6.4	39.8 ± 7.3	0.62	39.1±6.8	40.6±7.2	0.46
Fat-free mass (kg)	49.9 ± 9.1	48.4 ± 8.9	0.52	49.6±8.7	47.6±8.7	0.39
Fat-free mass (%)	58.1 ± 6.1	57.3 ± 7.0	0.60	58.0±6.5	56.6±6.9	0.45
Adipocyte diameter (μm)	111.1 ± 9.6	114.4 ± 7.2	0.14	111.3±10.5	114.0±7.3	0.30
**Lipid Homeostasis**						
Triacylglycerol (g/L)	1.3 ± 0.5	1.3 ± 0.7	0.76	1.3±0.5	1.3±0.7	0.47
Total cholesterol (g/L)	2.2± 0.4	2.1 ± 0.4	0.25	2.3±0.5	2.1±0.4	0,29
HDL cholesterol (g/L)	0.5 ± 0.1	0.5 ± 0.2	0,18	0.5±0.1	0.6±0.2	0,07
LDL cholesterol (g/L)	1.5 ± 0.4	1.3 ± 0.4	0,09	1.5±0.4	1.4±0.4	0,10
FFA (mmol/L)	0.4 ± 0.2	0.4 ± 0.2	0.85	0.4±0.2	0.5±0.2	0.18
**Glucose homeostasis**						
Fasting Plasma glucose (mmol/L)	6.0 ± 0.6	6.1 ± 0.6	0.72	6.1±0.6	6.1±0.6	0,75
Fasting Plasma insulin (μU/mL)	9.3 ± 5.4	9.9 ± 4.2	0.47	9.7±5.7	9.4±3.5	0,86
HbA1c (%)	5.9 ± 0.4	5.9 ± 0.4	0.86	5.9±0.4	5.9±0.4	0,57
HOMA-IR	1.3 ± 0.7	1.4 ± 0.6	0.46	1.3±0.8	1.3±0.5	0,84
HOMA-S (%)	101.4 ± 51.5	92.2 ± 57.9	0.47	99.7±54.2	95.1±59.3	0.84
HOMA-B (%)	73.9 ± 24.4	76.7 ± 27.2	0.68	75.0±25.4	72.3±17.6	0.83
Revised Quicki	0.4 ± 0.1	0.4 ± 0.04	0.48	0.4±0.1	0.4±0.04	0,29
Disse index	-7.6 ± 6.42	-6.8 ± 4.8	0.62	-7.7±6.8	-6.3±4.5	0,42
**Adipokines and Inflammatory factors**						
Leptin (ng/mL)	30.2 ± 22.9	31.0 ± 21.6	0.71	32.6±24.0	32.0±21.9	0.82
Adiponectin (μg/mL)	5.0 ± 3.4	4.5 ± 2.2	0.62	5.0±3.5	4.6±2.1	0.96
hs-CRP(mg/L)	5.3 ± 6.4	4.0 ± 4.3	0.49	5.9±6.8	4.0±4.3	0.35
PAI-1 (ng/mL)	25.8 ± 21.3	26.0 ± 20.4	0.75	26.5±22.6	25.8±20.0	0.74
IL-6 (pg/mL)	2.0 ± 1.3	1.9 ± 1.3	0.96	1.9±1.3	1.9±1.3	0.99
**Adipokines explored from adipose tissue**						
Adiponectin (pg/mL)	10415.4 ± 10185.4	8883.2 ± 6408.2	0,87	11877±10501	9283 ±6581	0,51
IL-6 (pg/mL)	536.0 ± 420.2	1099.2 ± 1699.3	0,15	576 ± 446	1130 ± 1777	0,25
Akt (arbitratry U)	13.8 ± 14.8	11.4 ± 13.8	0,49		15.2 ± 15.8	9.5 ± 11.5	0,23

### Energy intake and body composition

Changes in energy and in macronutrient intakes were similar in the two groups (Table 3). Body weight and body mass index increased significantly at Month 4 in both treatment groups with no statistically significant between-group difference. However, fat-free mass increased significantly in the dietary supplement group compared to the placebo group, in terms of both percentage (p = 0.02) and absolute (p = 0.008) values.

**Table 3:** Study variables before and after 4-month treatment with dietary supplement or placebo. Values are expressed as mean±SD. M4: Month 4; FPG: fasting plasma glucose. FP insulin: fasting plasma insulin; HbA1c: glycated hemoglobin; HOMA-IR: homeostatic model assessment-insulin resistance; HOMA-B (%): β cell function; HOMA-S (%): insulin sensitivity; QUICKI: quantitative insulin sensitivity check index; HDL: high-density lipoprotein; LDL: low-density lipoprotein; FFA: free fatty acids; hs-CRP: high-sensitivity C-reactive protein; PAI-1: plasminogen activator inhibitor-1; IL-6: interleukin-6; Akt: serine/threonine protein kinase B. Baseline data did not differ between groups using unpaired Student t-tests. Baseline and 4-month data in each group were compared using paired Student’s t-tests; Changes (values at 4 months–values at baseline/values at baseline*100) in the placebo and dietary supplement groups were compared using unpaired Student’s t-test.

	Placebo (n = 26)			Dietary supplement (n = 26)			Changes in placebo vs dietary supplement
	Baseline (Day 0)	After treatment (M4)	P value Day 0 *vs*. M4	Baseline (Day 0)	After treatment (M4)	P value Day 0 *vs*. M4	P value
**Dietary intake**							
Energy (kcal/day)	1989.5±448.5	2045.1±611.1	0.69	1883.8±735.9	1898.6±572.4	0.80	0.90
Carbohydrates (%)	42.8 ±6.7	42.74±7.8	0.96	39.6±7.8	39.3±7.5	0.82	0.91
Proteins (%)	17.6±3.6	17.6±3.8	0.91	17.7±3.9	17.9±3.5	0.69	0.70
Lipids (%)	37.1±5.5	35.7±5.8	0.30	37.9±5.8	37.6±4.5	0.76	0.55
**Glucose homeostasis**							
FPG (mmol/L) (mmol/L)mmol/L)	6.1±0.6	6.2±0.8	0.36	6.1±0.6	5.9±0.6	0.026	0.020
FP insulin (μU/mL)	9.7±5.7	9.0±4.5	0.77	9.4±3.5	9.9±3.9	0.25	0.33
HbA1c (%)	5.96±0.40	6.12±0.50	0.00015	5.89±0.43	5.99±0.47	0.015	0.32
HOMA-IR	1.3±0.8	1.3±0.6	0.80	1.29±0.5	1.3±0.5	0.31	0.39
HOMA-S (%)	99.7±54.2	99.1±46.6	0.83	95.1±59.3	85.7±34.1	0.31	0.41
HOMA-B (%)	75.0±25.4	68.4±18.4	0.5	72.3±17.6	81.7±19.8	0.043	0.06
Revised QUICKI	0.4±0.1	0.4±0.1	0.29	0.4±0.04	0.38±0.03	0.083	0.66
Disse index	-7.7±6.8	-7.4±6.3	0.76	-6.3±4.5	-7.8±4.5	0.040	0.88
**Lipid Homeostasis**							
Triacylglycerol (g/L)	1.3±0.5	1.3±0.7	0.52	1.3±0.7	1.3±0.7	0.56	0.91
Total cholesterol (g/L)	2.3±0.5	2.2±0.4	0.36	2.1±0.4	2.1±0.4	0.86	0.49
HDL cholesterol (g/L)	0.5±0.1	0.5±0.2	0.63	0.6±0.2	0.5±0.1	0.12	0.56
LDL cholesterol (g/L)	1.5±0.4	1.45±0.3	0.41	1.4±0.4	1.4±0.5	0.40	0.24
FFA (mmol/L)	0.4±0.2	0.4±0.2	0.11	0.5±0.2	0.5±0.2	0.24	0.96
**Adiposity markers**							
Body weight (kg)	87.5±13.7	88.6±14.1	0.035	85.8±10.2	86.8±10.2	0.020	0.81
Body mass index (kg/m^2^)	31.6 ± 4.5	31.9 ± 4.7	0.035	31.4 ± 3.1	31.8 ± 3.2	0.014	0.79
Fat mass (kg)	33.8±9.0	34.5±9.4	0.041	34.04±7.1	34.0±6.5	0.94	0.26
Fat mass (%)	39.1±6.8	39.7±7.4	0.09	40.6±7.2	40.0±6.5	0.15	0.026
Fat-free mass (kg)	49.6±8.7	49.5±9.0	0.58	47.6±8.7	48.8±8.2	0.003	0.008
Fat-free mass (%)	58.0±6.5	57.4±7.0	0.09	56.6±6.9	57.2±6.2	0.14	0.020
Adipocyte diameter (μm)	111.3±10.5	111.7±10.0	0.82	114.0±7.3	116.6±5.7	0.045	0.18
**Adipokines and markers of inflammation**							
Leptin (ng/mL)	32.6±24.0	32.6±23.7	0.99	32.0±21.9	32.9±20.9	0.35	0.54
Adiponectin (μg/mL)	5.0±3.5	4.6±2.2	0.63	4.6±2.1	4.7±2.4	0.97	0.83
hs-CRP (mg/L)	5.9±6.8	6.0±8.6	0.30	4.0±4.3	3.7±5.0	0.59	0.59
PAI-1 (ng/mL)	26.5±22.6	32.4±22.5	0.07	25.8±20.0	33.1±22.2	0.11	0.85
IL-6 (pg/mL)	1.9±1.3	1.9±1.6	0.48	1.9±1.3	1.9±1.1	0.92	0.66
**Adipokines assayed in adipose tissue**							
Adiponectin (pg/mL)	11877±10501	9656 ±8068	0.19	9283 ±6581	9768 ± 6628	0.72	0.38
IL-6 (pg/mL)	576 ± 446	929 ± 1279	0.22	1130 ± 1777	1487.35 ±2120	0.24	0.92
Akt (arbitrary U)	15.2 ± 15.8	16.8 ± 17.5	0.89	9.5 ± 11.5	16.0 ± 15.7	0.23	0.40

### Plasma glucose homeostasis and insulin sensitivity surrogates

At Month 4, a significant decrease in FPG level was seen in the dietary supplement group (absolute changes:-0.24±0.50 mmol/L) compared to baseline (p = 0.026), whereas no change being observed in the placebo group (absolute change: +0.12±0.59 mmol/L, p = 0.36) (Table 3). The difference between the groups was statistically significant (p = 0.02; Table 3 and Figure A in S1 File). At the end of follow-up (two months after treatment cessation), FPG tended to return towards baseline values in the dietary supplement group (Figure A in S1 File). However, three different kinetic trajectories for FPG were identified in this group (Figure B-A in S1 File **)**: 1) in six subjects, FPG decreased during the treatment period (-5%), with a further decline at the end of follow-up (-11%); 2) in 11 subjects, FPG decreased during treatment (-9.7%) and then returned to baseline; 3) in nine subjects, FPG slightly increased during treatment, subsequently remaining constant throughout follow-up. In the placebo group only two different kinetic trajectories were identified (Figure B-B in S1 File): 1) in 17 subjects, FPG declined slightly (-2.7%) during the treatment period and remained stable throughout follow-up; 2) in nine subjects, FPG showed an elevation of 11% during the treatment period then a reduction at the end of follow-up. Kinetic trajectories in the placebo group were totally different from those observed in the treatment group: while two clusters in the dietary supplement group (representing 65% of included subjects) showed a decrease of either -5 or -10 of FPG, only one cluster in the placebo group showed a slight decrease of -2.7% in 65% of subjects.

HbA1c levels were slightly higher at Month 4 than at baseline in both groups, with no between-group difference. Whereas fasting plasma insulin level did not change within either group, insulin secretion, evaluated by HOMA-B%, increased significantly in the dietary supplement group and numerically decreased in the placebo group, with a trend towards statistical significance for the between-group comparison (p = 0.06; Table 3). There was an improvement in insulin sensitivity in the dietary supplement group (a trend for revised QUICKI, p = 0.08 and a significant effect for Disse index, p = 0.04), but this improvement did not differ from that observed in the placebo group, whether evaluated by HOMA-IR, revised QUICKI or Disse Index.

**Fig 2:**
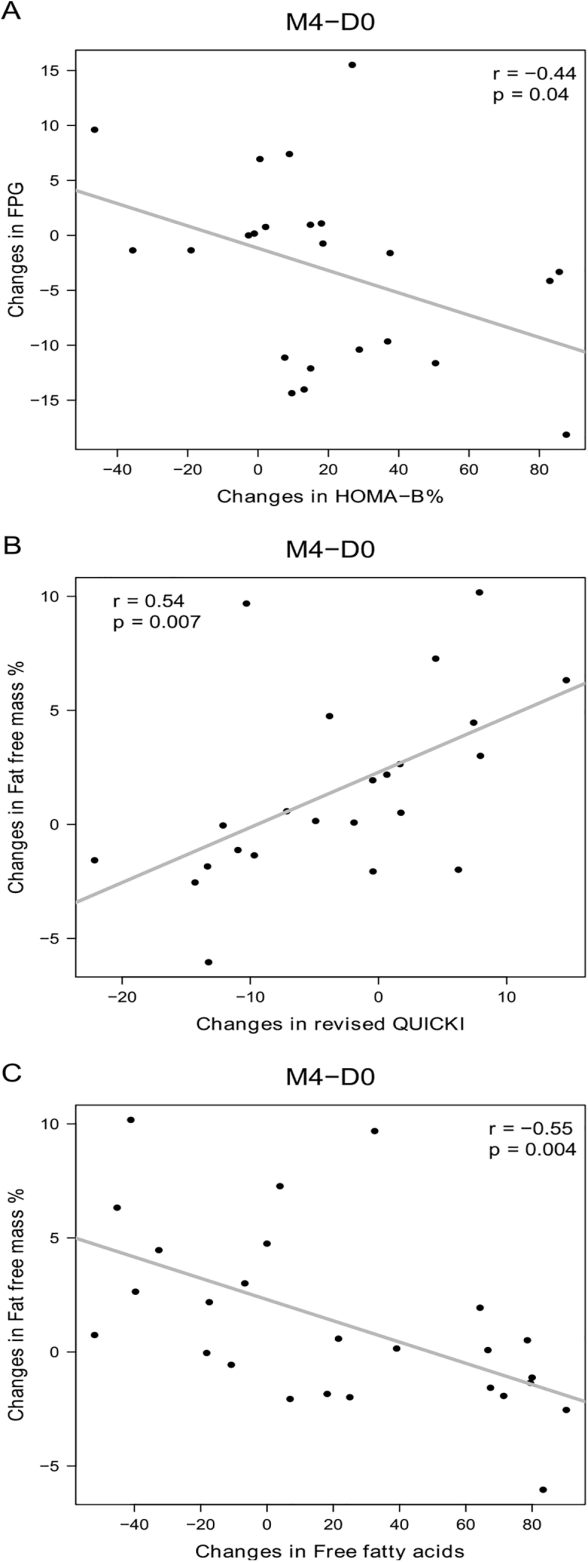
Correlations between changes in FPG or fat-free mass and other bioclinical parameters during dietary supplement treatment. A: Correlation between changes in fasting plasma glucose and insulin secretion estimated by HOMA-B%. B: Correlations between changes in fat-free mass and changes in insulin sensitivity (estimated by revised QUICKI). C: Correlations between changes in fat-free mass and free fatty acids; N = 23 in A and B due to 3 missing data for plasma insulin, N = 26 subjects in C. Pearson correlations were used. D0: Day 0; M4: Month 4.

**Fig 3:**
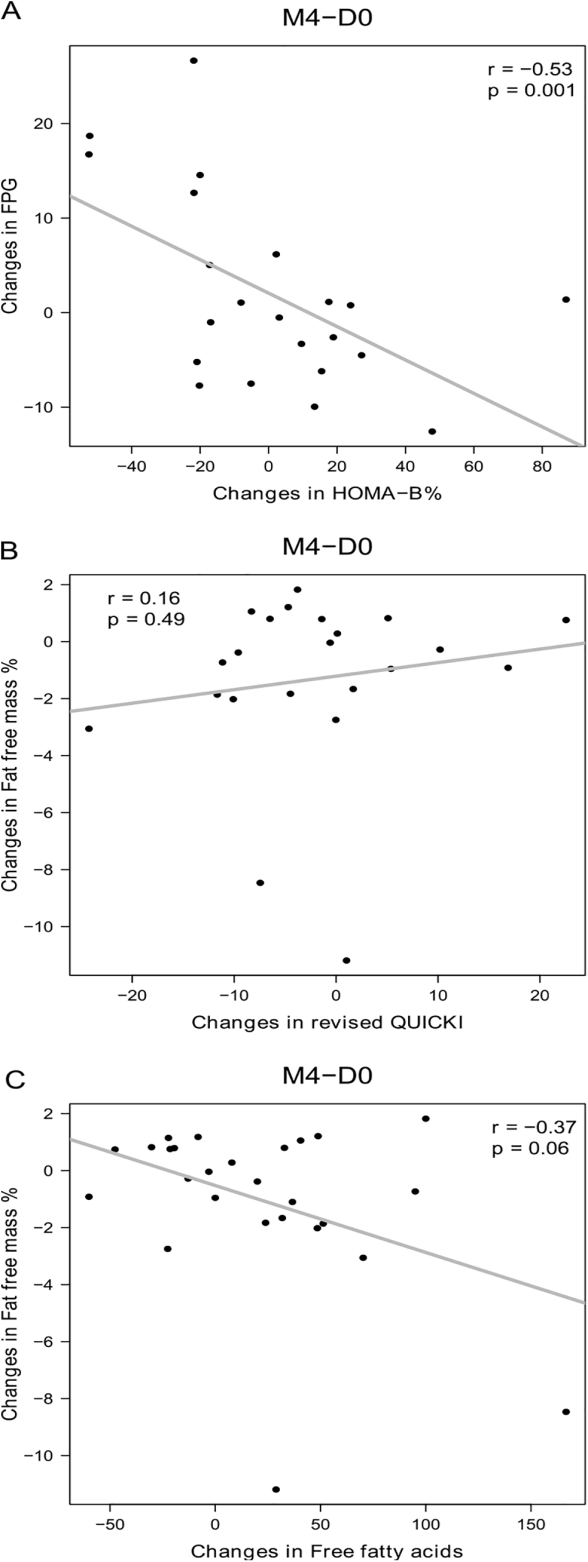
Correlations between changes in FPG or fat-free mass and other bioclinical parameters in the placebo group. A: Correlation between changes in FPG and insulin secretion estimated by HOMA-B%. B: Correlations between changes in fat-free mass and changes in insulin sensitivity estimated by revised QUICKI. C: Correlations between changes in fat-free mass and free fatty acids; N = 22 in A and B due to 4 missing data for plasma insulin, N = 26 subjects in C. Pearson correlations were used. D0: Day 0; M4: Month 4.

In the dietary supplement, the decrease in FPG was negatively correlated with increased insulin secretion (Fig 2A) and the increase in fat-free mass was associated with increased insulin sensitivity as measured by revised QUICKI (Fig 2B). The correlations remained significant after adjustment by gender, age and BMI: a) adjusted correlation between changes in FPG and changes in HOMA-B (p = 0.00005, r = -0.57), b) adjusted correlation between changes in fat-free mass and changes in revised QUICKI (p = 0.038, r = 0.31), c) adjusted correlation between changes in fat-free mass and changes in free fatty acids (p = 0.0006, r = -0.46). In the placebo group, changes in FPG were also negatively correlated to changes in insulin secretion (Fig 3A), whereas there was no significant correlation between changes in fat-free mass and changes in insulin sensitivity (Fig 3B).

### Other biological parameters

No within-group changes and no between-group differences were observed with respect to plasma triglycerides, total, HDL- or LDL-cholesterol, free fatty acids, plasma adipokines, inflammatory markers or adipose tissue metabolism *in vitro* (Table 3). In the dietary supplement group, but not in the placebo group, the increase in fat-free mass was negatively correlated with rise in plasma free fatty acid concentration (Figs 2C and 3C).

### Findings in the intention-to-treat population

Overall, the results concerning biological markers in the intention-to-treat population were similar to those seen in the per-protocol population (Table D in S1 File).

The rates of adverse events, as a whole, and serious adverse events did not differ significantly between the two groups (Table E in S1 File). None of the serious adverse events was considered to be related to the study treatment.

### Factors potentially predictive of decreased FPG in the dietary supplement group

#### 1. Associations between FPG changes and baseline characteristics

None of the following baseline characteristics was found to be associated with decreased FPG: sex, age, physical activity, total energy intake, macronutrient categories and adiposity markers. In contrast, the greatest decreases in FPG during dietary supplement treatment were significantly associated with high baseline FPG and with low baseline IL-6 values (Figures C and D in S1 File).

#### 2. Classification of subjects by their manifestation of changes in FPG and insulin secretion during dietary supplement intake

As shown in Fig 2A, points below the correlation line corresponded to subjects with the greatest decreases in FPG level in response to increased insulin secretion during dietary supplementation (good responders), whereas points above the line designated those whose FPG concentrations declined less than would be expected given the changes in their insulin secretion levels (poor responders). As regards the baseline characteristics of these two groups (Table F in S1 File), good responders had a lower mean age, a milder inflammatory state (based on plasma IL-6 level and IL-6 secretion by adipose tissue *in vitro*), and higher HDL levels. In addition, good responders tended to have higher FPG and lower plasma adiponectin levels at baseline compared to poor responders.

## Discussion

In view of the conflicting results of available clinical studies evaluating cinnamon [17–20] and chromium [23;37–39], the American Diabetes Association currently considers that there is insufficient evidence to support their use in subjects with diabetes [3;40]. Although modest, this randomized, double-blind study conducted in pre-diabetic subjects adds to the body of emerging evidence, showing that compared to placebo, treatment with a dietary supplement containing cinnamon, chromium and carnosine significantly decreased FPG levels. This beneficial effect was associated with a significant increase in fat-free mass. We included overweight or obese patients with impaired FPG who are considered to be at high risk of T2D and are assigned high priority for diabetes prevention [10]. The dietary supplement contained a cinnamon bark (*Cinnamomum cassia*) extract rich in polyphenol type-A polymers currently considered to be the bioactive components of cinnamon [41]. Concerning the type of cinnamon, *Cinnamomum zeylanicum*, however, is regarded to be more effective and safe than *Cinnamomum cassia* which is characterized by high concentrations of coumarins that may cause health risks [42;43]. For this reason, the European Food Safety Association (EFSA) advocated in 2008 against the regular use of *Cinnamomum cassia* as a supplement in diabetes [44]. However, data on the efficacy of *Cinnamomum zeylanicum* in humans are sparse [45] and no randomized double-blinded placebo-controlled clinical trials are available to establish the therapeutic efficacy and safety of *Cinnamomum zeylanicum* as a pharmaceutical agent. On the other hand, the extract of *Cinnamomum cassia* used in the present study, can be considered safe since the daily intake of two capsules contains 0.41 mg of coumarins which is well below the tolerable daily intake of 0.1 mg/kg body weight advised by the EFSA [44]. The dose and type of cinnamon tested in the present study were comparable to those selected in a previous study in pre-diabetic subjects with impaired FPG [21] as well as those with diabetes (42). Finally, the 4-month duration of treatment in the present study is one of the longest evaluated to date for cinnamon [17–20] and constitutes the recommended minimal duration in trials assessing the benefits of chromium [37].

Of note, there was a very slight elevation of HbA1c (within the normal range) in each of the dietary supplement and placebo groups without a significant difference between the two groups. We have no obvious reason explaining this difference, except only the observed slight non-significant increase in carbohydrate intake. In addition, the favorable effect of the dietary supplement on FPG, in the present study, was not associated with any benefit with respect to HbA1c. This is in agreement with a previous study in pre-diabetic subjects (21) and with a recent meta-analysis [19] in T2D patients. In the meta-analysis, the only two trials showing beneficial effect of cinnamon on HbA1c included diabetic patients with HbA1c levels greater than 8% [46;47]. In T2D subjects with controlled HbA1c levels, cinnamon failed to decrease HbA1c contrary to its effect on FPG [48]. Similar results were found in the pre-diabetic state with normal range of HbA1c [21]. It has been demonstrated that there is a poor agreement between FPG and low HbA1c values and that normal range HbA1c is less sensitive to variations in FPG [49]. It has been suggested that the effect of cinnamon is minimal when glucose control is closer to normal and that it exerts a significant effect in reducing FPG as the values slightly increase [50]. This observation is strengthened by the fact that the decrease in FPG observed in the dietary supplement group was negatively correlated with baseline FPG. Patients with the highest FPG level at baseline were those who benefited more from the dietary supplement showing thus the greatest reduction in FPG.

It may be argued that the 0.3 mmol/L difference between the changes in FPG achieved by the dietary supplement compared with placebo, in the absence of any difference in terms of HbA1c, is small and likely to have little impact on the future development of diabetes. Nevertheless, it is within the range observed in a previous study investigating the use of cinnamon alone in pre-diabetic subjects (0.5 mmol/L) [21]. Moreover, in intervention trials evaluating the effects of diet and exercise and/or various pharmacological agents, which showed a 30 to 70% decrease in the risk of diabetes in a much larger population with a much longer follow-up than in our study, the difference between the intervention and control groups with respect to change in FPG ranged from 0.2 mmol/L at 2.4-year follow-up to 0.9 mmol/L at 20-year follow-up [21;51–56]. Although, in diabetic patients, traditionally available therapeutic options reduce blood glucose concentrations, and subsequently improve pancreatic beta-cell function, none of the available anti-hyperglycemic agents changes the natural progression of T2D [57]. However, in pre-diabetic patients as in the present study, the modest decrease in FPG will prevent the development of diabetes with its associated micro- and macrovascular complications [58;59]. Moreover, epidemiological evidence shows that cardiovascular diseases might start at low plasma glucose levels, below the level defined for diabetes and even for impaired glucose tolerance [60;61]. Therefore, any minor lowering of FPG levels in non-diabetic subjects will be of benefit in both preventing the progression to diabetes and lowering the risk of cardiovascular diseases.

Interestingly, 23% of subjects in our dietary supplement group showed continued improvement in FPG level after treatment cessation, a percentage approaching consistency with the 35% to 50% rate of regression to normal glucose tolerance observed in some large intervention trials [55;56;62]. The decrease in FPG seen in the dietary supplement group was negatively associated with markers of insulin secretion. This correlation allowed identification of good responders, i.e. subjects showing an appropriate decrease in FPG in response to increased insulin secretion, and their tentative phenotype. In regards to the baseline characteristics of these two groups, the good responders had a lower mean age, a milder inflammatory state (based on plasma IL-6 level and IL-6 secretion by adipose tissue *in vitro*) and higher HDL levels. In addition, good responders tended to have higher FPG and lower plasma adiponectin levels at baseline compared to poor responders. Taken together, these findings may help to identify the subjects most likely to benefit from such dietary supplementation.

The question is raised whether the increased insulin secretion in the dietary supplement group could be of benefit since increasing insulin secretion capacity is one of the main targets of clinical perspective. Main therapies for diabetes aimed indeed at restoring insulin levels either by stimulating insulin secretion or improving insulin sensitivity. Therefore, any dietary supplements that may stimulate insulin secretion would be of promising benefit in controlling FPG levels in pre-diabetic subjects. However, we are aware that the estimation of insulin secretion by indirect surrogates is not the best way to evaluate insulin secretion. Further studies are needed to confirm this finding by direct methods as frequently sampled intravenous glucose tolerance test or hyperglycemic clamps.

Another interesting finding of the present study was that the use of the dietary supplement was associated with an increase in lean mass, although there was no difference *versus* placebo with respect to food intake, body weight, body mass index and lifestyle. This finding is consistent with that reported in the only previous study in subjects with impaired FPG which showed that 12-week treatment with cinnamon (500 mg/day) improved body composition by increasing lean mass and decreasing percentage of fat mass, using the same methods as ours to measure body composition [21]. In that study, the increase in lean mass (+1.4 kg) was in the same range as that we observed (+1.2 kg). Another study conducted in T2D patients showed that intake of a high dose of cinnamon (3 g/day) for eight weeks was associated with a decrease in percentage of fat mass [63], probably owing to an increase in fat-free mass as observed in our study. We believe this finding is important considering the role of lean mass in the long-term maintenance of metabolic rate, core body temperature, skeletal integrity, muscle strength, functional capacities, and prevention of sarcopenic obesity [64]. Since muscle constitutes the largest portion of insulin-sensitive tissue in the body, decrease in fat-free mass may be an important factor with regard to insulin resistance and risk of T2D [65–67]. Our study showed no evidence of any change in insulin resistance/sensitivity indices or free fatty acid level. However, in the dietary supplement, but not in the placebo group, there was a significant within-group improvement in insulin sensitivity (revised QUICKI and Disse index), and the increase in fat-free mass was associated with increased insulin sensitivity (as measured by revised QUICKI) and was negatively correlated with increased plasma free fatty acids. Several evidences support the involvement of free fatty acids in the development of skeletal muscle insulin resistance [68]. Cinnamon might increase fat-free mass by several mechanisms. In the dietary supplement group, increased insulin secretion might have increased glucose uptake that enabled glycogen to be synthetized and stored. Additionally, in the presence of an adequate supply of amino acids, insulin is anabolic in muscle and may increase muscle mass. Further researches are required to elucidate the underlying mechanisms.

As in the previous study investigating the benefits of cinnamon in pre-diabetic subjects [21], we did not find any difference between the dietary supplement and placebo groups with respect to lipid homeostasis. This might reflect the fact that the measured parameters were already normal at baseline. Chronic inflammation appears to be a central mediator of insulin resistance associated with obesity [69] or pre-diabetes [70–72]. In our study, no difference was seen between the treatment groups in terms of inflammatory markers, an unexpected finding considering the anti-inflammatory effect of cinnamon observed in experimental studies [16]. In contrast, we found that the dietary supplement had a greater effect on FPG in subjects with lower baseline plasma IL6. Furthermore, a milder inflammatory state at baseline was one of the characteristics of subjects identified as good responders to increased insulin secretion.

The question as to whether the other two components of the dietary supplement played a synergistic role in our observations is difficult to ascertain. The use of chromium in the treatment of T2D has been debated [39;73;74]. Two recent meta-analyses assessing the interest of chromium supplementation in the context of diabetes led to contradictory conclusions: The first one [38], based on 16 clinical studies, failed to show any benefit, whereas the second one [73] that included 22 studies concluded favorable effects of chromium supplementation on plasma glucose control in patients with diabetes. Chromium at doses of 200 to 1000 μg/day for 2 or 4 months was shown to reduce FPG and HbA1c in patients with T2D [23]. However, this benefit was not seen in pre-diabetic subjects [23]. Meta-analyses of trials in overweight or obese patients showed that chromium was associated with statistically significant reductions in body weight and percentage body fat, although these effects were considered to be of small magnitude and uncertain clinical relevance [24;37]. Importantly, several clinical studies showed that chromium supplementation increased lean body mass and decreased fat body mass in overweight or obese subjects [27;75;76]. In experimental studies, carnosine limited oxidative stress and inflammation, prevented protein cross-linking both in diabetic animals and in otherwise healthy aging animals, and appeared to possibly play a role in the regulation of blood glucose level via an effect on the autonomic nervous system [26;77;78]. However, no clinical data are available regarding the effect of carnosine on FPG or HbA1c.

In conclusion, a 4-month treatment with a dietary supplement containing cinnamon, chromium and carnosine decreased FPG and increased fat-free mass in overweight or obese pre-diabetic subjects. Whether the dietary supplement tested in this study can prevent the risk of T2D and related complications remains to be established in larger studies with longer treatment and follow-up durations, using clinical endpoints. Higher cinnamon doses as well as other sources of cinnamon extracts may also need to be tested.

## Supporting Information

S1 CONSORT Checklist CONSORT checklist. (DOC)

S1 Protocol Trial protocol. (DOC)

S2 Protocol French version of trial protocol. (DOC)

S1 File Table A in S1 File: Transformations used for each variable, Table B in S1 File: Baseline characteristics of complier subjects and subjects not considered in the per-protocol efficacy analysis; Table C in S1 File: Physical activity scores at baseline and after 4-month treatment in both the dietary supplement and placebo groups; Table D in S1 File: Efficacy analysis in the intention-to-treat population, Table E in S1 File: Adverse events, Table F in S1 File: Comparisons of baseline characteristics of good *vs*. poor responders to the dietary supplement, Figure A in S1 File: FPG (mmol/L) over time, Figure B in S1 File: Profiles of change in FPG (%) in the dietary supplement and placebo groups Figure C in S1 File: Relationship between change in FPG (%) and baseline FPG (mmol/L) in the dietary supplement and placebo groups, Figure D in S1 File. Relationship between change in FPG (%) and baseline plasma IL-6 level (pg/mL) in the dietary supplement and placebo groups. (DOC)

S1 Dataset (XLSX)

## References

[1] International Diabetes Federation. IDF Diabetes Atlas, 6th edn. Brussels, Belgium: International Diabetes Federation http://www.idf.org/diabetesatlas. 2013.

[2] American Diabetes Association. Diagnosis and classification of diabetes mellitus. Diabetes Care. 2010;33 Suppl 1: S62–9. 10.2337/dc10-S062 20042775 PMC2797383

[3] American Diabetes Association Standard of medical care in diabetes. Diabetes Care. 2014;37 Suppl 1: S14–80. 10.2337/dc14-S014 24357209

[4] Ryden L, Grant PJ, Anker SD, Berne C, Cosentino F, Danchin N, et al ESC Guidelines on diabetes, pre-diabetes, and cardiovascular diseases developed in collaboration with the EASD: the Task Force on diabetes, pre-diabetes, and cardiovascular diseases of the European Society of Cardiology (ESC) and developed in collaboration with the European Association for the Study of Diabetes (EASD). Eur Heart J. 2013;34(39):3035–87. 10.1093/eurheartj/eht108 23996285

[5] Garber AJ, Handelsman Y, Einhorn D, Bergman DA, Bloomgarden ZT, Fonseca V, et al Diagnosis and management of prediabetes in the continuum of hyperglycemia: when do the risks of diabetes begin? A consensus statement from the American College of Endocrinology and the American Association of Clinical Endocrinologists. Endocr Pract. 2008;14(7):933–46. 18996826 10.4158/EP.14.7.933

[6] Garber AJ, Abrahamson MJ, Barzilay JI, Blonde L, Bloomgarden ZT, Bush MA, et al American Association of Clinical Endocrinologists' comprehensive diabetes management algorithm 2013 consensus statement—executive summary. Endocr Pract. 2013;19(3):536–57. 10.4158/EP13176.CS 23816937 PMC4142590

[7] Tabak AG, Herder C, Rathmann W, Brunner EJ, Kivimaki M. Prediabetes: a high-risk state for diabetes development. Lancet. 2012;379(9833):2279–90. 10.1016/S0140-6736(12)60283-9 22683128 PMC3891203

[8] Moutzouri E, Tsimihodimos V, Rizos E, Elisaf M. Prediabetes: to treat or not to treat? Eur J Pharmacol. 2011;672(1–3):9–19. 10.1016/j.ejphar.2011.10.007 22020287

[9] Portero McLellan KC, Wyne K, Villagomez ET, Hsueh WA. Therapeutic interventions to reduce the risk of progression from prediabetes to type 2 diabetes mellitus. Ther Clin Risk Manag. 2014;10:173–88. 10.2147/TCRM.S39564 24672242 PMC3964168

[10] Paulweber B, Valensi P, Lindstrom J, Lalic NM, Greaves CJ, McKee M, et al A European evidence-based guideline for the prevention of type 2 diabetes. Horm Metab Res. 2010;42 Suppl 1:S3–36. 10.1055/s-0029-1240928 20391306

[11] Qin B, Nagasaki M, Ren M, Bajotto G, Oshida Y, Sato Y. Cinnamon extract (traditional herb) potentiates in vivo insulin-regulated glucose utilization via enhancing insulin signaling in rats. Diabetes Res Clin Pract. 2003;62(3):139–48. 14625128 10.1016/s0168-8227(03)00173-6

[12] Qin B, Dawson H, Polansky MM, Anderson RA. Cinnamon extract attenuates TNF-alpha-induced intestinal lipoprotein ApoB48 overproduction by regulating inflammatory, insulin, and lipoprotein pathways in enterocytes. Horm Metab Res. 2009;41(7):516–22. 19593846 10.1055/s-0029-1202813

[13] Qin B, Nagasaki M, Ren M, Bajotto G, Oshida Y, Sato Y. Cinnamon extract prevents the insulin resistance induced by a high-fructose diet. Horm Metab Res. 2004;36(2):119–25. 15002064 10.1055/s-2004-814223

[14] Sheng X, Zhang Y, Gong Z, Huang C, Zang YQ. Improved Insulin Resistance and Lipid Metabolism by Cinnamon Extract through Activation of Peroxisome Proliferator-Activated Receptors. PPAR Res. 2008;2008:581348 10.1155/2008/581348 19096709 PMC2602825

[15] Khan A, Safdar M, li Khan MM, Khattak KN, Anderson RA. Cinnamon improves glucose and lipids of people with type 2 diabetes. Diabetes Care. 2003;26(12):3215–8. 14633804 10.2337/diacare.26.12.3215

[16] Rafehi H, Ververis K, Karagiannis TC. Controversies surrounding the clinical potential of cinnamon for the management of diabetes. Diabetes Obes Metab. 2012;14(6):493–9. 10.1111/j.1463-1326.2011.01538.x 22093965

[17] Leach MJ, Kumar S. Cinnamon for diabetes mellitus. Cochrane Database Syst Rev. 2012;9:CD007170 10.1002/14651858.CD007170.pub2 22972104 PMC6486047

[18] Akilen R, Tsiami A, Devendra D, Robinson N. Cinnamon in glycaemic control: Systematic review and meta analysis. Clin Nutr. 2012;31(5):609–15. 10.1016/j.clnu.2012.04.003 22579946

[19] Allen RW, Schwartzman E, Baker WL, Coleman CI, Phung OJ. Cinnamon use in type 2 diabetes: an updated systematic review and meta-analysis. Ann Fam Med. 2013;11(5):452–9. 10.1370/afm.1517 24019277 PMC3767714

[20] Davis PA, Yokoyama W. Cinnamon intake lowers fasting blood glucose: meta-analysis. J Med Food. 2011;14(9):884–9. 10.1089/jmf.2010.0180 21480806

[21] Ziegenfuss TN, Hofheins JE, Mendel RW, Landis J, Anderson RA. Effects of a water-soluble cinnamon extract on body composition and features of the metabolic syndrome in pre-diabetic men and women. J Int Soc Sports Nutr. 2006;3:45–53. 10.1186/1550-2783-3-2-45 18500972 PMC2129164

[22] Roussel AM, Hininger I, Benaraba R, Ziegenfuss TN, Anderson RA. Antioxidant effects of a cinnamon extract in people with impaired fasting glucose that are overweight or obese. J Am Coll Nutr. 2009;28(1):16–21. 19571155 10.1080/07315724.2009.10719756

[23] Balk EM, Tatsioni A, Lichtenstein AH, Lau J, Pittas AG. Effect of chromium supplementation on glucose metabolism and lipids: a systematic review of randomized controlled trials. Diabetes Care. 2007;30(8):2154–63. 17519436 10.2337/dc06-0996

[24] Pittler MH, Stevinson C, Ernst E. Chromium picolinate for reducing body weight: meta-analysis of randomized trials. Int J Obes Relat Metab Disord. 2003;27(4):522–9. 12664086 10.1038/sj.ijo.0802262

[25] Martin J, Wang ZQ, Zhang XH, Wachtel D, Volaufova J, Matthews DE, et al Chromium picolinate supplementation attenuates body weight gain and increases insulin sensitivity in subjects with type 2 diabetes. Diabetes Care. 2006;29(8):1826–32. 16873787 10.2337/dc06-0254

[26] Reddy VP, Garrett MR, Perry G, Smith MA. Carnosine: a versatile antioxidant and antiglycating agent. Sci Aging Knowledge Environ. 2005;2005(18):e12. 10.1126/sageke.2005.18.pe12 15872311

[27] Anderson RA. Effects of chromium on body composition and weight loss. Nutr Rev. 1998;56(9):266–70. 9763876 10.1111/j.1753-4887.1998.tb01763.x

[28] Rizkalla SW, Prifti E, Cotillard A, Pelloux V, Rouault C, Allouche R, et al Differential effects of macronutrient content in 2 energy-restricted diets on cardiovascular risk factors and adipose tissue cell size in moderately obese individuals: a randomized controlled trial. Am J Clin Nutr. 2012;95(1):49–63. 10.3945/ajcn.111.017277 22170375

[29] Pellegrinelli V, Rouault C, Veyrie N, Clement K, Lacasa D. Endothelial cells from visceral adipose tissue disrupt adipocyte functions in a three-dimensional setting: partial rescue by angiopoietin-1. Diabetes. 2014;63(2):535–49. 10.2337/db13-0537 24130331

[30] Ribiere C, Jaubert AM, Sabourault D, Lacasa D, Giudicelli Y. Insulin stimulates nitric oxide production in rat adipocytes. Biochem Biophys Res Commun. 2002;291(2):394–9. 11846418 10.1006/bbrc.2002.6444

[31] Matthews DR, Hosker JP, Rudenski AS, Naylor BA, Treacher DF, Turner RC. Homeostasis model assessment: insulin resistance and beta-cell function from fasting plasma glucose and insulin concentrations in man. Diabetologia. 1985;28(7):412–9. 3899825 10.1007/BF00280883

[32] Antuna-Puente B, Disse E, Faraj M, Lavoie ME, Laville M, Rabasa-Lhoret R, et al Evaluation of insulin sensitivity with a new lipid-based index in non-diabetic postmenopausal overweight and obese women before and after a weight loss intervention. Eur J Endocrinol. 2009;161(1):51–6. 10.1530/EJE-09-0091 19429699

[33] Stunkard AJ, Messick S. The three-factor eating questionnaire to measure dietary restraint, disinhibition and hunger. J Psychosom Res. 1985;29(1):71–83. 3981480 10.1016/0022-3999(85)90010-8

[34] Baecke JA, Burema J, Frijters JE. A short questionnaire for the measurement of habitual physical activity in epidemiological studies. Am J Clin Nutr. 1982;36(5):936–42. 7137077 10.1093/ajcn/36.5.936

[35] Lemon J. Plotrix: a package in the red light district of R. R-News. 2006;6(4):8–12.

[36] Pinheiro J, Bates D, DebRoy S, Sarkar D. R Development Core Team. nlme: Linear and Nonlinear Mixed Effects Models. R package version 3.1–103. Vienna: R Foundation for Statistical Computing 2012.

[37] Onakpoya I, Posadzki P, Ernst E. Chromium supplementation in overweight and obesity: a systematic review and meta-analysis of randomized clinical trials. Obes Rev. 2013;14(6):496–507. 10.1111/obr.12026 23495911

[38] Bailey CH. Improved meta-analytic methods show no effect of chromium supplements on fasting glucose. Biol Trace Elem Res. 2014;157(1):1–8. 10.1007/s12011-013-9863-9 24293356

[39] Kleefstra N, Houweling ST, Bilo HJ. Effect of chromium supplementation on glucose metabolism and lipids: a systematic review of randomized controlled trials. Diabetes Care. 2007;30(9):e102 17726181 10.2337/dc07-1015

[40] Evert AB, Boucher JL, Cypress M, Dunbar SA, Franz MJ, Mayer-Davis EJ, et al Nutrition therapy recommendations for the management of adults with diabetes. Diabetes Care. 2014;37 Suppl 1:S120–S143. 10.2337/dc14-S120 24357208

[41] Anderson RA, Broadhurst CL, Polansky MM, Schmidt WF, Khan A, Flanagan VP, et al Isolation and characterization of polyphenol type-A polymers from cinnamon with insulin-like biological activity. J Agric Food Chem. 2004;52(1):65–70. 14709014 10.1021/jf034916b

[42] Ranasinghe P, Jayawardana R, Galappaththy P, Constantine GR, de Vas GN, Katulanda P. Efficacy and safety of 'true' cinnamon (Cinnamomum zeylanicum) as a pharmaceutical agent in diabetes: a systematic review and meta-analysis. Diabet Med. 2012;29(12):1480–92. 10.1111/j.1464-5491.2012.03718.x 22671971

[43] Ranasinghe P, Perera S, Gunatilake M, Abeywardene E, Gunapala N, Premakumara S, et al Effects of Cinnamomum zeylanicum (Ceylon cinnamon) on blood glucose and lipids in a diabetic and healthy rat model. Pharmacognosy Res. 2012;4(2):73–9. 10.4103/0974-8490.94719 22518078 PMC3326760

[44] European Food Safety Authority (EFSA). Scientific Opinion of the Panel on Food Additives, Flavourings, Processing Aids and Materials in Contact with Food on a request from the European Commission on Coumarin in flavourings and other food ingredients with flavouring properties. 2008. Report No.: *793*. 10.2903/j.efsa.2008.729 PMC10193617 37213834

[45] Ranasinghe P, Pigera S, Premakumara GA, Galappaththy P, Constantine GR, Katulanda P. Medicinal properties of 'true' cinnamon (Cinnamomum zeylanicum): a systematic review. BMC Complement Altern Med. 2013;13:275 10.1186/1472-6882-13-275 24148965 PMC3854496

[46] Crawford P. Effectiveness of cinnamon for lowering hemoglobin A1C in patients with type 2 diabetes: a randomized, controlled trial. J Am Board Fam Med. 2009;22(5):507–12. 10.3122/jabfm.2009.05.080093 19734396

[47] Akilen R, Tsiami A, Devendra D, Robinson N. Glycated haemoglobin and blood pressure-lowering effect of cinnamon in multi-ethnic Type 2 diabetic patients in the UK: a randomized, placebo-controlled, double-blind clinical trial. Diabet Med. 2010;27(10):1159–67. 10.1111/j.1464-5491.2010.03079.x 20854384

[48] Mang B, Wolters M, Schmitt B, Kelb K, Lichtinghagen R, Stichtenoth DO, et al Effects of a cinnamon extract on plasma glucose, HbA, and serum lipids in diabetes mellitus type 2. Eur J Clin Invest. 2006;36(5):340–4. 16634838 10.1111/j.1365-2362.2006.01629.x

[49] Incani M, Sentinelli F, Perra L, Pani MG, Porcu M, Lenzi A, et al Glycated hemoglobin for the diagnosis of diabetes and prediabetes: Diagnostic impact on obese and lean subjects, and phenotypic characterization. J Diabetes Investig. 2015;6(1):44–50. 10.1111/jdi.12241 25621132 PMC4296702

[50] Medagama AB, Bandara R, Abeysekera RA, Imbulpitiya B, Pushpakumari T. Use of Complementary and Alternative Medicines (CAMs) among type 2 diabetes patients in Sri Lanka: a cross sectional survey. BMC Complement Altern Med. 2014;14:374 10.1186/1472-6882-14-374 25280877 PMC4201716

[51] Pan XR, Li GW, Hu YH, Wang JX, Yang WY, An ZX, et al Effects of diet and exercise in preventing NIDDM in people with impaired glucose tolerance. The Da Qing IGT and Diabetes Study. Diabetes Care. 1997;20(4):537–44. 9096977 10.2337/diacare.20.4.537

[52] Li G, Zhang P, Wang J, Gregg EW, Yang W, Gong Q, et al The long-term effect of lifestyle interventions to prevent diabetes in the China Da Qing Diabetes Prevention Study: a 20-year follow-up study. Lancet. 2008;371(9626):1783–9. 10.1016/S0140-6736(08)60766-7 18502303

[53] Tuomilehto J, Lindstrom J, Eriksson JG, Valle TT, Hamalainen H, Ilanne-Parikka P, et al Prevention of type 2 diabetes mellitus by changes in lifestyle among subjects with impaired glucose tolerance. N Engl J Med. 2001;344(18):1343–50. 11333990 10.1056/NEJM200105033441801

[54] Knowler WC, Barrett-Connor E, Fowler SE, Hamman RF, Lachin JM, Walker EA, et al Reduction in the incidence of type 2 diabetes with lifestyle intervention or metformin. N Engl J Med. 2002;346(6):393–403. 11832527 10.1056/NEJMoa012512 PMC1370926

[55] Gerstein HC, Yusuf S, Bosch J, Pogue J, Sheridan P, Dinccag N, et al Effect of rosiglitazone on the frequency of diabetes in patients with impaired glucose tolerance or impaired fasting glucose: a randomised controlled trial. Lancet. 2006;368(9541):1096–105. 16997664 10.1016/S0140-6736(06)69420-8

[56] DeFronzo RA, Tripathy D, Schwenke DC, Banerji M, Bray GA, Buchanan TA, et al Pioglitazone for diabetes prevention in impaired glucose tolerance. N Engl J Med. 2011;364(12):1104–15. 10.1056/NEJMoa1010949 21428766

[57] Heine RJ, Diamant M, Mbanya JC, Nathan DM. Management of hyperglycaemia in type 2 diabetes: the end of recurrent failure? BMJ. 2006;333(7580):1200–4. 17158386 10.1136/bmj.39022.462546.80 PMC1693603

[58] Stratton IM, Adler AI, Neil HA, Matthews DR, Manley SE, Cull CA, et al Association of glycaemia with macrovascular and microvascular complications of type 2 diabetes (UKPDS 35): prospective observational study. BMJ. 2000;321(7258):405–12. 10938048 10.1136/bmj.321.7258.405 PMC27454

[59] Haffner SM, Lehto S, Ronnemaa T, Pyorala K, Laakso M. Mortality from coronary heart disease in subjects with type 2 diabetes and in nondiabetic subjects with and without prior myocardial infarction. N Engl J Med. 1998;339(4):229–34. 9673301 10.1056/NEJM199807233390404

[60] Bragg F, Li L, Smith M, Guo Y, Chen Y, Millwood I, et al Associations of blood glucose and prevalent diabetes with risk of cardiovascular disease in 500 000 adult Chinese: the China Kadoorie Biobank. Diabet Med. 2014;31(5):540–51. 10.1111/dme.12392 24344928 PMC4114560

[61] Gerstein HC, Punthakee Z. Dysglycemia and the Risk of Cardiovascular Events Evidence-Based Cardiology. Wiley-Blackwell; 2009 p. 179–89.

[62] Chiasson JL, Josse RG, Gomis R, Hanefeld M, Karasik A, Laakso M. Acarbose for prevention of type 2 diabetes mellitus: the STOP-NIDDM randomised trial. Lancet. 2002 6 15;359(9323):2072–7. 12086760 10.1016/S0140-6736(02)08905-5

[63] Vafa M, Mohammadi F, Shidfar F, Sormaghi MS, Heidari I, Golestan B, et al Effects of cinnamon consumption on glycemic status, lipid profile and body composition in type 2 diabetic patients. Int J Prev Med. 2012;3(8):531–6. 22973482 PMC3429799

[64] Miller CT, Fraser SF, Levinger I, Straznicky NE, Dixon JB, Reynolds J, et al The effects of exercise training in addition to energy restriction on functional capacities and body composition in obese adults during weight loss: a systematic review. PLoS One. 2013;8(11):e81692 10.1371/journal.pone.0081692 24409219 PMC3884087

[65] Srikanthan P, Hevener AL, Karlamangla AS. Sarcopenia exacerbates obesity-associated insulin resistance and dysglycemia: findings from the National Health and Nutrition Examination Survey III. PLoS One. 2010;5(5):e10805 10.1371/journal.pone.0010805 22421977 PMC3279294

[66] Dominguez LJ, Barbagallo M. The cardiometabolic syndrome and sarcopenic obesity in older persons. J Cardiometab Syndr. 2007;2(3):183–9. 17786082 10.1111/j.1559-4564.2007.06673.x

[67] Atlantis E, Martin SA, Haren MT, Taylor AW, Wittert GA. Inverse associations between muscle mass, strength, and the metabolic syndrome. Metabolism. 2009;58(7):1013–22. 10.1016/j.metabol.2009.02.027 19394973

[68] Martins AR, Nachbar RT, Gorjao R, Vinolo MA, Festuccia WT, Lambertucci RH, et al Mechanisms underlying skeletal muscle insulin resistance induced by fatty acids: importance of the mitochondrial function. Lipids Health Dis. 2012;11:30 10.1186/1476-511X-11-30 22360800 PMC3312873

[69] Theuma P, Fonseca VA. Inflammation, insulin resistance, and atherosclerosis. Metab Syndr Relat Disord. 2004;2(2):105–13. 10.1089/met.2004.2.105 18370641

[70] Haffner SM. Insulin resistance, inflammation, and the prediabetic state. Am J Cardiol. 2003;92(4A):18J–26J. 12957323 10.1016/s0002-9149(03)00612-x

[71] Festa A, Hanley AJ, Tracy RP, D'Agostino R Jr., Haffner SM. Inflammation in the prediabetic state is related to increased insulin resistance rather than decreased insulin secretion. Circulation. 2003;108(15):1822–30. 14517163 10.1161/01.CIR.0000091339.70120.53

[72] Lu Q, Tong N, Liu Y, Li N, Tang X, Zhao J, et al Community-based population data indicates the significant alterations of insulin resistance, chronic inflammation and urine ACR in IFG combined IGT group among prediabetic population. Diabetes Res Clin Pract. 2009;84(3):319–24. 10.1016/j.diabres.2009.03.002 19442860

[73] Suksomboon N, Poolsup N, Yuwanakorn A. Systematic review and meta-analysis of the efficacy and safety of chromium supplementation in diabetes. J Clin Pharm Ther. 2014;39(3):292–306. 10.1111/jcpt.12147 24635480

[74] Abdollahi M, Farshchi A, Nikfar S, Seyedifar M. Effect of chromium on glucose and lipid profiles in patients with type 2 diabetes; a meta-analysis review of randomized trials. J Pharm Pharm Sci. 2013;16(1):99–114. 23683609 10.18433/j3g022

[75] Crawford V, Scheckenbach R, Preuss HG. Effects of niacin-bound chromium supplementation on body composition in overweight African-American women. Diabetes Obes Metab. 1999;1(6):331–7. 11225649 10.1046/j.1463-1326.1999.00055.x

[76] Kim CW, Kim BT, Park KH, Kim KM, Lee DJ, Yang SW, et al Effects of short-term chromium supplementation on insulin sensitivity and body composition in overweight children: randomized, double-blind, placebo-controlled study. J Nutr Biochem. 2011;22(11):1030–4. 10.1016/j.jnutbio.2010.10.001 21216583

[77] Nagai K, Niijima A, Yamano T, Otani H, Okumra N, Tsuruoka N, et al Possible role of L-carnosine in the regulation of blood glucose through controlling autonomic nerves. Exp Biol Med (Maywood). 2003;228(10):1138–45. 14610252 10.1177/153537020322801007

[78] Nagai K, Tanida M, Niijima A, Tsuruoka N, Kiso Y, Horii Y, et al Role of L-carnosine in the control of blood glucose, blood pressure, thermogenesis, and lipolysis by autonomic nerves in rats: involvement of the circadian clock and histamine. Amino Acids. 2012;43(1):97–109. 10.1007/s00726-012-1251-9 22367578

