# Correction: A Dietary Supplement Containing Cinnamon, Chromium and Carnosine Decreases Fasting Plasma Glucose and Increases Lean Mass in Overweight or Obese Pre-Diabetic Subjects: A Randomized, Placebo-Controlled Trial

**DOI:** 10.1371/journal.pone.0145315

**Published:** 2015-12-14

**Authors:** Yuejun Liu, Aurélie Cotillard, Camille Vatier, Jean-Philippe Bastard, Soraya Fellahi, Marie Stévant, Omran Allatif, Clotilde Langlois, Séverine Bieuvelet, Amandine Brochot, Angèle Guilbot, Karine Clément, Salwa W. Rizkalla

The affiliation for the ninth author is incorrect. Séverine Bieuvelet is not affiliated with #5 but with #6 Groupe PiLeJe, 75015, Paris, France.

## References

[1] Liu Y, Cotillard A, Vatier C, Bastard J-P, Fellahi S, Stévant M, et al (2015) A Dietary Supplement Containing Cinnamon, Chromium and Carnosine Decreases Fasting Plasma Glucose and Increases Lean Mass in Overweight or Obese Pre-Diabetic Subjects: A Randomized, Placebo-Controlled Trial. PLoS ONE 10(9): e0138646 doi:10.1371/journal.pone.0138646 26406981 10.1371/journal.pone.0138646 PMC4583280

