# Supplementary material for: A Dietary Supplement Containing Cinnamon, Chromium and Carnosine Decreases Fasting Plasma Glucose and Increases Lean Mass in Overweight or Obese Pre-Diabetic Subjects: A Randomized, Placebo-Controlled Trial
Source: PLoS One. 2015 Sep 25;10(9):e0138646. doi: 10.1371/journal.pone.0138646 (PMC4583280; doi:10.1371/journal.pone.0138646)
Supplement: S1 Protocol — (DOC) [file pone.0138646.s002.doc]

*CLINICAL STUDY ON EFFICACY*

***Effect of dietary supplement Glycabiane on glycaemic control in prediabetic overweight or OBESE subjects.***

Short title: *Glycabiane and glycaemic control in prediabetic overweight subjects.*

**Version:** 1.2 **date of: 20/04/2012**

**Sponsor**

**Laboratoire PILEJE**

37 Quai de Grenelle, Tour Pollux, 75738 Paris Cedex 15, France

PARIS Trade and Companies Register no. 490 450 452

E-mail: s.bieuvelet@pileje.com

**Principal investigator**:

Prof. Karine Clément

Department of Nutrition

Groupe Pitié Salpêtrière

47-83 boulevard de l’hôpital

Pavillon Husson Mourler

75651 Paris Cedex 13, France

Phone: +33 (0)1.42.17.79 19

E-mail: karine.clement@psl.aphp.fr

**Co-investigator**:

Dr. Salwa Rizkalla

INSERM U872 (Eq 7), CRNH[[1]](#footnote-2) Ile de France (Paris Metropolitan area)

Department of Nutrition

Bâtiment Benjamin Delessert

Hôpital Pitié Salpêtrière

83 Bd de l’Hôpital

75013 Paris, France

Phone: +33 (0)1.42.17.79.81

E-mail: salwa.rizkalla@psl.aphp.fr

**Place of conduct of study**:

INSERM U872 (Eq 7), CRNH Ile de France (Paris Metropolitan area)

Department of Nutrition

Bâtiment Benjamin Delessert

Hôpital Pitié Salpêtrière

83 Bd de l’Hôpital

75013 Paris, France

**Synopsis**

| Title | Effect of the dietary supplement Glycabiane, on glycaemic control in prediabetic overweight subjects. |
| --- | --- |
| Sponsor | PILEJE – 37 quai de Grenelle – 75015 Paris, France |
| Coordinating Investigator | Prof. Karine Clément  Department of Nutrition  Groupe Pitié Salpêtrière  47-83 boulevard de l’hôpital  Pavillon Husson Mourler  75651 Paris Cedex 13, France |
| Protocol version | Version no. 1.2 |
| Rationale/context | Prediabetes is a condition in which glycaemia is higher than normal, but without being high enough to justify the diagnosis of type 2 diabetes (i.e. fasting glycaemia of 7.0 mmol/L or higher). All persons with prediabetes will not develop type 2 diabetes, but a good proportion of them will develop it. The risk of developing type 2 diabetes increases with age. A change in lifestyle, including an increase in physical activity of moderate intensity and a balanced diet with a hypocaloric diet, in case of overweight and/or obesity, are the basis of management to prevent or to delay progression to type 2 diabetes. However, such changes in lifestyle are not always easy to implement in the long-term. Different nutritional intervention studies have demonstrated that cinnamon and chromium can help to fight insulin resistance, with or without a change in lifestyle. Therefore, the combination cinnamon/chromium may help to control glycaemia in prediabetic subjects and thus to limit the occurrence of diabetes and of its micro and macrovascular complications, as well as therapeutic escalation often observed in such patients. |
| Primary Objective | To study the influence of 4 month supplementation with Glycabiane on fasting glycaemia in prediabetic, overweight subjects. |
| Secondary Objectives | To study the influence of 4 month supplementation with Glycabiane on:  - glycosylated haemoglobin (HbA1c)  - lipid profile (Total cholesterol, LDL-c, HDL-c, TG, etc.)  - markers of inflammation: circulating levels of IL6 and CRPus  - weight, BMI, waistline, ratio of height over and hip circumference and total fatty mass  - diastolic and systolic blood pressure  - insulin resistance  - size of adipocytes  - inflammatory status of adipose tissue (measurement of secretion of cytokine IL-6 and of adipokine, adiponectin)  - sensitivity to insulin of adipose tissue by study of phosphorylation of Akt and of secretion of adiponectin  To evaluate the safety of 4 month supplementation with Glycabiane |
| Primary endpoint | Fasting glycaemia in prediabetic overweight subjects before and after nutritional intervention |
| Secondary endpoints | To study the influence of 4-month supplementation with Glycabiane on lipid profile (Total cholesterol, LDL-c, HDL-c, TG, etc.), levels of glycosylated haemoglobin, anthropometric characteristics and on the biology of adipose tissue (size of adipocytes, inflammatory status, sensitivity to insulin.  To evaluate safety of 4-month supplementation with Glycabiane  To evaluate quality of life |
| Methodology/Study design | A single centre, randomised, double-blind, placebo-controlled study |
| Criteria for inclusion of subjects | Men and women 25-65 years of age:  - prediabetic: 1.00 g/L ≤ fasting glycaemia < 1.26 g/L,  - overweight or obesity (BMI>25),  - with normal renal and hepatic function tests,  - covered by the French social security system,  - sufficiently cooperative to comply with requirements of the study,  - patients with immunocompetent status (no known HIV or hepatitis C infection),  - use of an effective method of contraception by women of childbearing potential. |
| Criteria for Non-Inclusion of Subjects | - thyroid disorder - hypogonadism - history of a musculo-skeletal, auto-immune or neurological disorder - Hepatitis C, HIV - Medical therapy for thyroid disease, hypoglycaemia or use anti-coagulant therapy - pregnant women or breastfeeding mothers - subject under measure of legal protection - subject who received fees of €4,500 as compensation for participation in clinical trials during the last 12 months. This information is to be verified by the investigator, since subjects are compensated in the study - history of allergy to one of the components of the investigational product - contra-indication to use of xylocaine as a local anaesthetic |
| Number of Patients | 50 subjects: 25 subjects per group |
| Duration of Study | Duration of the inclusion period: 6 months  Duration of participation for each patient: 6 months  Total duration of study: 24 months |
| Expected findings | This first randomised, double-blind, placebo-controlled study of a new combination cinnamon/chromium represents important hope in order to regulate fasting glycaemia in prediabetic overweight subjects with a nutritional product and with a physiological dose in order to limit progression of prediabetes to type 2 diabetes. |

TABLE OF CONTENTS

[I. Study rationale 7](#__RefHeading___Toc394046426)

[A. Name and description of the disorder 7](#__RefHeading___Toc394046427)

[B. Treatments or management already existing and their limits 8](#__RefHeading___Toc394046428)

[1. Change in lifestyle and diet 8](#__RefHeading___Toc394046429)

[2. Pharmacological interventions 8](#__RefHeading___Toc394046430)

[3. Nutritional intervention 10](#__RefHeading___Toc394046431)

[C. The new proposed management 16](#__RefHeading___Toc394046432)

[D. Expected benefits and/or foreseeable risks for persons 16](#__RefHeading___Toc394046433)

[II. Protection of persons 17](#__RefHeading___Toc394046434)

[A. Ethical rationale of the study 17](#__RefHeading___Toc394046435)

[B. Ethical and regulatory conditions 17](#__RefHeading___Toc394046436)

[C. Committee for Protection of Persons (CPP) 18](#__RefHeading___Toc394046437)

[D. AFSSAPS (French Agency for the Safety of Health Products) 18](#__RefHeading___Toc394046438)

[E. Insurance and financing 19](#__RefHeading___Toc394046439)

[F. Modalities for collection of consent from patients 19](#__RefHeading___Toc394046440)

[G. Compensation 20](#__RefHeading___Toc394046441)

[III. Objectives 20](#__RefHeading___Toc394046442)

[A. Primary objective 20](#__RefHeading___Toc394046443)

[B. Secondary objectives 20](#__RefHeading___Toc394046444)

[IV. Experimental design 21](#__RefHeading___Toc394046445)

[A. Study design 21](#__RefHeading___Toc394046446)

[B. Research category: 21](#__RefHeading___Toc394046447)

[C. Statement of the experimental design 21](#__RefHeading___Toc394046448)

[V. Evaluation endpoints 23](#__RefHeading___Toc394046449)

[A. Primary endpoint 23](#__RefHeading___Toc394046450)

[B. Secondary endpoints: 23](#__RefHeading___Toc394046451)

[VI. Screening of persons 23](#__RefHeading___Toc394046452)

[A. Participating studied population: 23](#__RefHeading___Toc394046453)

[1. Duration of participation of persons in the study: 23](#__RefHeading___Toc394046454)

[2. Description of participating persons: 23](#__RefHeading___Toc394046455)

[3. Number of persons to include: 24](#__RefHeading___Toc394046456)

[B. Recruitment and withdrawals from study 24](#__RefHeading___Toc394046457)

[1. Recruitment 24](#__RefHeading___Toc394046458)

[2. Withdrawals from study 24](#__RefHeading___Toc394046459)

[3. Exclusion period and participation in another study 25](#__RefHeading___Toc394046460)

[VII. Practical conduct of study 25](#__RefHeading___Toc394046461)

[A. Detailed description of procedures practiced on persons: 25](#__RefHeading___Toc394046462)

[B. Description of general logistical organisation of study 27](#__RefHeading___Toc394046463)

[C. Provisional study schedule (Flow-Chart) 28](#__RefHeading___Toc394046464)

[VIII. Studied product 28](#__RefHeading___Toc394046465)

[A. Description of the studied product 28](#__RefHeading___Toc394046466)

[1. Glycabiane 28](#__RefHeading___Toc394046467)

[2. Placebo 29](#__RefHeading___Toc394046468)

[B. Dispensing and compliance 29](#__RefHeading___Toc394046469)

[IX. Medical devices 29](#__RefHeading___Toc394046470)

[X. Collection of human biological samples 31](#__RefHeading___Toc394046471)

[XI. study safety 31](#__RefHeading___Toc394046472)

[A. DEFINITIONS RELATING TO SAFETY 31](#__RefHeading___Toc394046473)

[B. investigators’ role 32](#__RefHeading___Toc394046474)

[Evaluation of severity/causal relation of adverse events 32](#__RefHeading___Toc394046475)

[C. sponsor’s role 33](#__RefHeading___Toc394046476)

[D. follow-up of patients in case of a serious adverse event 34](#__RefHeading___Toc394046477)

[E. Independent monitoring committee 34](#__RefHeading___Toc394046478)

[XII. Data collection and processing 34](#__RefHeading___Toc394046479)

[A. data collection 34](#__RefHeading___Toc394046480)

[B. Description of channel of data management before data processing 34](#__RefHeading___Toc394046481)

[Data collection 34](#__RefHeading___Toc394046482)

[Computerised data processing 35](#__RefHeading___Toc394046483)

[XIII. Statistical analysis of data 36](#__RefHeading___Toc394046484)

[Number of subjects necessary 36](#__RefHeading___Toc394046485)

[XIV. Cnil 37](#__RefHeading___Toc394046486)

[XV. Archiving 37](#__RefHeading___Toc394046487)

[XVI. Communication 38](#__RefHeading___Toc394046488)

[XVII. Substantial changes to the protocol 38](#__RefHeading___Toc394046489)

[XVIII. Confidentiality 38](#__RefHeading___Toc394046490)

[A. Access to data 38](#__RefHeading___Toc394046491)

[B. Source data 38](#__RefHeading___Toc394046492)

[C. Confidentiality of data 39](#__RefHeading___Toc394046493)

[D. Registration in the national file of persons who are subjects in biomedical research 39](#__RefHeading___Toc394046494)

[XIX. Quality assurance 39](#__RefHeading___Toc394046495)

[A. Commitment by investigators and sponsor 39](#__RefHeading___Toc394046496)

[B. Quality Assurance 40](#__RefHeading___Toc394046497)

[C. Quality control 40](#__RefHeading___Toc394046498)

[D. Case report forms 40](#__RefHeading___Toc394046499)

[XX. bibliographical references 41](#__RefHeading___Toc394046500)

[XXI. Appendixes 46](#__RefHeading___Toc394046501)

[A. Appendixe 1: Lettre d’information version 1.2 46](#__RefHeading___Toc394046502)

[B. Appendixe 2: Consentement éclairé version 1.3 46](#__RefHeading___Toc394046503)

[C. Appendixe 3: Copie de l’attestation d’assurance 46](#__RefHeading___Toc394046504)

[D. Appendixe 4: Autorisation du lieu de recherche 46](#__RefHeading___Toc394046505)

[E. Appendixe 5: Copies de l’avis favorable du CPP datant du 20/12/2010 et de l’Afssaps datant du 2/11/2010 46](#__RefHeading___Toc394046506)

[F. Appendixe 6: Questionnaires TFEQ et d’activité physique 46](#__RefHeading___Toc394046507)

[G. Appendixe 7: Carnet alimentaire 46](#__RefHeading___Toc394046508)

[H. Appendixe 8: Annonce « Métro » 46](#__RefHeading___Toc394046509)

[I. Appendixe 9: Déclaration d’Helsinki 46](#__RefHeading___Toc394046510)

[J. Appendixe 10: BPC 46](#__RefHeading___Toc394046511)

[A. Appendixe 1: Lettre d’information version 1.2 47](#__RefHeading___Toc394046512)

[B. Appendixe 2: Consentement éclairé version 1.3 48](#__RefHeading___Toc394046513)

[C. Appendixe 3: Copie de l’attestation d’assurance 49](#__RefHeading___Toc394046514)

[D. Appendixe 4: Autorisation du lieu de recherche 50](#__RefHeading___Toc394046515)

[E. Appendixe 5: Copies de l’avis favorable du CPP datant du 20/12/2010 et de l’Afssaps datant du 2/11/2010 51](#__RefHeading___Toc394046516)

[F. Appendixe 6: Questionnaires TFEQ et d’activité physique 52](#__RefHeading___Toc394046517)

[G. Appendixe 7: Carnet alimentaire 53](#__RefHeading___Toc394046518)

[H. Appendixe 8: Annonce « Métro » 54](#__RefHeading___Toc394046519)

[I. Appendixe 9: Déclaration d’Helsinki 55](#__RefHeading___Toc394046520)

[J. Appendixe 10: BPC 56](#__RefHeading___Toc394046521)

# Study rationale

## Name and description of the disorder

Type 2 diabetes is a disorder whose aetiopathogenesis and pathophysiology are complex. This disorder develops unquestionably in a subject with a genetic predisposition and is promoted by inappropriate lifestyle. Type 2 diabetes is characterised by hyperglycaemia resulting from deficiency of the action of insulin or of insulin secretion or of both of these two factors together (1). This hyperglycaemia is associated, in varying degrees and with mechanisms which are still incompletely elucidated, with long-term complications, in particular affecting the eyes, kidney, nerves, heart and arteries. This disorder is marked by a long period of asymptomatic progression and typically occurs in subjects starting with the age 50 years, in particular, in subjects who are overweight or who have a family history of the same disorder.

The absence of symptoms in type 2 diabetes is such so that it is very difficult to determine the prevalence of this disorder. Nevertheless, it is a disorder which continues to progress and the number of affected patients increases each year worldwide, including in the developing countries.

Worldwide, type 2 diabetes accounts for 90% of diabetic patients and this figure should rise from 135 to 300 million patients between 1995 and 2025 (2).

Currently, the prevalence of type 2 diabetes is higher in the industrialised countries and will remain so, but the expected increase in this prevalence, between now and 2025, is 171% in the developing countries and 41% in the industrialised countries. Countries which have the highest number of diabetics are India, China and the USA and this proportion will remain as such for the next 20 years (3).

Based on requests for reimbursement for antidiabetic medicinal products, the prevalence of diabetes under treatment has been estimated at 3.95% in 2007, i.e. 2,500,000 persons. It is higher in categories of older subjects, with a maximum of 18.2% in men 75 to 79 years of age, and of 13.2% in women of the same age. Wide regional variations exist in subjects of equivalent age with higher rates in the French overseas’ departments (between 6 and 7%) and in the North and North-East of France (4.4% in Lorraine, 4.5% in Alsace and Champagne-Ardennes, 4.7% in Picardie, and 4.8% in the Nord-Pas-De-Calais region).

This prevalence is rising rapidly, increasing by 5.7% per year on average (4). It results from the combination of two factors: the effects of aging of the population and that of a rise in prevalence of obesity in France (5).

Currently, it can be considered as established that type 2 diabetes, except in rare cases, is a disorder characterised by the combination of insulin resistance (decreased sensitivity to the action of insulin) and a deficiency in insulin secretion by pancreatic beta cells. More exactly, the onset of diabetes appears as a process which develops in two phases (6):

first, insulin resistance, but with normal glycaemia, maintained at the cost of “compensatory” hyperinsulinaemia; followed by a shift to diabetes strictly speaking when the pancreas no longer can provide the quantity of insulin necessary to maintain homeostasis of insulin dependent metabolism.

The first “prediabetic” period generally is accompanied by various potentially atherogenic or thrombogenetic metabolic anomalies probably derived from the combination of insulin resistance plus hyperinsulinaemia and are combined under the name of “metabolic syndrome”.

Therefore, “prediabetes” is an intermediate phase between normal glucose homeostasis and confirmed type 2 diabetes, and includes two clinical entities: moderate fasting hyperglycaemia (MFH) and glucose intolerance (GI).

Abnormal fasting glycaemia is defined as fasting glycaemia of 1.0 g/l, but < 1.26 g/l according to the IDF (International Diabetes Federation) in partnership with the AHA (American Heart Association), the NHLBI (National Heart, Lung and Blood Institute), and the International Atherosclerosis Society and the International Association for the study of Obesity (6 bis).

Glucose intolerance is defined as glycaemia ≥1.40 g/l and < 2 g/l, 2 hours after a 75 g oral load of glucose.

## Treatments or management already existing and their limits

### Change in lifestyle and diet

Three prospective studies have demonstrated the effectiveness of weight loss and of physical activity on risk reduction of diabetes in patients with glucose intolerance.

**A US study of a DPP (diabetes prevention programme)**, conducted on 3,234 subjects with glucose intolerance compared the effect of Metformin (a hypoglycaemic pharmacological treatment) to that of changes in lifestyle (reduction of caloric intake + physical exercise). Results made it possible to establish that weight loss (of 5 to 7%) by means of changes in diet (decrease in intake of saturated fat (<10% of total caloric intake, an increase in intake of fibre (of 15 g/day) reinforced by physical exercise (at least 30 min/day) made it possible to decrease the risk of diabetes by 58% in three years versus 31% with Metformin (7)

**The Da Qing study in China** (1997), conducted on 577 subjects with glucose intolerance showed that dietary intervention and/or physical exercise followed during 6 years decreased the risk of diabetes overall by 39% (8).

**A Finish diabetes prevention study (DPS)** conducted on 522 subjects with insulin resistance showed that weight loss of 5% obtained by following an intensive dietary programme (decrease in intake of saturated fat and increase in intake of fibre) and exercise made it possible to reduce the incidence of diabetes by 58%.

In 2007, a meta-analysis [10] evaluated the effectiveness of various interventions, change in lifestyle (diet and exercise) and use of medicinal products on the risk of developing type 2 diabetes. Changes to lifestyle, diet and physical exercise designed to prevent or to reduce obesity clearly decreased the risk of diabetes, without having adverse effects.

### Pharmacological interventions

Medicinal products (metformin and acarbose) which reduce the uptake of glucose do not change the progression of the disease, but only delay its onset.

Furthermore, all pharmacological interventions have adverse events which must be taken into account.

#### Metformin

In the US study of the DPP, one third of patients with glucose intolerance took metformin during three years. Progression to type 2 diabetes seems to have decreased by 31% compared to a control group. This medicinal product appeared as more effective in subgroups of patients under 60 years of age and with a BMI greater than 30 kg/m². Discontinuation of the medicinal product resulted in a loss of efficacy of prevention of over 75%. The main adverse events reported were nausea, diarrhoea and abdominal pain which were found in over 25% of subjects. This medicinal product is contra-indicated in case of renal impairment (creatinine clearance < 30 ml/min).

#### Acarbose

Acarbose is an oral hypoglycaemic agent whose mode of action is to delay absorption of glucose from the intestine by inhibition of alphaglucosidase. In the STOP-NIDDM study, use of acarbose significantly reduced the risk of progression to type 2 diabetes by 25% in patients with glucose intolerance and this was true, whatever the subject’s age or BMI (9). This medicinal product also seems to decrease cardiovascular complications in subjects with prediabetes. Its utility is limited by occurrence of flatulence and diarrhoea associated with its regular use. In the STO-NIDDM study, 25% of subjects treated discontinued their treatment as the result of adverse events associated with the medicinal product.

#### Thiazolidinediones

The DREAM study sought to demonstrate in over 1,500 prediabetic patients the utility either of a medicinal product generally used for treatment of heart failure or of hypertension (Ramipril) or of an oral hypoglycaemic agent (rosiglitazone, a medicinal product that stimulates PPAR gamma, which leads to a redistribution of visceral fat or a shift from the liver to muscle and which increases transport of glucose in muscle between other mechanisms of action of this complex medicinal product). Although it slightly decreased glycaemia, ramipril did not decrease the occurrence of diabetes. Rosiglitazone decreased the risk of progression from glucose intolerance to type 2 diabetes by 62% compared to a control group. Rosiglitazone appeared to be more effective in patients with a large waistline. However, in 5 years follow-up, only 20% of subjects progressed to type 2 diabetes. Furthermore, a small increase in cardiovascular risk and a definite increase in risk of distal bone fractures seems to limit, at least for the time being, use of rosiglitazone as prophylaxis in young patients. Moreover, this therapeutic modality is associated with a constant weight gain (10).

#### Orlistat

This medicinal product is a lipase inhibitor used to treat obesity which improves glycaemia by stimulating weight loss. Its possible mechanisms of action may include improvement in sensitivity to insulin, slowing of incomplete digestion of lipids, a decrease in plasma postprandial non-esterified fatty acids, a reduction in visceral adipose tissue and stimulation of secretion of glucagon-like peptide-1 in the small intestine (11). The main adverse events reported are oily stool, faecal incontinence and urgent diarrhoea: frequent bowel movements or urgency, bloating and abdominal pain, such as colic and flatulence.

### Nutritional intervention

#### Cinnamon in prevention of hyperglycaemia

Cinnamon is a spice traditionally used in some Asian countries in treatment of diabetes. Since 1990, different *in vitro* and *in vivo* animal studies have demonstrated its utility in the regulation of glucose metabolism (12-16). In adipocyte cell lines studied *in vitro*, cinnamon in extract form stimulated the uptake of glucose and synthesis of glycogen by stimulating phosphorylation of the insulin receptor and glycogen synthase (14). Cinnamon extract can also modulate the expression of PPAR transcription factor and of its target genes, including LPL, CD-36 and GLUT-4 which are involved in metabolism of lipids (17). *In vivo* in rats, cinnamon extract stimulated the use of glucose in a dose-dependent manner by stimulation of phosphorylation of the beta subunit of the insulin receptor, that of IRS-1 (Insulin Receptor Substrate-1) and the combination IRS-1 with PI3K (Phosphatidyl Inositol 3 Kinase) (15,18).

One of the active substances in cinnamon extract may be a methylhydroxychalcone polymer that can mimic insulin and can produce “insulin-like” effects (14), in particular OPC-A (type A polyphenol polymer, water-soluble) (12).

In humans, 6 clinical studies and one meta-analysis on the effects of cinnamon on glucose metabolism have been published. The majority sought to determine the effect of cinnamon in patients with type 2 diabetes (19-24) (table). Only one study investigated persons with metabolic syndrome (25), while two other studies were conducted on healthy subjects (26; 27). Results of studies on type 2 diabetes are debatable, some of them concluded in an effect of cinnamon on fasting glycaemia, HbA1c and possibly lipid profile, while others did not report any significant effect (table). It should be noted that all studies contained bias in methodology. These studies often were conducted on a limited number of subjects, biological data at baseline were not always standardised, duration of supplementation did not always make it possible to observe changes in levels of HbA1c, etc. Furthermore, cinnamon supplementation was always different, whether in terms of dose or in terms of duration of supplementation. Yet, the effects of cinnamon appear to depend both on the dose ingested the duration of supplementation, and on initial carbohydrate-fat parameters of patients. Other randomised, double-blind, placebo-controlled studies therefore will be necessary to determine the exact role of cinnamon on carbohydrate-fat parameters in patients with type 2 diabetes.

A double-blind, placebo-controlled study determined the effects of supplementation with a water-soluble cinnamon extract^15^ on fasting glycaemia in men and women with metabolic syndrome or prediabetes. After adjustment for age, fasting glycaemia, blood pressure, and levels of usual physical activity, 12 subjects ingested 500 mg per day of an aqueous extract of cinnamon containing 1% OPCA and 10 received a placebo during 12 weeks. Results showed a significant decrease in fasting glycaemia (- 8.4%: 116.3+/-12.8 mg/dl at start vs. 106.5+/-20.1 mg/dl at end of the study) in subjects who ingested the cinnamon extract. Subjects also had a decrease in systolic blood pressure (-3.8%) as well as in their body fatty mass (-0.7%), while their lean mass increased by 1.1%.

Recently, a team led by Salomon evaluated the effect of ingestion of 3 g of cinnamon per day during 14 days on glucose tolerance and sensitivity to insulin in 8 healthy men 25 ± 1 years of age with a BMI of 24± 0.7. 14 subjects received either Placebo or cinnamon extract alternately during 14 days. Supplementation with Placebo was continued for 5 days after the 14 days’ supplementation. Glucose tolerance tests were conducted at D0, D1, D14, D16, D18 and D20. Ingestion of cinnamon significantly reduced glycaemic response to the glucose tolerance test at D1 (-13.1±6.3% vs. D0, p<0.05) and D14 (-5.5±8.1% vs. D0, p=0.09). Ingestion of cinnamon also reduced the insulin response at D14 (-27.1±6.2% vs. D0, p <0.05) and sensitivity to insulin (p<0.05 at D14 vs. D0). These positive effects ceased after discontinuation of supplementation suggesting that cinnamon can improve glycaemic control and sensitivity to insulin, but that these effects are reversible (28). Lastly, another study on 15 healthy subjects showed a beneficial effect of ingestion of 3 g of cinnamon mixed with rice cake on postprandial serum insulin (vs. rice cake without cinnamon). No effect was found with 1 g of cinnamon and no effect was found on glycaemia (29).

In conclusion, convincing scientific data are still lacking to conclude in an effect of cinnamon on glycaemia, although it seems that cinnamon has a slight hypoglycaemia-inducing effect. Regarding levels of HbA1c, the effect on this parameter appears negligible, but other randomised placebo-controlled clinical studies are necessary to evaluate its real impact.

| **Bibliographical References** | **Study design and number of subjects (N)** | **Target population** | **Intervention** | **Results** | **Comments** |
| --- | --- | --- | --- | --- | --- |
| Khan A. et al. *Diabetes Care* 26:3215-3218, 2003(30) | A randomised, placebo-controlled clinical trial  N=60 | Men and women with type 2 diabetes (T2D) 52.2±6.32 years of age | 6 groups of 10 subjects:  Gr 1: 1 g cinnamon/d  Gr 2: 2 g cinnamon/d  Gr 3: 3 g cinnamon/d  Gr 4: 1 g placebo/d  Gr 5: 2 g placebo/d  Gr 6: 3 g placebo/d Supplementation during 40 days, and then 20 days with no supplement | Fasting glycaemia was significantly decreased by 18 to 29%, triglycerides by 23 to 30%, total cholesterol by 13 to 26% and LDL cholesterol by 10 to 24% in response to cinnamon supplementation. No significant change in levels of HDL cholesterol was observed. Levels of glucose and triglycerides rose slightly after discontinuation of treatment, but remained below baseline values. Those of total cholesterol and LDL cholesterol continued to decrease. No adverse event was reported. | The study was conducted on Pakistani patients with poorly controlled diabetes and whose baseline serum glucose levels were significantly higher than those generally observed in other clinical studies on patients with type 2 diabetes. Generally, diabetic patients have better glycaemic control. Therefore, it is likely that the results observed in this study overestimate the positive effects of cinnamon. Furthermore, the study is limited by the sample size (a total of 60 subjects: 10 per group) and the absence of standardisation of the 6 groups in terms of dietary intake. |
| Mang B. et al. *Eur J Clin Invest* 2006; 36(5):340-344(31) | A prospective, randomised, double-blind, placebo-controlled study N=79 | German men and women T2D | 2 groups:  Gr 1: 112 mg aqueous extract of C. cassia equivalent to 1 g of cinnamon, three times a day during 4 months  Gr 2: placebo three times a day during 4 months | Supplementation resulted in a significant decrease in fasting glycaemia (-10.3% ± 13.2%) in diabetic subjects who received the aqueous extract of cinnamon compared to those who took the placebo (-3.37 ± 14.2%) (p=0.046).  No significant change in levels of HbA1c was observed. No adverse event was reported. | Although the authors concluded in a moderate effect of cinnamon on fasting glycaemia in patients with poorly controlled type 2 diabetes, it was noted that this study contained different limiting factors. First, the sample size (n=65). Then, the frequency of measurements of glycaemia. It would have been advisable to make measurements closer together to confirm the trend observed. |
| Vanschoonbeek K. et al.*J. Nutr.* 136:977-980. 2006(32) | A placebo-controlled, non-randomised study  N=25 | T2D, post-menopausal women 62.9+/1.5 years of age with a BMI of 30.4 +/- 0.9 kg/m2 | 2 groups:  Gr 1 (n=13): 1 g of cinnamon/d during 6 weeks  Gr 2 (n=12): 1 g of placebo/d during 6 weeks | Results after 6 weeks of supplementation, no significant difference between the groups that received supplementation and placebo was observed in levels of HbA1c nor in fasting glycaemia. | Certain biases in methodology can be underlined. First, this trial was an open-label, non-randomised study. Next, the study involved solely post-menopausal women with type 2 diabetes and not the entire population of patients with type 2 diabetes and on a small number of subjects (a total of 25). Lastly, duration of the study (6 weeks) perhaps was not sufficient to observe changes in HbA1c. Moreover, initial fasting glycaemia of patients included in this study was less than that observed in other studies. |
| Altschuler JA. et al. *Diabetes Care* 30:813-816, 2007.(33) | A randomised, double-blind, placebo-controlled study  N=75 | Adolescents with type 1 diabetes | Ingestion of 1 g of cinnamon or placebo/d during 90 days | No significant difference was observed in levels of glycosylated haemoglobin (8.8 vs. 8.7, p=0.88) nor in serum insulin between start and end of supplementation. | Absence of the production of insulin in patients with type 1 diabetes may be at the origin of the absence of the effect of cinnamon. In fact, *in vitro* and *in vivo* studies in rodents suggest that cinnamon may act by stimulating endogenous production of insulin. Furthermore, it is possible that subjects ingested an insufficient quantity of cinnamon. Studies on patients with type 2 diabetes in fact show an interesting effect of cinnamon with high doses of about 1.5 to 3 g cinnamon per day. Duration of supplementation can also be cast into doubt since renewal of red blood cells requires 120 days and supplementation lasted only 90 days. |
| Blevins SM et al. *Diabetes Care* 30:2236-2237, 2007. (34) | A randomised, double-blind, placebo-controlled study  N=57 | US patients with type 2 diabetes, mean age 63 years of age. | 2 groups:  Gr 1 (n=29): 1 g of cinnamon/d during 3 months  Gr 2 (n=28): 1 g of placebo/d during 3 months | No significant difference was observed in fasting glycaemia and in HbA1c. |  |
| Baker WL. et al. *Diabetes Care* 31:41-43, 2008.(35) | A meta-analysis of clinical trials on effect of cinnamon in T2D subjects  N=282 | Patients with type 2 diabetes | 5 clinical trials | Results showed that ingestion of cinnamon did not significantly change HbA1c, fasting glycaemia or plasma lipid parameters. The authors concluded that cinnamon had no significant effect on carbohydrate-fat parameters of patients with types 1 and 2 diabetes. | In the 5 clinical trials selected (N=282), on corresponded to a non-randomised trial, while another had as the target adolescents with type 1 diabetes. The 3 other randomised, placebo-controlled clinical trials included a total of 296 patients. The doses of cinnamon ingested and duration of supplementation varied from one study to another, as well as the target population (more or less well-controlled diabetes, etc.) |
| Ziegenfuss TN. et al. J Int Soc Sports Nutr 3: 45-53, 2006.(36) | A randomised, double-blind, placebo-controlled study  N=22 | Prediabetic subjects with multiple metabolic changes | 2 groups:  Gr 1 (n=12): 500 mg of cinnamon extract titrated with 1% OPCA/d during 3 months  Gr 2 (n=10): 500 mg of placebo/d during 3 months | The results showed a significant decrease in fasting glycaemia (-8.4%: 116.3+/-12.8 mg/dl at start vs. 106.5+/-20.1 mg/dl at end of study) in subjects who ingested cinnamon extract, p<0.05. Subjects also had a decrease in systolic blood pressure (-3.8%) as well as in body fatty mass (-0.7%) while lean mass increased by 1.1%. |  |

#### Chromium and carbohydrate metabolism

Chromium is an essential trace element for carbohydrate and lipid metabolism. In animals, it is needed to maintain normal glucose tolerance. In humans, there are several clinical signs associated with a chromium deficiency: elevated glycaemia and insulin levels, hypertriglyceridaemia and hypercholesterolaemia, and also neurological and memory disorders (patient under parenteral nutrition).

Diabetic patients have 33% lower serum chromium concentrations than those of normal subjects, but on the contrary, have higher urinary excretion (100%) (37). Such excretion perhaps is related to glomerular hyperfiltration or to renal lesions (a process known to occur in patients with type 1 diabetes, but debated in those with NIDDM). In patients with retinopathy and other complications associated with diabetes, chromium concentrations seem to be lower than those of diabetics who do not have these associated disorders. Furthermore, measurement of chromium in the lens of the eye in patients with retinopathy revealed levels similar to those observed in the elderly and lower than in controls (38).

An inverse relationship between serum chromium and diabetic disease is not causal, but underlines the essential feature of chromium in management of glycaemic homeostasis. It is very likely that, without being the sole cause of the clinical signs observed, chromium deficiency may worsen or accelerate the course of the disease. Moreover, different factors can affect absorption and excretion of chromium, i.e. stress (via cortisol), ingestion of rapid acting sugars, and physical activity can induce increased loss of chromium. Therefore, patients with glycaemic dysfunction may be more exposed to alteration of the metabolism of chromium. Consequently, sufficient chromium intake to restore “normal” levels in the blood may be beneficial as suggested by studies of supplementation.

Chromium seems to act as a regulator of glycaemic homeostasis: in patients with reactive hypoglycaemia, chromium (200 µg of chloride/day) makes it possible to increase glycaemia and to reduce hypoglycaemic symptoms (39). In diabetic patients, with abnormal postprandial hyperglycaemia, chromium makes it possible to reduce glycaemia and to modulate serum insulin. On the contrary, in patients who do not have an alteration of glycaemia, chromium supplementation remains ineffective.

Studies of chromium supplementation have reported variable results on fasting glycaemia and glucose tolerance. No dose-effect study has been conducted in humans, but it would appear that studies where intake of chromium is less than 5 µmol/day provide more variable results than those using higher doses. Furthermore, the form in which chromium is supplied seems to be important, with the picolinate derivative being more effective than the chloride derivative (40; 41). A review by Mertz (41) showed that only a few studies conducted did not conclude in the beneficial effects of chromium in diabetics, but that in studies in which a dose of trivalent chromium >400 µg a day was provided during several months (min. 2-4 months) the latter reduced insulin resistance and improved glucose tolerance. In particular, a recent study on supplementation for 2 to 4 months in 180 diabetic subjects and 83 control subjects showed a reduction in fasting and postprandial glycaemia (-20 and 15%). Similarly, fasting and postprandial serum insulin were decreased (-25-30%). Therefore, there was regulation of glycaemia and an increase in glucose tolerance in patients with NIDDM (42). In 2007, a large meta-analysis classified 41 clinical trials (N=1,198) which evaluated the effective different forms of chromium on carbohydrate-lipid metabolism. Doses tested ranged from 200 to 1,000 µg a day during 2 to 26 weeks. Among these 41 trials, 14 (n=431) followed patients with type 2 diabetes with baseline HbA1c ranging from 7.0% to 10.2%. Results showed that supplementation with chromium picolinate and beer yeast at doses ranging from 200 to 1,000 µg a day during 2 to 26 weeks reduced the levels of HbA1c by 0.6% (95% CI: -0.9% to -0.2%) and of fasting glycaemia by 1 mmol/L (95% CI -1.4 to -0.5)(43). However, it is important to note that in this meta-analysis, half of the studies included were of poor quality and used different forms and doses of chromium in populations which may ingest chromium in their diet. Out of the 41 studies identified, no adverse event was reported.

The mechanism of action of chromium in glycaemic homeostasis is poorly elucidated. First, it seems that chromium forms with nicotinic acid (vitamin B3), amino acids (cysteine, glycine, glutamine) and with glutathione a complex called “Glucose Tolerance Factor”, which in vitro has beneficial effects on utilisation of glucose and binding of insulin to its receptor (44). In humans, it was not clearly identified, but lack of nicotinic acid may decrease the beneficial effect of chromium on glycaemia (45). Furthermore, chromium may act by increasing insulin efficacy: chromium supplementation results in increased binding of insulin to its peripheral receptor which may be due to an increase in the number of receptors (42).

#### Carnosine

Carnosine (or L-carnosine) is a natural beta-alanyl-L-histidine dipeptide primarily found in tissues with a long lifespan, such as skeletal muscle, the brain and the lens (more than 20 mM in muscle). It forms as the result of the action of carnosine synthetase which produces a bridge between the two amino acids alanine and histidine. Its concentrations in tissues result from a balance between synthesis by carnosine synthetase and inactivation by carnisinase. High levels of carnosine exist in cells with a long lifespan (such as neurons).

Carnosine has recognised antioxidant and anti-glycating properties as well as direct activity of limiting protein-sugar binding: or so-called “anti-crosslinking” activity (46).

Carnosine reacts with sugars such as glucose, galactose and dihydroxyacetone to form glycosylated carnosine (non-toxic for the body which eliminates it). The most reactive of these three sugars is dihydroxyacetone: carnosine reacts with it more quickly than with lysine. Furthermore, carnosine inactivates glycosylated proteins by dihydroxyacetone. Thus, carnosine makes it possible to reduce glycation of proteins and formation of AGEs (Advanced Glycation End products) by improving its recognition by their receptor. Furthermore, a very recent study (47) demonstrated that carnosine (20 mM) can intervene in the deglycation of Schiff bases (transglycation) catalysing the reaction of deglycation and generating amine groups.

Boldyrev demonstrated that 30% to 70% of carnosine comes from the diet (depending on the quantity of different amino acids in food) and that of pure L-carnosine is over 70%. Absorption occurs mainly in the small intestine (jejunum, but not in the ileum) (48). A study in rats demonstrated that carnosine supplementation (0.0875% of the diet) associated with vitamin E (50 ppm) increased carnosine concentrations in the liver by 1.5 times (49). The same was true in mice. A study on diabetic mice (diabetes induced by streptozocin) supplemented with 0.5 g/L or 1 g/L of carnosine in their drinking water during 4 weeks revealed an increase in carnosine concentrations, both in the plasma, in the heart and in the liver, and a decrease in quantity of dialdehyde malone (an end-stage product of highly reactive lipoperoxydation) produced (50).

Therefore, carnosine supplementation may make it possible to increase plasma and tissue concentrations of carnosine and thus may decrease glycation. To date, no clinical trial has been published on the effect of L-carnosine supplementation on glycation of proteins in patients with type 2 diabetes.

## The new proposed management

Several studies have demonstrated the benefit of changes in lifestyle on progression of glucose intolerance or from prediabetes to type 2 diabetes. Nevertheless, it must be noted that such changes in lifestyle (such as close monitoring of diet and regular exercise) are often difficult to implement in the long-term. Difficulties can be explained by a lack of conviction, motivation and diligence in some patients, but also by reasons of inability or impossibility for other patients to follow such recommendations (in the elderly, existence of a cardiac disorder, inappropriate physical conditions).

When changes in lifestyle are poorly followed or are not adequate to normalise glycaemia, it sometimes can be necessary to initiate nutritional supplementation and/or medical therapy.

Medical therapies are often accompanied by non-negligible adverse events and only delay the time to onset of type 2 diabetes.

Data from the literature on cinnamon and chromium provide evidence in support of the effect of each of these dietary components on carbohydrate metabolism in overweight subjects with prediabetes. Carnosine may reduce glycation of proteins.

The dietary supplement Glycabiane provides simultaneous intake of OPC-A in cinnamon, chromium and carnosine, in proportions which should make it possible to obtain a significant effect on carbohydrate metabolism in subjects with prediabetes.

Intake of chromium in the form of Guanylor® is especially useful because it may enable better intestinal absorption in the form of yeast traditionally used in dietary supplements. A comparative kinetic study of absorption of chromium in the form of Guanylor®-Cr or of yeast high in chromium has been conducted by Pileje Laboratory in non-deficient vigilant rats. Results showed that administration of Guanylor*®* Cr resulted in a significantly higher increase in plasma concentration of chromium than that observed with yeast high in chromium (See technical dossier, unpublished confidential data). Furthermore, the quantity of chromium eliminated in the faeces was significantly lower with Guanylor® Cr than with yeast high in chromium suggesting improved absorption of chromium provided in Guanylor®-Cr. Concomitantly, ChalCinn® is a cinnamon extract specifically developed and selected by Pileje. Especially high in OPC-A (at least 5 mg of OPC-A per 100 mg extract) and with reduced coumarin content (< 15 ppm to prevent anti-coagulant effects) and essential oils (< 2% to retain the status of a dietary supplement), it should enable supplementation in the long-term with a significant effect on carbohydrate metabolism.

Therefore, Glycabiane is formulated to optimise the effects of chromium and of OPC-A in cinnamon on carbohydrate metabolism in overweight prediabetic subjects.

The aim of this clinical trial conducted in the CRNH of Ile-de-France (Metropolitan Paris), Department of Nutrition at Pitié Salpêtrière, is to evaluate the effect of nutritional supplementation with the dietary supplement Glycabiane on fasting glycaemia in prediabetic overweight subjects.

## Expected benefits and/or foreseeable risks for persons

Participation in this study can lead to progression of knowledge by physicians on understanding the prevention of diabetes and elevation of glycaemia, but the benefit for participating subjects cannot be evaluated precisely, since this is the subject of the study. Current knowledge makes it possible to formulate the hypothesis of the utility of Glycabiane in terms of regulation of fasting glycaemia in prediabetic subjects who ingest Glycabiane and thus limit the progression to type 2 diabetes (with micro and macrovascular complications). In the exceptional case where, for a given subject, the study might demonstrate a risk to health, appropriate data would be communicated to the investigator who would provide them in a relevant manner to the person concerned, in the setting of respect for persons and medical secrecy.

- **Risks related to treatments**

The studied product is a dietary supplement which comes in capsule form containing cinnamon extract, carnosine and chromium. It is already marketed under the trade name Glycabiane by PiLeJe since 2008.

Since 2010, when the collection of adverse events related to ingestion of the product was set up, nutrition vigilance has not recorded any unexpected serious adverse event and has been informed of only a single case of an adverse event whose causal relation with the product was doubtful.

- No cases in 2010
- 1 case in 2011: gastric burning (causal relationship: doubtful)
- No cases in 2012
- **Risks related to metabolic investigations.**

***Suctioning of adipose tissue by needle puncture:*** This procedure was performed under local anaesthesia, with the possible unpleasant effect being an ecchymosis generally of small size which is reabsorbed in a few days, and, in rare cases, a small haematoma (small collection of blood), reabsorbed in a few days and that can require application of an alcohol dressing. The dose used for local anaesthesia (Xylocaine 1% without adrenaline) is non-toxic and very rarely allergenic (amino amide allergy)

****DEXA:*** It is hazardless and provides radiation of about 70-140 kV (less than one chest X-ray). The examination lasts about ten minutes.

- Study constraints.

Regulation in terms of human clinical trials prohibits a subject who participates in a research study to participate at the same time participate in other experimental studies. If the subject accepts to participate in this study, he/she must not participate in any other clinical trial during the same period.

*DEXA: dual energy X-ray absorptometry

# Protection of persons

## Ethical rationale of the study

This study is a single centre, national, randomised, double-blind, placebo-controlled study of efficacy.

## Ethical and regulatory conditions

The study will be conducted in compliance with French regulations in force, in particular, conditions relating to biomedical research of the Public Health Code, article L 1121-1 and following, of laws on Bioethics, law on Data Processing, Computer Files and Freedoms (and, in particular, methodology of reference MR-001 relating to biomedical research – appendix 6, of the Declaration of Helsinki (appendix 9), as well as the present study.

The investigator agrees to conduct the study in compliance with ethical and regulatory conditions. He/she is conscious that all documents, as well as all data relating to the study may be audited and inspections performed in compliance with professional secrecy and without being opposed by medical secrecy. The investigator recognises that results of this study are the property of Pileje Laboratory, sponsor of the study.

## Committee for Protection of Persons (CPP)

Before implementation of the study, the sponsor will submit the projected study for an opinion to the CPP of Ile de France VI (metropolitan Paris) and will provide all the necessary information: research protocol, information form (appendix 1) and consent form (appendix 2) and all other relevant documents that have to be presented to the Committee.

The trial can start only after Pileje has received a favourable opinion without reservation from the CPP concerning the protocol submitted. This opinion will include the study title and number assigned by the sponsor, the documents examined, as well as the date of its examination and list of members of the CPP who participated in the deliberations.

The sponsor will inform the CPP of all substantial changes to the protocol and of all serious or unexpected adverse events and of new facts occurring during the study and which would probably affect the safety of persons who are subjects in it, in compliance with the sponsor’s procedure described in the present protocol.

## AFSSAPS[[2]](#footnote-3)

Prior to set up of a clinical trial, not involving a health product, the applicant must do the following:

1. Obtain a national registration number for the study from Afssaps (ID-RCB number);

2. Send an application for authorisation of a clinical trial dossier (AEC) to Afssaps.

Evaluation of the dossier by Afssaps consists of:

- an examination of its completeness (technico-regulatory admissibility),

- its technical evaluation

Biomedical research studies not involving a health product mentioned in article L. 5311-1 of the PHC[[3]](#footnote-4) are subject to an implicit authorisation regimen: for such studies, upon expiry of the time for evaluation assigned to Afssaps (60 days), absence of any objection expressd by the latter signifies its authorisation.

Whenever Afssaps requires further information and if the applicant sends it in within the assigned time period, the trial is authorised implicitly if Afssaps has not notified its decision before expiry of the 60 day time of evaluation. In this case, the sponsor can request from Afssaps, upon expiry of the legal time period of 60 days, the granting of a certificate confirming that the subject of the application has been authorised by Afssaps.

According to conditions of article L. 1123-9 of the PHC, after the start of the study, any substantial change to it at the sponsor’s initiative must obtain a favourable opinion from the committee for the protection of persons (CCP) prior to its implementation and authorisation from the competent authority (authorisation for a substantial change or ASC)

During evaluation of the application for authorisation of the study or an opinion on it, Afssaps and the CPP mutually exchange information on requests for further information, objections to set up of the study, and requests for changes that each one formulates to the applicant. For this purpose Afssaps and the CPP mutually exchange a copy of letters that they have sent to the applicant.

According to conditions of article L. 1123-9 of the PHC, after the start of the study, any substantial change made at the sponsor’s initiative must obtain, prior to its implementation, a favourable opinion from the committee for protection of persons (CPP) and authorisation from the competent authority (authorisation for a substantial change or ASC). Non-substantial changes are those which do not have significant impact on any aspect of the study whatsoever. Such changes do not have to be submitted to Afssaps, neither for authorisation, nor for information.

The sponsor must immediately report to Afssaps, as well as to the CPP:

- **all suspected unexpected serious adverse events** (USAEs)that occurred in France in the trial concerned

- any **new finding** or any new data that may jeopardise the safety of persons who are subjects in this study or that have an impact on conduct of the study

Periodically

- a **report each semester** containing:

- a list of all suspected unexpected serious adverse events (USAEs )that occurred in other trials conducted in France with the same product, procedure or method, as well as those that occurred outside of France in the concerned study;

- a concise synthesis prepared by the sponsor underlining the principal problems of safety raised.

- the annual **safety report**.

Furthermore, the sponsor communicates:

- to the CPP concerned: all further information requested by it on cases of patient deaths notified

- to Afssaps: new facts brought to its knowledge after the end of the study.

## Insurance and financing

Pileje, in its capacity as the sponsor, has taken out insurance for the duration of the study covering its civil responsibility, from the HDI-Gerling Industries insurance company, address 111, rue de Longchamp, 75115 Paris, France, under the policy number (1680) 90712, in compliance with French legal and regulatory conditions for biomedical research (insurance certificate relating to the present protocol in appendix 3 of the latter).

The insurance certificate related to the present protocol comprises appendix 3.

Financing of the study is assured by Pileje.

## Modalities for collection of consent from patients

Written informed consent must be obtained from all persons who are subjects in a biomedical research study, by the investigator who is registered in the Order of Doctors, declared in the capacity of investigator to the sponsor, before any procedure is performed in the setting of the research study and whatever this procedure, in compliance with the Public Health Code, article L1122-1.

Information will be given orally and in writing in the first part of the information and consent form (appendices 1 and 2). Each page of the form will be initialled at the bottom of each page so that the person who is a subject in this study certifies that he or she has in fact received this information. Information is written in clear, perfectly comprehensible language for the person. It must contain all of the elements which the person must be informed of, in compliance with the Public Health Code, article L 1122-1.

Consent to participate is collected from each person enrolled in the study in writing in the second part of the information and consent form. This second party must be written in clear language, perfectly comprehensible for the person who is a subject in this study. It must contain all items which the person consents to. Consent is given by the subject by signing this consent form by the person who is the subject in the study with their first name, surname and date of consent.

Furthermore, the investigator who collects the consent, dates and signs the form in the place provided for this purpose and ensures 1) that the form complies and that no item or date is missing, 2) that an original of the document is given to the patient. He/she keeps a second original copy in a secure place and access to which is controlled and 3) places a copy of his/her original in a secure envelope notified to the attention of the sponsor; this third copy must not be opened by the sponsor, but kept with the study permanent dossier.

The investigator must make certain that the person who is a subject in this study has the time to freely decide and will have read and understood the information and consent form.

The information and consent form is a document which will have been approved by the CPP prior to the start of the study, during examination of the protocol.

## Compensation

A fee of 500 Euros will be paid to the subject at the end of the study, i.e. after 24 weeks of participation. If a subject decides to discontinue the study before its completion, no compensation will be paid on a prorate basis.

# Objectives

## Primary objective

To evaluate the influence of 4 month supplementation with Glycabiane on fasting glycaemia in overweight or obese prediabetic subjects.

## Secondary objectives

To study the influence of 4 month supplementation with Glycabiane on:

- glycosylated haemoglobin (HbA1c)
- lipid profile (Total cholesterol, LDL-c, HDL-c, TG)
- markers of inflammation: circulating levels of IL6 and of CRPus
- insulin resistance (HOMA, QUICKI revised)

HOMA IR: I_0_/(22.5Xe^-ln(gly^_0_))

QUICKI revised: 1/[Log(I_0_)+ Log(gly_0_) + Log(FFA_0_)]

● markers of adiposity: weight, BMI (weight/height²), waist circumference, hip circumference, body composition (including percentage of fatty mass and lean mass), adipocyte size

- inflammatory status of adipose tissue (secretion of IL-6 and adiponectin)
- sensitivity to insulin of adipose by measurement of adiponectin secreted and by study of Akt phosphorylation

To evaluate safety of 4 month supplementation with Glycabiane

To evaluate dietary intake before and after supplementation

# Experimental design

## Study design

This study is a single centre, national, randomised, double-blind, placebo-controlled study with five parallel groups in a clinical trial of efficacy.

It is a longitudinal study with planned follow-up of 5 visits: D-14 to -1 D0, D60±7, D120±7 and D180±7.

## Research category:

A biomedical research study not concerning a product mentioned in article L. 5311-1 of the public health code.

A biomedical research study concerning a dietary supplement.

## Statement of the experimental design

A population of 60 overweight or obese prediabetic subjects will be selected after an examination for pre-inclusion.

After inclusion, subjects will be randomised to 2 groups to follow 4 month supplementation either with Glycabiane or with Placebo (2 capsules per day). Three follow-up visits will also be scheduled: one after 2 months of supplementation, another at the end of the 4 month supplementation period, and a last one 2 months after the end of supplementation at 6 months.

*Overall study flowchart*

| Visits | V1  Inclusion | V2  D0-Tr | V3  2M | V4  4M  End of tr. | V5  6M  follow-up |
| --- | --- | --- | --- | --- | --- |
| Days | -14 to -1 | 0 | 60±7 | 120±7 | 180±7 |
| Criteria for inclusion/non-inclusion | X | X |  |  |  |
| Invest. Treatment |  | X | x | x |  |
| Information/  Signature of consent form | X | ~~X~~ |  |  |  |
| BP | X | X | X | X | X |
| Weight (kg) | X | X | x | X | x |
| Waistline | X | X | x | x | x |
| **Somatic and nutritional evaluation**  **-** Anthropometric measurements  - Measurements of body composition (DEXA)  - Nutritional diary (see appendix 7) | X  X | x  x  x | x  x | x  x  x | x  x |
| **Screening evaluation:**  -Liver function test evaluations:  AST, ALT, gamma GT  - Renal evaluation: Expanded Ionno  - CBC + platelets  - Serology: HIV, Hepatitis C  - Pregnancy test: Beta-hCG | X |  |  |  |  |
| **Metabolic evaluation**  - Glycaemia  - Serum insulin,  - Lipid evaluation (Cholesterol, LDL-, HDL- Cholesterol, TG)  - Free fatty acids (FFA)  - % HbA1c | x  X | x  x  x  X  x |  | x  x  x  X  x | x  x  x  X  x |
| **Inflammatory evaluation**  - CRPus, IL6 |  | X |  | x | x |
| **Adipose signals**  - Leptine, adiponectin  - Needle puncture of subcutaneous abdominal adipose tissue  - Adipocyte size:  - Study of adipocytes in culture (in vitro) |  | X  x |  | X  x | X |
| **Quality of life questionnaires (TFEQ, physical activity)**  **Dietary questionnaire (diary)** | X | X  X | X | X  X | X  X |

# Evaluation endpoints

## Primary endpoint

Quantification of improvement in fasting glycaemia.

## Secondary endpoints:

Evaluation of changes in percentage of HbA1c, lipid profile, serum insulin, serum leptin, anthropometric characteristics, adipocyte size at end of supplementation, inflammatory status and sensitivity of adipose tissue to insulin.

Quality of life evaluation

# Screening of persons

## Participating studied population:

### Duration of participation of persons in the study:

Total duration of participation of each subject will be 6 consecutive months.

### Description of participating persons:

Criteria for inclusion

- Men and women 25 to 65 years of age,

- Who satisfy the following criteria:

- 1.0 g/L ≤ fasting glycaemia < 1.26 g/L

- Overweight or obese (BMI ≥ 25)

- Who do not wish to change their dietary habits and physical activity,

- With normal renal and liver function test evaluations,

- Covered by the French social security system,

- Sufficiently cooperative to comply with requirements of the study,

- Immunocompetent status (no known HIV nor hepatitis C virus),

- Use of an effective method of contraception for women of childbearing potential

Criteria for exclusion

- thyroid disease
- hypogonadism

- history of a musculo-skeletal, auto-immune or neurological disorder

- hepatitis C, HIV

- medical therapy for thyroid disease, hypoglycaemia or anti-coagulant therapy

- medical therapy than can have an influence on:

- weight gain/loss

- glycaemic control

- serum insulin

- pregnant women or nursing mother

- weight loss > 5% during last 6 months

- ingestion of a dietary supplement containing cinnamon or chromium

- subject who is under legal protection,
- subject who collected €4,500 as compensation for participating in clinical trials during the last 12 months. This information is to be verified by the investigator, subjects are compensated in research studies
- history of allergy to one of the components of the investigational product
- contra-indication to use of xylocaine as a local anaesthetic

### Number of persons to include:

Calculation of sample size: 25 subjects per group are necessary to demonstrate a change in fasting glycaemia of at least 10% and of a standard deviation 0.13 g/L (mean of standard deviations observed in clinical studies published on type 2 diabetes and cinnamon), setting an α risk of 0.05 and a β risk of 0.10. 60 subjects will be selected in the study according to criteria for inclusion and non-inclusion to ensure inclusion and follow-up of the study for 50 subjects in its totality.

## Recruitment and withdrawals from study

### Recruitment

Subjects will be recruited by different methods of a communication campaign displayed:

- in medical offices of doctors practicing in Paris or in the Paris area, via posters printed by the CRNH (see appendix 8)
- doctors who wish to do so may also offer the study to patients during a consultation. Their contribution to the study will be limited to pre-screening of overweight and or obese prediabetic patients based on voluntary participation of subjects with an abnormal glycaemia (1.0 g/L ≥ fasting glycaemia > 1.26 g/L), satisfying screening criteria, who read the information sheet and accept to come to the department of Nutrition of Pitié Salpêtrière hospital group to validate their inclusion in this study or not.
- in biomedical clinical analyses laboratories, pharmacies, centres of screening for diabetes via posters printed by the CRNH (see appendix 8)
- in the daily newspaper “Métro”, Paris edition (see copy of poster in appendix 8)
- via “Volterys”, a “meeting site” for volunteer subjects and researchers

Subjects will be selected based on medical interview, a complete clinical examination performed by the investigator or co-investigator and laboratory test evaluation.

### Withdrawals from study

**Criteria for discontinuation or withdrawal from study:**

- a serious adverse event, that is “any event or adverse effect which results in death, is life-threatening for the person who is the subject in the research, requires hospitalisation or results in prolongation of hospitalisation, causes incapacity or major or durable disability or in fact is translated by a congenital anomaly or malformation, whatever the dose administered” (ICH E6: GCP, 1.50).
- non-compliance with the study protocol.

**Deliberate decision of a person:**

Withdrawal from the study is possible at any time if requested by the participant. In particular, if participants do not wish to continue tests, it is sufficient for them to notify this to the examiner.

**An imposed decision**:

Participants who cannot execute tasks relating to the study will be excluded from the study.

### Exclusion period and participation in another study

The exclusion period defined in the setting of this study is 3 months, period during which the subject cannot participate in another clinical research study.

This exclusion is justified to prevent any risk of interference in interpretation of results.

The investigator makes certain that persons who may be subjects in the biomedical research study are not already participating in another study which may make their inclusion in the proposed study impossible. He/she also makes certain that they have not participated in a study for which currently an exclusion period is required, in particular, using the National File of persons who are subjects in biomedical research studies.

# Practical conduct of study

## Detailed description of procedures practiced on persons:

So-called screening/inclusion visit D-14 to D-1

- Information of the person who is a subject in the study and collection of his or her consent
- Medical visit and nutritional evaluation
- Laboratory test evaluation specific for inclusion consisting of:
  - complete blood count with white blood cell differential count (CBC-) and erythrocyte sedimentation rate (ESR),
  - HIV and HCV serologies,
  - renal function tests (serum electrolytes (plasma sodium, plasma potassium, calcium, alkaline reserve, serum phosphorus), creatinine, blood urea nitrogen)
  - liver function tests (ALT, AST, Gamma GT)
  - lipid parameter profile (Total cholesterol, HDL and LDL cholesterol, triglycerides)
  - glycaemia
  - a pregnancy test if a female volunteer is not post-menopausal and not using an effective method of contraception
  - nutritional diary (see appendix 7)

Visit V2-D0 -randomisation:

- Verification of criteria for inclusion
- Clinical examination, somatic and nutritional evaluation (blood pressure, weight, waistline, anthropometric measurements including body composition, nutritional diary)
- Laboratory test evaluation after an overnight fast of 8 hours, and including measurement of glycaemia, serum insulin, total cholesterol, LDL-, HDL-, triglycerides, CRPus, IL6, serum leptin, HbA1c
- Needle aspiration of subcutaneous adipose tissue
- Distribution of a nutritional diary and of an operational diary for the next visit
- Distribution and retrieval of quality of life questionnaires TFEQ*, physical activity questionnaires (see appendix 6) and nutritional diary (appendix 7)
- Distribution of treatment (Glycabiane)
- Measurement of body composition (DEXA)
- Setting of an appointment for visit 3 (D60±7)

*The Three Factor Eating Questionnaire (TFEQ) is a self-questionnaire which makes it possible to study three dimensions of dietary behaviour: hunger, restriction and disinhibition. It is defined by occurrence of uncontrolled access of hyperphagia, which most often are subsequent to lifting of dietary restrictions.

Visit 3 (V3 at month 2) at D 60 ± 7

- Clinical examination, somatic and nutritional evaluation (blood pressure, weight, waistline, anthropometric measurements, nutritional diary)
- Recovery of empty boxes and blisters of products consumed
- Setting of an appointment for end of supplementation visit (D120±7)

Visit 4 (V4 at month 4) at end of supplementation at D 120 ± 7

- Recovery of empty boxes and blisters of products consumed and of remaining products not consumed
- Distribution of nutritional diary and of operating diary for next visit
- Distribution and recovery of quality of life questionnaires, physical activity questionnaire and nutritional diary (appendix 7)
- Clinical examination, somatic and nutritional evaluation (blood pressure, weight, waistline, anthropometric measurements, nutritional diary)
- Laboratory test evaluation after an overnight fast of 8 hours, and including glycaemia, serum insulin, total cholesterol, LDL-, HDL-, triglycerides, CRPus, IL6, serum leptin, HbA1c
- Needle aspiration of subcutaneous adipose tissue
- Measurement of body composition (DEXA)
- Setting of an appointment for the follow-up visit (D194± 7)

Visit 5 (V5-at month 6) of follow-up at D 194 ± 7

- Clinical examination, somatic and nutritional evaluation (blood pressure, weight, waistline, anthropometric measurements, nutritional diary)
- Laboratory test evaluation after an overnight fast of 8 hours, and including glycaemia, serum insulin, total cholesterol, LDL-, HDL-, triglycerides, CRPus, IL6, serum leptin, HbA1c
- Collection of nutritional diary and of operating diary
- Distribution and recovery of quality of life questionnaires
- End of study

**Nutritional and inflammatory evaluation**

We are going to characterise the lipid profile and glycaemic control of subject: before and after each period. At start and at end of each period, after overnight fasting for 8 hours and blood sample will be collected from the subject fasting (for measurement of glycaemia, serum insulin, total cholesterol, LDL, HDL, triglycerides, leptin, and adiponectin). Inflammatory factors: CRPus and IL6 will be measured (ELISA).

Fatty mass and its distribution will be measured with dual energy X-ray absorptiometry (DEXA). A sample of abdominal subcutaneous adipose tissue collected by needle puncture will be performed by suctioning under a vacuum after local anaesthesia with 10% xylocaine without isoprenaline.

We are going to characterise the dietary profile throughout the study using the nutritional diary, of a “weekly” type over 3 days.

**Sampling of adipose tissue:**

Samples of adipose tissue under the abdominal skin (about 500 mg) will be collected by needle puncture at time of inclusion and after follow-up. This sample, collected by the investigator, is performed in routine practice in the department of nutrition of Pitié Salpetrière Hospital. After local anaesthesia (1% Xylocaine without adrenaline), suctioning of adipose tissue with a very small calibre needle is performed. Part of the sample will make it possible to measure adipocyte size before and after supplementation. Another will be used to evaluate:

1) the inflammatory status of adipose tissue with a ELISA assay measurement of cytokine IL-6 and of adipokine, adiponectin.

2) sensitivity to insulin by study of phosphorylation Akt by Western Blot technique and by secretion of adiponectin.

These analyses 1) and 2) will be subcontracted to AdipoPhYt firm.

**Processing of biological samples**

All sera intended for measurements of signs of adiposity and inflammatory factors will be analysed in the INSERM Unit U872 (Eq 7). The serum collection will be stored by the Inserm Unit U872 (Eq. 7). For each sample of adipose tissue (subcutaneous) the cell size will be evaluated before and after treatment. Investigational studies of adipose in vitro in culture (inflammatory status and sensitivity to insulin) will be performed by AdipoPhYt.

## Description of general logistical organisation of study

Investigators in the INSERM laboratory U872 (Eq 7), Endocrinology unit, CRNH-Metropolitan Paris in Pitié Salpêtrière will be responsible for recruiting (subcontracted to AdipoPhYt) and selection of patients, dispensing of products, measurement and recording in the case report forms of evaluation criteria.

PiLeJe will provide the product (placebo and active product). A clinical research associate (CRA) from the sponsor will perform monitoring of the study to verify source data and conduct of the study according to good clinical practice. Staff from AdipoPhYt will contact the subject the night before the procedure to remind him or her of the appointment and the requirement to maintain fasting for 8 hours.

INSERM laboratory U872 (Eq 7) is in charge of writing statistical chapters of the protocol, data management, statistical analysis and of writing of the statistical report.

Products and placebo will be blinded by the sponsor (Laboratoire Pileje)

## Provisional study schedule (Flow-Chart)

- Duration of inclusion period: 6 months

In case of withdrawals from study for medical intercurrent reason, subject withdrawn will not replaced

- Total study duration: 24 months
- Total duration of participation of each subject will be 194 consecutive days.

# Studied product

## Description of the studied product

### Glycabiane

- - Medicinal product: a dietary supplement
  - Active substances: OPC-A cinnamon, guanylor chromium and carnosine
  - Pharmaceutical formulation: capsules
  - Composition: one capsule of 595 mg of Glycabiane contains:

| **components** | **Quantity/capsule** |
| --- | --- |
| Cinnamon extract | 228.000 mg |
| Calcium carbonate | 136.750 mg |
| L-carnosine | 100.000 mg |
| Transparent HPMC capsules (size 0) | 95.000 mg |
| Silica (SIPERNAT) | 16.000 mg |
| Talc | 7.000 mg |
| Magnesium stearate | 6.000 mg |
| Silica hydrate | 5.000 mg |
| Guanylor chromium | 1.250 mg |
|  |  |
| **TOTAL** | 595 mg |

In blue, active substances

- Method of administration: oral route
- Dosage: 2 capsules per day to be taken during the noon time meal during 4 months
- Packaging: blisters of 20 capsules
- Labelling: in compliance with decision of 24 May 2006 (Official Journal of 30 May 2006 – text no. 41) setting content of labelling of investigational medicinal products.

### Placebo

- - Medicinal product: placebo
  - Pharmaceutical formulation: capsules
  - Composition: one capsule of 496 mg of Placebo contains:

| Components | Quantity/capsule |
| --- | --- |
| Calcium carbonate | 136.750 mg |
| Transparent HPMC capsule (size 0) | 95.000 mg |
| Talc | 7.000 mg |
| Magnesium stearate | 6.000 mg |
| Silica hydrate | 5.000 mg |
| Silicium dioxide | 16.000 mg |
| Microcrystalline cellulose | 230.25 mg |
|  |  |
| TOTAL | **496 mg** |

- Method of administration: oral route
- Dosage: 2 capsules per day to be taken during the noon time meal during 4 months
- Packaging: blisters of 20 capsules
- Labelling: in compliance with decision of 24 May 2006 (Official Journal of 30 May 2006 – text no. 41) setting content of labelling of investigational medicinal products.

## Dispensing and compliance

The studied product and placebo will be delivered directly to the INSERM laboratory U872 (Eq7).

Temperature for storage of products must be below 25 °C.

Unused products will be recovered and collected by the sponsor who agrees to eliminate waste products in compliance with regulations in force.

Documents describing handling for elimination of waste products (forms for monitoring of waste products, vouchers for collection, etc.) will be issued by the sponsor.

Compliance will be evaluated by means of 2 parameters:

- the patient “interview”;
- the return of unused products;

No medicinal product or treatment that may have an action on glycaemic control in persons who are subjects in the biomedical research will be authorised during the trial.

# Medical devices

- Measurement of body composition

Body weight will be measured with a scale with the patient in underwear to an accuracy of ± 0.1 kg. Height will be measured with the patient barefoot to an accuracy of ± 0.1 cm. Circumferences will be measured with a seamstress’ tape measure. Waistline is the smallest circumference at the height of the umbilicus and hip circumference is the largest circumference opposite the femoral heads. These methods are standardised in the CRNH and are performed by the same investigator.

Body composition will be measured by dual energy X-ray absorptiometry (DEXA). This method is usually used for measurement of bone mineral density. It is based on body scanning with an X-ray beam at 2 different levels, which make it possible to individualise 3 compartments: mineral mass, active tissue mass and fatty mass. This method that uses little radiation (less than that of one conventional chest X-ray) is used in adults, as well as in children and now is considered as the reference method to measure body compartments precisely. For this examination, the subject is supine on a table, in his or her underwear. The total duration of the study is about 10 minutes.

- Fasting blood sample collection

In the morning, fasting (an 8 hour fast), volunteers will undergo blood sample collection of about 40 ml which immediately will be centrifuged, divided into aliquots and frozen for analysis of substrates and hormones. We will measure the circulating levels of the following:

- glucose and insulin
- lipids: cholesterol, triglycerides, free fatty acids (FFA) and high and low density lipoproteins (HDL and LDL- cholesterol)
- leptin and adiponectin
- inflammatory factors: CRPus and IL6.

About 7 ml of blood will be collected to obtain plasma or serum which will be stored in order to perform additional assay measurements regarding results of previous measurements.

All sera intended for assay measurement of signals of adiposity and of inflammatory factors will be analysed in the INSERM Unit U872 (Eq 7). The serum collection will be stored by the Inserm Unit U872 (Eq. 7). Total blood volume collected in four times of visits therefore will be about 160 mL for the totality of the study. Blood samples will be stored at -80 °C up until the end of the study, and then will be destroyed.

- Sampling of peri-umbilical adipose tissue

A sample of adipose tissue of about 500 mg will be collected by needle puncture suctioning performed on volunteers. This sample, collected by the investigator, is performed in routine practice in the department of nutrition of Pitié Salpetrière Hospital to measure cell size. After local anaesthesia with 2 ml of 1% xylocaine without isoprenaline, the peri-umbilical adipose tissue will be collected by a small calibre needle with suctioning to follow the outcome of the population and morphology. For each adipose tissue sample collected (subcutaneous), cell size will be evaluated before and after treatment.

Investigational studies for investigation of adipose tissue (inflammatory status, insulin sensitivity) in vitro in culture will be performed by the AdipoPhyt firm.

- Specific instructions during the investigational period

Persons who are subjects in the study must not change their dietary habits. Lastly, any form of medical therapy initiated during the investigational period must be reported by the volunteer.

# Collection of human biological samples

- Description of type of samples:

Blood samples and adipose tissue.

- Description of conditions for sampling:

Blood samples will be collected at D-1 to -14, D0, D 120 and D180 after 8 hours of fasting, between 8 and 9:30 am. Test tubes collected will be kept at 4 °C starting with collection of the sample, centrifuged to 4 °C, divided into aliquots and stored at -80 °C until their use for the planned analyses.

The sample of adipose tissue will be collected after application of local anaesthesia with 2 ml of 1% xylocaine without isoprenaline, peri-umbilical adipose tissue sample will be collected by suctioning with a fine needle to measure the adipocyte size immediately and to conduct study of cell culture in vitro.

Blood samples will be stored by INSERM U872 (Eq7) at -80 °C up until end of study (last analysis of last patient included), and then they will be destroyed. Those of peri-umbilical adipose tissue will be stored at -80 °C by ADIPOPHYT up until end of study (last analysis of last patient included), and then they will be destroyed.

# study safety

## DEFINITIONS RELATING TO SAFETY

See article R. 1123-39 no. 1; no. 2; no. 6; and no. 8 of PHC

Adverse event

Any harmful event occurring in a person who is a subject in a biomedical research study whether this occurrence is related to the study or not or to a product on which the study is focused.

Adverse effect

Any adverse event related to the study.

For each adverse event, the investigator and the sponsor evaluate its causal relation with the study. Any adverse event considered by the investigator or by the sponsor as having a scientifically reasonable causal relation with the study is qualified as an adverse effect. The expression “scientifically reasonable causal relation”, generally means that evidence or an argument exists enabling to suggest, scientifically, a cause-to-effect relationship between the harmful and unwanted reaction and the study (i.e. procedure, method, action or product which is the subject of the study). An adverse event due to the study can be related, for example, to the procedures, methods, to the procedures performed or to the products which are the subject of the study or used for requirements of the study.

Serious adverse event or effect

Any adverse event or effect which meets the definition of “serious” if:

- it results in death;

- is life-threatening for the person who is the subject of the study;

- requires hospitalisation or results in prolongation of hospitalisation;

- causes important or durable incapacity or disability;

- is translated by a congenital anomaly or malformation.

For each event/ adverse effect, the investigator and sponsor evaluate its causal relationship.

The term “life-threatening” corresponds to an event/effect which is immediately life-threatening for the person who is a subject in the study at time of the adverse event and not an event/effect which could possibly have caused death if it had been more severe. Medical judgement must be exercised to determine if the adverse event or effect is serious in other cases. Major adverse effects or events which are not immediately life-threatening or which do not result in death or in hospitalisation, but which can place a subject in danger or require an intervention to prevent its progression to one of the aforementioned criterion must also be considered as serious.

Unexpected serious adverse event

An unexpected adverse event means any adverse event whose nature, severity or outcome do not coincide with information relating to products, procedures performed and methods used during the study.

For each adverse event, the sponsor must evaluate if it is unexpected or not.

Evaluation of the unexpected feature of an adverse event is done based on information described in the study protocol or in the investigator brochure, in particular, if applicable, relating to procedures and method performed during the study or to products which are subject of the study or used for requirements of the study.

## investigators’ role

### Evaluation of severity/causal relation of adverse events

The investigator must evaluate the following for each adverse event collected during the study:

- its seriousness,

- its causal relation between the serious adverse event and the study, and then send the result of his or her evaluation to the sponsor.

Notification of serious adverse events to the sponsor

The investigator will notify without delay starting from day when he/she became aware of it all SAE which occurred during the study:

Using the SAE reporting form that he/she will send by fax and by e-mail to the sponsor at +33 (0)1.45.51.12.74 . Att. Mrs. Séverine BIEUVELET

In this notification, the investigator sends the sponsor his/her evaluation of the causal relation of the serious adverse event with the study (that is, with the procedure, method, procedure or product that is the subject of the study).

The investigator will also provide all information necessary for medical evaluation of the observation to the sponsor (laboratory test reports, pathology report, and radiology report).

The sponsor can request further information from the investigator.

All documents sent by the investigator must be made anonymous.

SAE occurring after the study must be notified to the sponsor and followed up until their final resolution.

**Notification of adverse events to the sponsor**

The investigator will complete an adverse event form for all adverse events that occurred during the study. Such events will be recorded in a dedicated Excel spreadsheet at the study site. The CRA will retrieve pages as monitoring visits are completed and will send a copy of them to the safety unit upon his/her return from the monitoring visit. A copy of the updated Excel spreadsheet will also be sent to the Sponsor. If analysis of cases results in an SAE, he/she must follow the procedure for notification and follow-up of SAEs described in the aforementioned..

## sponsor’s role

The sponsor must report all suspect serious adverse events related to the product or to the study to the CPP Ile-de-France VI and to AFSSAPS without delay and at latest with an initial time of 7 calendar days starting from when he/she became aware of it for serious adverse events which resulted in death or where life-threatening and within 15 days for other serious adverse events. Any initial report will be completed by a follow-up report delaying relevant further information concerning serious adverse events within a time period of 8 days starting from the abovementioned deadlines.

The sponsor will make a decision based on significance of the serious adverse events that he/she reports and the consequences that he/she draws, in particular, concerning conduct of the study.

The sponsor will also make a decision on the causal relation of the adverse event using a joint analysis with the team from PiLeJe safety staff. The unexpected feature will be established by the sponsor based on nutritional safety data from Pileje concerning the studied product and also the scientific and medical literature concerning the component ingredients of the product.

The sponsor will keep up-to-date detailed registries of all adverse events (AE, SAE, USAE)

The sponsor will periodically report to AFSSAPS, as well as to the CPP:

- Each semester:
- a list of suspect unexpected SAE that occurred in other trials conducted in France with the same product, procedure or method
- a concise synthesis underlining the principle problems of safety raised.
- Yearly:
- an annual safety report taking into account all available safety information.

The sponsor keeps up-to-date detailed registries of all adverse events notified to his/her by investigators.

## follow-up of patients in case of a serious adverse event

Follow-up of patients who have had a serious adverse event will be performed by the doctor responsible for the study.

## Independent monitoring committee

There is no monitoring committee because this is not necessary considering the type of product under study: the risk is almost non-existent because it is a combination of known dietary ingredients already used in other formulations of dietary supplements marketed by the Pileje group (OligobianeCr (launched on the market in early 2001) and Multibiane Age Protect (launched on the market in March 2008) for Guanylor®-Cr and Glycabiane (launched on the market in March 2008) for the combination Glycabiane and nutrivigilance safety is free of any adverse event.

# Data collection and processing

## data collection

Data in the form of questionnaires will be collected in case report forms by investigators mandated in this study. Data concerning biological samples from patients will be inserted in the medical dossier of the person who is a subject in the study. An anonymised copy will be inserted in the CRF in a plastic folder provided for this purpose.

## Description of channel of data management before data processing

Data are stored in locking cabinets on-site.

## Data collection

Data will be collected in compliance with confidentiality of the identity of persons participating in this study and all personal or medical information concerning them. Patients’ anonymity will be ensured by inserting at most the first 3 letters of the patient’s surname and the first 2 letters of his first name on all documents necessary for this study or by erasure by appropriate means (white correction marker, correcting fluid, etc.) of data associated with a name in copies of source documents, intended for documentation of the study.

Clinical data will be reported or results will be stapled in the case report forms using data from the database after they are made anonymous.

Data collection will be performed by means of case report forms specific for the study. Each dossier will be completed progressively as information is collected. If data are missing, the latter will be indicated by the sign “MD” (missing data). If there are data not applicable to the subject included in the study, such data will be annotated with the term “NA” (not applicable). If corrections must be made to the case report form, the erroneous data must be crossed out with a single line (without concealing the original entry), the new data must be written next to it with the investigator’s initials and date on which the correction was made. Investigators will sign pages of the CRF corresponding to their examination.

Subjects will have the right of access to source data and documents through the study investigators. Data will be archived confidentially for a period of 15 years after the end of the study. Healthy volunteers, in compliance with legislation, will also be informed of the overall results of the study.

Data will be stored in locking cabinets on-site.

## Computerised data processing

Data are digitised in single entry with double reading. This will be done under the responsibility of Dr. Salwa Rizkalla, co-investigator of the study, who validates data after each entry. Data is entered in an Excel spreadsheet where each page corresponds to a type of parameter. A summary page makes it possible to record all data on a single page, which enables it to be transferred to the statistical utilisation software (SAS).

- Traceability will be ensured by an attached table. Each change will be noted, dated, and listing the reason and the author of this change.

− Data will be kept in a computer located on Dr. S Rizkalla’s desk; this computer is on the secure network of the Pité Salpêtrière hospital, a hospital of the APHP[[4]](#footnote-5), protected with a specific ID and password.

− Weekly saving on to a computer data stick dedicated to the study, as well as saving of “paper” documents will also be performed in the laboratory. All these data will be stored in a locking file cabinet.

− Similarly, procedures, names, addresses, phone and fax numbers and e-mails of doctors participating in this study and reports of meetings of the steering committee and of follow-up of study will be kept in a room and a locking cabinet. Access will be controlled and restricted to a list of participants established by the scientific person responsible.

- Lastly, all these data will be transferred to the server for saving in Unit U872 (team 7) which is protected according to rules in force in INSERM.

Confidentiality relating to each volunteer participating in this study is assured. Clinical data and source data will be accessed directly in case of an audit ordered by the sponsor or an inspection conducted by the competent administrative authorities.

# Statistical analysis of data

## Number of subjects necessary

Based on the literature, a 10% decrease in fasting glycaemia can be expected in the Glycabiane group compared to the Control group, after 4 months of supplementation. The statistical hypothesis is a 10% difference in the primary evaluation end point (Fasting glycaemia) between subjects who received the placebo and those who received Glycabiane®. Therefore, the number of subjects to be enrolled in this trial, with an alpha risk of 5% and a power of 90%, is 25 per group, i.e. a total of 50 subjects. 60 subjects will be screened in the study to ensure inclusion and follow-up of a total of 50 patients.

**Statistical considerations**

All data will be listed by group and by subject.

Descriptive statistics will be compiled by type of parameter analysed:

- Quantitative variable: sample size, mean, standard deviation, minimum and maximum of raw data and, if necessary, changes compared to baseline, as well as percent change.
- Qualitative variable: sample size, absolute frequency (N) and relative frequency (%) by class.

Descriptive statistics will be compiled by group.

Graphs will be provided according to type of variable:

Quantitative variable: Representation by group of the mean +/- SEM during visits.

Qualitative variable: Representation by group of percentages by class

Changes to clinical and biological parameters will be analysed for each subject before and during kinetic follow-up. Manova and Anova kinetic tests will be used. We will relate clinical, biological parameters, morphological data and data expression by logistic regression analysis. INSERM U872 (Eq7) over the last few years has gained expertise in analysis of these data (use of JMP software (derived from SAS software). For quantitative data, repeated measures analysis of variance will be used (Anova, Manova, logistic regression model). Statistical tests will be performed with an alpha risk of concluding in an erroneous difference set at 5%.

Statistical analysis will be performed by INSERM U872 (Eq7).

Description of sample

A description of subjects not included, subjects included who completed the study and of subjects included who withdrew from the study before its completion will be presented with a table of absolute frequency (N) and relative frequency (%).

Reasons for withdrawals will be presented in a listing.

A description of subjects at baseline, that is, before use of the studied product, will be reported by descriptive statistics on available demographic data (age, sex, height, weight and BMI).

**Primary analysis**

The primary evaluation end point will be the fasting glycaemia before and after supplementation. It will be compared within groups and between the two groups using a test of comparison of means.

**Secondary analyses**

Other comparisons (secondary evaluation end points) will be:

- levels of HbA1c before and after supplementation. Comparison will be performed within groups and between the two groups.

- concentrations of total cholesterol, HDL-c, LDL-c, TG, ND leptin before and after supplementation. Comparison will be performed within groups and between the two groups

- serum insulin and HOMA before and after supplementation, within groups and between the two groups comparison

- quality of life before and after supplementation

- systemic inflammatory status (IL-6, CRPus)

- adipocyte size before and after supplementation

- inflammatory status of adipose tissue in vitro by ELISA assay measurement (IL-6, adiponectin)

- sensitivity to insulin of adipose tissue in vitro by study of adiponectin secretion and of Akt phosphorylation

# Cnil[[5]](#footnote-6)

This study falls within the scope of the “Methodology of Reference” (MR-001) in application of conditions of law of 6 August 2004 relating to protection of physical persons regarding processing of personal data and modifying law of 6 January 1978 relating to data processing, computer files and freedoms. This change has been certified by decision of 5 January 2006.

# Archiving

PiLeJe, in its capacity as the sponsor, saves documents and data relating to the trial in compliance with legislative and regulatory conditions in force, in particular, the decision of 8 November 2006 setting the duration of storage by the sponsor and the investigator of documents and data relating to a biomedical research study involving a medicinal product for human use.

This duration of storage of documents is set at 15 years.

Documents concerned are the following, in particular:

- the investigator brochure;
- the study protocol and any possible amendments signed, specimens of case report forms and operating diaries and all documents/media used to collect data;
- information communicated to the person who is the subject in the study and forms for collection of his or her consent;
- financial aspects of the study;
- insurance policy covering the sponsor’s civil responsibility and an insurance certificate delivered by the insurance company;
- contracts or agreements signed between the different persons (sponsor, CRO, investigators, etc.);
- dossier requesting an opinion from the CPP, exchanges of correspondence and opinions from the CPP;
- names of persons in the CPP who took part in the deliberations on the research project;
- *Curriculum vitae* of investigators and of collaborators involved in the study, list of the investigators’ assistants ;
- Completed case report forms, dated and signed;
- AE, SAE, USAE forms.

PiLeJe, in its capacity as the sponsor, informs the investigators in writing of the need to save documents and data relating to the study up until it is notified to them that such documents no longer are necessary.

# Communication

Data will be disclosed only after the prior joint agreement of the investigator and of the sponsor. Results will be the subject of communications and published articles.

Furthermore, at the end of the study, persons who were subjects in the study have the right to be informed of overall results of the study, according to modalities which will be specified in the information document (article L. 1121-1 of the Public Health Code).

# Substantial changes to the protocol

Changes made to the protocol must be qualified as substantial or not.

Depending on their type, they will be subject to another opinion from the Committee for the Protection of Persons and AFSSAPS.

Any substantial change to the protocol concerning the study objectives, its design, population, tests or significant administrative aspects will require approval by the investigator, the sponsor, and a favourable opinion of the CPP. Another consent form for persons participating in this study will be collected, if necessary.

# Confidentiality

## Access to data

The sponsor is in charge of obtaining consent from all parties involved in the study in order to ensure direct access to all of the places where the study is conducted, to source data and reports for the purpose of quality control and an audit by the sponsor.

Investigators will make available individual documents and data strictly necessary for follow-up, quality control and an audit of the biomedical research study will be made available to persons with access to documents in compliance with legislative and regulatory conditions in force (articles L.1121-3 and R.5121-13 of the Public Health Code).

## Source data

Since source documents are defined as any original document or object making it possible to prove the existence or accuracy of data or a fact recorded during the clinical study will be kept during 15 years by the investigator or by the hospital if it involves a hospital medical dossier. In this study, only the medical dossier is considered as source data.

## Confidentiality of data

In compliance with conditions concerning confidentiality of data to which persons in charge of quality control of a biomedical research study have access (article L.1121-3 of the Public Health Code), in compliance with conditions relating to confidentiality of information, in particular, concerning the nature of the investigational products, tests, persons who are subjects in this study and results obtained (article R. 5121-13 of the Public Health Code), persons with direct access will take all necessary precautions to ensure the confidentiality of information relating to investigational medicinal products, tests, persons who are subjects and, in particular, concerning their identity, as well as the results obtained.

These persons, in the same capacity as the investigators, are subject to professional secrecy (according to conditions defined by articles 226-13 and 226-14 of the Penal Code).

During the biomedical research study or at its end, data collected on persons who are subjects and sent to the sponsor by the investigators (or all other specialised participants) will be made anonymous.

The names of persons concerned, nor their address, in any case must not be clearly legible.

Only the first three letters of a subject’s surname and the first two letters of their first name will be recorded, together with a code number specific for the study indicating the order of inclusion of subjects.

The sponsor will ensure that each person who is a subject in the study has given his or her agreement in writing for access to individual, personal data strictly necessary for quality control of the study.

## Registration in the national file of persons who are subjects in biomedical research

In application of article L. 1121-16 of the public health code, the names of persons who are subjects in biomedical research will be recorded in a national file of persons who are subjects in biomedical research: VRB (Volunteer Subjects for Biomedical Research)

The investigators will be the only persons authorised to enter the names of volunteers in this file.

# Quality assurance

## Commitment by investigators and sponsor

The investigator agrees that this study will be conducted in compliance with public health law no. 2004-806 of 9 August 2004 concerning biomedical research, application decision no. 2006-477 of 26/04/2006 modifying chapter I title II of book 1 of the 1^st^ part of the Public Health Code relating biomedical research, as well as the decisions in force.

Rules on good clinical practice (GCP) for biomedical research on medicinal products for human use, mentioned in article L. 1121-3 of the Public Health Code and decision of 24 November 2006 will also be applied (see Appendix 10).

The investigator also agrees to work in agreement with the Declaration of Helsinki of the World Medical Association (Tokyo 2004, revised version). (See Appendix 9).

## Quality Assurance

A Clinical Research Associate (CRA) mandated by the sponsor will ensure proper conduct of the study, the collection of data generated in writing, their documentation, recording and report, in agreement with Procedures and in compliance with Good Clinical Practice, as well as legislative and regulatory conditions in force.

## Quality control

The investigator is the guarantor of the authenticity of data collected in the setting of the study and accepts legal conditions authorising the sponsor of the study to set up quality control.

The investigator and co-investigators therefore accept to make themselves available at visits for Quality Control performed at regular intervals by the Clinical Research Associate. At these visits, the following items will be reviewed:

- informed consent and information forms;
- compliance with study protocol and procedures defined in them;
- quality of data collected in the case report forms: accuracy, missing data, consistency of data with “source” documents (medical dossiers, log books of appointments, originals of laboratory test results, etc.);
- management of products.

## Case report forms

All information required by the study protocol must be entered into case report forms and an explanation must be provided for all missing data. Data must be collected as they are obtained and copied into case report forms clearly and legibly.

Erroneous data recorded in case report forms will be crossed out clearly and the new data will be copied next to the crossed out information, with the corrector’s initials, date and possibly a reason by the investigator or the authorised person who made the correction.

# bibliographical references

Reference List

1. Report of the Expert Committee on the Diagnosis and Classification of Diabetes Mellitus. Diabetes Care 1997;20:1183-97.

2. King H, Rewers M. Global estimates for prevalence of diabetes mellitus and impaired glucose tolerance in adults. WHO Ad Hoc Diabetes Reporting Group. Diabetes Care 1993;16:157-77.

3. Wild S, Roglic G, Green A, Sicree R, King H. Global prevalence of diabetes: estimates for the year 2000 and projections for 2030. Diabetes Care 2004;27:1047-53.

4. Drewnowski A, Specter SE. Poverty and obesity: the role of energy density and energy costs. Am J Clin Nutr 2004;79:6-16.

5. Charles MA, Eschwege E, Basdevant A. Monitoring the obesity epidemic in France: the Obepi surveys 1997-2006. Obesity (Silver Spring) 2008;16:2182-6.

6. Report of the Expert Committee on the Diagnosis and Classification of Diabetes Mellitus. Diabetes Care 1997;20:1183-97.

6 bis. Alberti GK, Eckel RH, Grundy SM et coll. Harmonizing the Metabolic Syndrome. A Joint Interim Statement of the International Diabetes Federation Task Force on Epidemiology and Prevention; National Heart Lung and Blood Institute; American Heart Association; World Heart Federation; International Atherosclerosis Society; and International Association for the Study of Obesity. *Circulation* 2009;120:1640-45.

7. The Diabetes Prevention Program (DPP): description of lifestyle intervention. Diabetes Care 2002;25:2165-71.

8. Pan XR, Li GW, Hu YH et al. Effects of diet and exercise in preventing NIDDM in people with impaired glucose tolerance. The Da Qing IGT and Diabetes Study. Diabetes Care 1997;20:537-44.

9. Chiasson JL. Acarbose for the prevention of diabetes, hypertension, and cardiovascular disease in subjects with impaired glucose tolerance: the Study to Prevent Non-Insulin-Dependent Diabetes Mellitus (STOP-NIDDM) Trial. Endocr Pract 2006;12 Suppl 1:25-30.

10. Hoerger TJ, Hicks KA, Sorensen SW et al. Cost-effectiveness of screening for pre-diabetes among overweight and obese U.S. adults. Diabetes Care 2007;30:2874-9.

11. Jacob S, Rabbia M, Meier MK, Hauptman J. Orlistat 120 mg improves glycaemic control in type 2 diabetic patients with or without concurrent weight loss. Diabetes Obes Metab 2009;11:361-71.

12. Anderson RA, Broadhurst CL, Polansky MM et al. Isolation and characterization of polyphenol type-A polymers from cinnamon with insulin-like biological activity. J Agric Food Chem 2004;52:65-70.

13. Broadhurst CL, Polansky MM, Anderson RA. Insulin-like biological activity of culinary and medicinal plant aqueous extracts in vitro. J Agric Food Chem 2000;48:849-52.

14. Jarvill-Taylor KJ, Anderson RA, Graves DJ. A hydroxychalcone derived from cinnamon functions as a mimetic for insulin in 3T3-L1 adipocytes. J Am Coll Nutr 2001;20:327-36.

15. Qin B, Nagasaki M, Ren M, Bajotto G, Oshida Y, Sato Y. Cinnamon extract (traditional herb) potentiates in vivo insulin-regulated glucose utilization via enhancing insulin signaling in rats. Diabetes Res Clin Pract 2003;62:139-48.

16. Qin B, Nagasaki M, Ren M, Bajotto G, Oshida Y, Sato Y. Cinnamon extract prevents the insulin resistance induced by a high-fructose diet. Horm Metab Res 2004;36:119-25.

17. Sheng X, Zhang Y, Gong Z, Huang C, Zang YQ. Improved Insulin Resistance and Lipid Metabolism by Cinnamon Extract through Activation of Peroxisome Proliferator-Activated Receptors. PPAR Res 2008;2008:581348.

18. Qin B, Nagasaki M, Ren M, Bajotto G, Oshida Y, Sato Y. Cinnamon extract prevents the insulin resistance induced by a high-fructose diet. Horm Metab Res 2004;36:119-25.

19. Altschuler JA, Casella SJ, MacKenzie TA, Curtis KM. The effect of cinnamon on A1C among adolescents with type 1 diabetes. Diabetes Care 2007;30:813-6.

20. Baker WL, Gutierrez-Williams G, White CM, Kluger J, Coleman CI. Effect of cinnamon on glucose control and lipid parameters. Diabetes Care 2008;31:41-3.

21. Blevins SM, Leyva MJ, Brown J, Wright J, Scofield RH, Aston CE. Effect of cinnamon on glucose and lipid levels in non insulin-dependent type 2 diabetes. Diabetes Care 2007;30:2236-7.

22. Khan A, Safdar M, Ali Khan MM, Khattak KN, Anderson RA. Cinnamon improves glucose and lipids of people with type 2 diabetes. Diabetes Care 2003;26:3215-8.

23. Mang B, Wolters M, Schmitt B et al. Effects of a cinnamon extract on plasma glucose, HbA, and serum lipids in diabetes mellitus type 2. Eur J Clin Invest 2006;36:340-4.

24. Vanschoonbeek K, Thomassen BJ, Senden JM, Wodzig WK, van Loon LJ. Cinnamon supplementation does not improve glycemic control in postmenopausal type 2 diabetes patients. J Nutr 2006;136:977-80.

25. Ziegenfuss TN, Hofheins JE, Mendel RW, Landis J, Anderson RA. Effects of a water-soluble cinnamon extract on body composition and features of the metabolic syndrome in pre-diabetic men and women. J Int Soc Sports Nutr 2006;3:45-53.

26. Hlebowicz J, Hlebowicz A, Lindstedt S et al. Effects of 1 and 3 g cinnamon on gastric emptying, satiety, and postprandial blood glucose, insulin, glucose-dependent insulinotropic polypeptide, glucagon-like peptide 1, and ghrelin concentrations in healthy subjects. Am J Clin Nutr 2009;89:815-21.

27. Solomon TP, Blannin AK. Changes in glucose tolerance and insulin sensitivity following 2 weeks of daily cinnamon ingestion in healthy humans. Eur J Appl Physiol 2009;105:969-76.

28. Solomon TP, Blannin AK. Changes in glucose tolerance and insulin sensitivity following 2 weeks of daily cinnamon ingestion in healthy humans. Eur J Appl Physiol 2009;105:969-76.

29. Hlebowicz J, Hlebowicz A, Lindstedt S et al. Effects of 1 and 3 g cinnamon on gastric emptying, satiety, and postprandial blood glucose, insulin, glucose-dependent insulinotropic polypeptide, glucagon-like peptide 1, and ghrelin concentrations in healthy subjects. Am J Clin Nutr 2009;89:815-21.

30. Khan A, Safdar M, Ali Khan MM, Khattak KN, Anderson RA. Cinnamon improves glucose and lipids of people with type 2 diabetes. Diabetes Care 2003;26:3215-8.

31. Mang B, Wolters M, Schmitt B et al. Effects of a cinnamon extract on plasma glucose, HbA, and serum lipids in diabetes mellitus type 2. Eur J Clin Invest 2006;36:340-4.

32. Vanschoonbeek K, Thomassen BJ, Senden JM, Wodzig WK, van Loon LJ. Cinnamon supplementation does not improve glycemic control in postmenopausal type 2 diabetes patients. J Nutr 2006;136:977-80.

33. Altschuler JA, Casella SJ, MacKenzie TA, Curtis KM. The effect of cinnamon on A1C among adolescents with type 1 diabetes. Diabetes Care 2007;30:813-6.

34. Blevins SM, Leyva MJ, Brown J, Wright J, Scofield RH, Aston CE. Effect of cinnamon on glucose and lipid levels in non insulin-dependent type 2 diabetes. Diabetes Care 2007;30:2236-7.

35. Baker WL, Gutierrez-William G, White CM, Kluger J, Coleman CI. Effect fo Cinnamon on Glucose Control and Lipid Parameters. Diabetes Care 8 A.D.;31:41-3.

36. Ziegenfuss TN, Hofheins JE, Mendel RW, Landis J, Anderson RA. Effects of a water-soluble cinnamon extract on body composition and features of the metabolic syndrome in pre-diabetic men and women. J Int Soc Sports Nutr 2006;3:45-53.

37. Morris BW, Macneil S, Hardisty CA, Heller S, Burgin C, Gray TA. Chromium homeostasis in patients with type II (NIDDM) diabetes. Journal of Trace Elements in Medicine and Biology 1999;13:57-61.

38. Pineau A, Guillard O, Risse JF. A study of chromium in human cataractous lenses and whole blood of diabetics, senile and normal population. Biological Trace Element Research 1992;32:133-8.

39. Anderson RA, Polansky MM, Bryden NA, Bhatena SJ, Canary JJ. Effects of supplemental chromium on patients with symptoms of reactive hypoglycemia. Metabolism 1987;36:351-5.

40. Anderson RA. Chromium, glucose intolerance and diabetes. Journal of the American College of Nutrition 1998;17:548-55.

41. Mertz W. Chromium in human nutrition: a review. Journal of Nutrition 1993;123:626-33.

42. Anderson RA, Cheng N, Bryden NA et al. Elevated intakes of supplemental chromium improve glucose and insulin variables in individuals with type 2 diabetes. Diabetes 1997;46:1786-91.

43. Balk EM, Tatsioni A, Lichtenstein AH, Lau J, Pittas AG. Effect of chromium supplementation on glucose metabolism and lipids: a systematic review of randomized controlled trials. Diabetes Care 2007;30:2154-63.

44. Zima T, Mestek O, Tesar V, Tesarova P, Zak A, Zeman M. Chromium levels in patients with internal diseases. Biochemistry and Molecular Biology International 1998;46:365-74.

45. Urberg M, Remel MB. Evidence for synergism between chromium and nicotinic acid in the control of glucose tolerance in elderly humans. Metabolism 1987;36:896-9.

46. Reddy VP, Garrett MR, Perry G, Smith MA. Carnosine: a versatile antioxidant and antiglycating agent. Sci Aging Knowledge Environ 2005;2005:e12.

47. Szwergold BS. Carnosine and anserine act as effective transglycating agents in decomposition of aldose-derived Schiff bases. Biochem Biophys Res Commun 2005;336:36-41.

48. Ferraris RP, Diamond J, Kwan WW. Dietary regulation of intestinal transport of the dipeptide carnosine. Am J Physiol 1988;255:G143-G150.

49. Chan WK, Decker EA, Chow CK, Boissonneault GA. Effect of dietary carnosine on plasma and tissue antioxidant concentrations and on lipid oxidation in rat skeletal muscle. Lipids 1994;29:461-6.

50. Lee YT, Hsu CC, Lin MH, Liu KS, Yin MC. Histidine and carnosine delay diabetic deterioration in mice and protect human low density lipoprotein against oxidation and glycation. Eur J Pharmacol 2005;513:145-50.

# Appendixes

## Appendixe 1: Lettre d’information version 1.2

## Appendixe 2: Consentement éclairé version 1.3

## Appendixe 3: Copie de l’attestation d’assurance

## Appendixe 4: Autorisation du lieu de recherche

## Appendixe 5: Copies de l’avis favorable du CPP datant du 20/12/2010 et de l’Afssaps datant du 2/11/2010

## Appendixe 6: Questionnaires TFEQ et d’activité physique

## Appendixe 7: Carnet alimentaire

## Appendixe 8: Annonce « Métro »

## Appendixe 9: Déclaration d’Helsinki

## Appendixe 10: BPC

## Appendixe 1: Lettre d’information version 1.2

## Appendixe 2: Consentement éclairé version 1.3

## Appendixe 3: Copie de l’attestation d’assurance

## Appendixe 4: Autorisation du lieu de recherche

## Appendixe 5: Copies de l’avis favorable du CPP datant du 20/12/2010 et de l’Afssaps datant du 2/11/2010

## Appendixe 6: Questionnaires TFEQ et d’activité physique

## Appendixe 7: Carnet alimentaire

## Appendixe 8: Annonce « Métro »

## Appendixe 9: Déclaration d’Helsinki

## Appendixe 10: BPC

1. CRNH: National Centre for research in Human Nutrition [↑](#footnote-ref-2)
2. AFSSAPS: French Health Products Safety Agency [↑](#footnote-ref-3)
3. PCH: Public health code [↑](#footnote-ref-4)
4. APHP: Public Assistance Hospitals of Paris [↑](#footnote-ref-5)
5. CNIL: National Commission on data processing and freedoms [↑](#footnote-ref-6)
