# Supplementary material for: A Dietary Supplement Containing Cinnamon, Chromium and Carnosine Decreases Fasting Plasma Glucose and Increases Lean Mass in Overweight or Obese Pre-Diabetic Subjects: A Randomized, Placebo-Controlled Trial
Source: PLoS One. 2015 Sep 25;10(9):e0138646. doi: 10.1371/journal.pone.0138646 (PMC4583280; doi:10.1371/journal.pone.0138646)
Supplement: S2 Protocol — (DOC) [file pone.0138646.s003.doc]

*ESSAI CLINIQUE D’EFFICACITE*

***Effet du complément alimentaire  Glycabiane sur le contrôle glycémique de sujets pré-diabétiques en surpoids ou OBESES.***

Titre abrégé : *Glycabiane* *et le contrôle glycémique des pré-diabétiques en surpoids.*

**Version :** 1.2 **en date du : 06/04/2012**

**Promoteur**

**Laboratoire PILEJE**

37 Quai de Grenelle, Tour Pollux, 75738 Paris Cedex 15

RCS PARIS 490 450 452

E-mail : s.bieuvelet@pileje.com

**Investigateur principal** :

Professeur Karine Clément

Service de Nutrition

Groupe Pitié Salpêtrière

47-83 boulevard de l’hôpital

Pavillon Husson Mourler

75651 Paris Cedex 13

Tel : 01.42.17.79 19

Mail : karine.clement@psl.aphp.fr

**Co-investigateur** :

Dr Salwa Rizkalla

INSERM U872 (Eq 7), CRNH Ile de France

Service de Nutrition

Bâtiment Benjamin Delessert

Hôpital Pitié Salpêtrière

83 Bd de l’Hôpital

75013 Paris

Tel : 01.42.17.79.81

Mail : salwa.rizkalla@psl.aphp.fr

**Lieu de réalisation de l’étude** :

INSERM U872 (Eq 7), CRNH Ile de France

Service de Nutrition

Bâtiment Benjamin Delessert

Hôpital Pitié Salpêtrière

83 Bd de l’Hôpital

75013 Paris

**Résumé**

| Titre | Effet du complément alimentaire Glycabiane, sur le contrôle glycémique de sujets pré-diabétiques en surpoids. |
| --- | --- |
| Promoteur | PILEJE – 37 quai de Grenelle – 75015 Paris |
| Investigateur Coordinateur | Professeur Karine Clément  Service de Nutrition  Groupe Pitié Salpêtrière  47-83 boulevard de l’hôpital  Pavillon Husson Mourler  75651 Paris Cedex 13 |
| Version du protocole | Version n°1.2 |
| Justification / contexte | Le prédiabète correspond à un état où les taux de glycémie sont plus élevés que la normale, mais sans être suffisamment élevés pour justifier un diagnostic de diabète de type 2 (c’est-à-dire un taux de glycémie à jeun de 7,0 mmol/L ou plus). Toutes les personnes présentant un prédiabète ne développeront pas un diabète de type 2, mais une bonne proportion d’entre elles le développera. Le risque de développer un diabète de type 2 augmente avec l’âge. Une modification des habitudes de vie incluant une augmentation de l’activité physique d’intensité modérée et une alimentation équilibrée avec un régime hypocalorique en cas de surpoids et/ou d’obésité constituent la base de la prise en charge pour prévenir ou retarder l’évolution vers le diabète de type 2. Toutefois, ces modifications du mode de vie ne sont pas toujours faciles à suivre au long cours. Différentes études d’intervention nutritionnelles ont montré que la cannelle d’une part et le chrome d’autre part peuvent aider à lutter contre la résistance à l’insuline, avec ou sans modification du mode de vie. L’association cannelle/chrome pourrait donc aider à réguler la glycémie de sujets pré-diabétiques et limiter ainsi la survenu du diabète et de ses complications micro et macrovasculaires ainsi que l’escalade thérapeutique souvent observée chez ces patients. |
| Objectif Principal | Etudier l’influence d’une supplémentation de 4 mois avec Glycabiane sur la glycémie à jeun de sujets prédiabétiques en surpoids. |
| Objectifs Secondaires | Etudier l’influence d’une supplémentation de 4 mois avec Glycabiane sur :  - le taux d’hémoglobine glyquée (HbA1c)  - le profil lipidique (Cholestérol total, LDL, HDL, TG, etc.)  - les marqueurs de l’inflammation : taux circulants de l’IL6 et CRPus  - le poids, l’IMC, le tour de taille, le rapport taille sur hanche et la masse grasse totale  - la pression artérielle diastolique et systolique  - l’insulinoresistance  - la taille des adipocytes  - le statut inflammatoire du tissu adipeux (dosage de la sécrétion de la cytokine IL-6 et de l’adipokine, adiponectine)  - la sensibilité à l’insuline du tissu adipeux par étude de la phosphorylation d’Akt et de la secretion de l’adiponectine  Evaluer la tolérance d’une supplémentation de 4 mois avec Glycabiane |
| Critère de Jugement Principal | Glycémie à jeun de sujets prédiabétiques en surpoids avant et après l’intervention nutritionnelle |
| Critères de Jugement Secondaires | Etudier l’influence d’une supplémentation de 4 mois avec Glycabiane sur le profil lipidique (Cholestérol total, LDL, HDL, TG, etc.), le taux d’hémoglobine glyquée, les caractéristiques anthropométriques et sur la biologie du tissu adipeux (taille des adipocytes, statut inflammatoire, sensibilité à l’insuline).  Evaluer la tolérance d’une supplémentation de 4 mois avec Glycabiane  Evaluer la qualité de vie |
| Méthodologie / Schéma de l’etude | Essai monocentrique, randomisé, contrôlé, en double aveugle contre placebo |
| Critères d’Inclusion des Sujets | Hommes et femmes âgés de 25-65 ans :  - prédiabétiques : 1,00g/L ≤glycémie à jeun<1,26g/L  - surpoids ou obésité (IMC>25)  - bilans rénaux et hépatiques normaux  - affilié à un régime de sécurité sociale française,  - suffisamment coopérant pour se conformer aux impératifs de l'étude,  - immunocompétent (pas de VIH connu ni d’hépatite C),  - utilisation d’une méthode de contraception efficace chez les femmes en âge de procréer |
| Critères de Non-Inclusion des Sujets | - maladie thyroïdienne - hypogonadisme - antécédent de maladie musculo-squelettique, auto-immune ou neurologique - Hépatite C, HIV - traitement médicamenteux contre une maladie thyroïdienne, hypoglycémie, ou anti-coagulant - femme enceinte ou allaitante - sujet faisant l’objet d’une mesure de sauvegarde de justice - sujet ayant perçu 4500€ d’indemnités compensatrices au cours des 12 derniers mois. Cette information est à vérifier par l’investigateur, les sujets étant indemnisés dans la recherche - antécédent d’allergie à l’un des composants du produit expérimental - contre-indication à l’utilisation de xylocaïne comme anesthésiant local |
| Nombre de Patients | 50 sujets : 25 sujets par groupe |
| Durée de la Recherche | Durée de la période d’inclusion : 6 mois  Durée de la participation pour chaque patient : 6 mois  Durée totale de l’étude : 24 mois |
| Retombées attendues | Cette première étude contrôlée, randomisée en double aveugle versus placebo d’une nouvelle association cannelle/chrome est un espoir important pour arriver à réguler la glycémie à jeun de sujets prédiabétiques en surpoids avec un produit nutritionnel et à dose physiologique afin de limiter l’évolution d’un prédiabète vers un diabète de type 2. |

SOMMAIRE

[I. Justification de l’essai 11](#__RefHeading___Toc321477298)

[A. Dénomination et description de la maladie 11](#__RefHeading___Toc321477299)

[B. Les traitements ou priseS en charge déjà existant et leurs limites 12](#__RefHeading___Toc321477300)

[1. Modification des habitudes de vie et de l’alimentation 12](#__RefHeading___Toc321477301)

[2. Interventions pharmacologiques 12](#__RefHeading___Toc321477302)

[3. Interventions nutritionnelles 14](#__RefHeading___Toc321477303)

[C. La nouvelle prise en charge proposée 20](#__RefHeading___Toc321477304)

[D. Bénéfices attendus et/ou risques prévisibles pour les personnes 21](#__RefHeading___Toc321477305)

[II. Protection des personnes 21](#__RefHeading___Toc321477306)

[A. Justification éthique du protocole 21](#__RefHeading___Toc321477307)

[B. Dispositions éthiques et réglementaires 21](#__RefHeading___Toc321477308)

[C. Comité de Protection des Personnes (CPP) 22](#__RefHeading___Toc321477309)

[D. AFSSAPS 22](#__RefHeading___Toc321477310)

[E. Assurance et financement 23](#__RefHeading___Toc321477311)

[F. Modalités de recueil du consentement des personnes 24](#__RefHeading___Toc321477312)

[G. Indemnités 24](#__RefHeading___Toc321477313)

[III. Objectifs 24](#__RefHeading___Toc321477314)

[A. Objectif principal 24](#__RefHeading___Toc321477315)

[B. Objectifs secondaires 25](#__RefHeading___Toc321477316)

[IV. Plan expérimental 25](#__RefHeading___Toc321477317)

[A. Type d’essai 25](#__RefHeading___Toc321477318)

[B. Catégorie de recherche : 25](#__RefHeading___Toc321477319)

[C. Exposé du plan expérimental 25](#__RefHeading___Toc321477320)

[V. Critères d’évaluation 27](#__RefHeading___Toc321477321)

[A. Critère principal 27](#__RefHeading___Toc321477322)

[B. Critères secondaires : 27](#__RefHeading___Toc321477323)

[VI. Sélection des personnes 27](#__RefHeading___Toc321477324)

[A. Population étudiée participante: 27](#__RefHeading___Toc321477325)

[1. Durée de la participation des personnes à la recherche : 27](#__RefHeading___Toc321477326)

[2. Description des personnes participantes : 27](#__RefHeading___Toc321477327)

[3. Nombre de personnes à inclure : 28](#__RefHeading___Toc321477328)

[B. Recrutement et sortie de l’essai 28](#__RefHeading___Toc321477329)

[1. Recrutement 28](#__RefHeading___Toc321477330)

[2. Sortie du protocole 28](#__RefHeading___Toc321477331)

[3. Période d’exclusion et participation à une autre recherche 29](#__RefHeading___Toc321477332)

[VII. Réalisation pratique du protocole 29](#__RefHeading___Toc321477333)

[A. Description détaillée des actes pratiqués sur les personnes : 29](#__RefHeading___Toc321477334)

[B. Description de l’organisation logistique générale de l’essai 31](#__RefHeading___Toc321477335)

[C. Calendrier prévisionnel de l’essai (Flow-Chart) 32](#__RefHeading___Toc321477336)

[VIII. Produit à l’étude 32](#__RefHeading___Toc321477337)

[A. Description du produit à l’étude 32](#__RefHeading___Toc321477338)

[1. Glycabiane 32](#__RefHeading___Toc321477339)

[2. Placebo 33](#__RefHeading___Toc321477340)

[B. Délivrance et compliance 33](#__RefHeading___Toc321477341)

[IX. Dispositifs médicaux 33](#__RefHeading___Toc321477342)

[X. Collection d’Echantillons biologiques d’origine humaine 35](#__RefHeading___Toc321477343)

[XI. vigilance de l’essai 35](#__RefHeading___Toc321477344)

[A. DEFINITIONS RELATIVES A LA VIGILANCE 35](#__RefHeading___Toc321477345)

[B. Rôle des investigateurs 36](#__RefHeading___Toc321477346)

[Evaluation de la gravité/lien de causalité des évènements indésirables 36](#__RefHeading___Toc321477347)

[C. Rôle du promoteur 37](#__RefHeading___Toc321477348)

[D. Suivi des patients en cas de survenue d’un évènement indésirable grave 38](#__RefHeading___Toc321477349)

[E. Comité de surveillance indépendant 38](#__RefHeading___Toc321477350)

[XII. Recueil et traitement des données 38](#__RefHeading___Toc321477351)

[A. Recueil des données 38](#__RefHeading___Toc321477352)

[B. Description du circuit des données avant la saisie informatique 38](#__RefHeading___Toc321477353)

[Recueil des données 38](#__RefHeading___Toc321477354)

[Saisie informatique des données 39](#__RefHeading___Toc321477355)

[XIII. Analyse statistique des données 40](#__RefHeading___Toc321477356)

[Nombre de sujets nécessaires 40](#__RefHeading___Toc321477357)

[XIV. Cnil 41](#__RefHeading___Toc321477358)

[XV. Archivage 41](#__RefHeading___Toc321477359)

[XVI. Communication 42](#__RefHeading___Toc321477360)

[XVII. Modifications substantielles du protocole 42](#__RefHeading___Toc321477361)

[XVIII. Confidentialité 42](#__RefHeading___Toc321477362)

[A. Accès aux données 42](#__RefHeading___Toc321477363)

[B. Données source 43](#__RefHeading___Toc321477364)

[C. Confidentialité des données 43](#__RefHeading___Toc321477365)

[D. Inscription au fichier national des personnes se prêtant à une recherche biomédicale 43](#__RefHeading___Toc321477366)

[XIX. Assurance qualité 44](#__RefHeading___Toc321477367)

[A. Engagement des investigateurs et du promoteur 44](#__RefHeading___Toc321477368)

[B. Assurance de Qualité 44](#__RefHeading___Toc321477369)

[C. Contrôle de la Qualité 44](#__RefHeading___Toc321477370)

[D. Cahier d’observation 44](#__RefHeading___Toc321477371)

[XX. Références bibliographiques 45](#__RefHeading___Toc321477372)

[XXI. Annexes 50](#__RefHeading___Toc321477373)

[A. Annexe 1 : Lettre d’information version 1.2 50](#__RefHeading___Toc321477374)

[B. Annexe 2 : Consentement éclairé version 1.3 50](#__RefHeading___Toc321477375)

[C. Annexe 3 : Copie de l’attestation d’assurance 50](#__RefHeading___Toc321477376)

[D. Annexe 4 : Autorisation du lieu de recherche 50](#__RefHeading___Toc321477377)

[E. Annexe 5 : Copies de l’avis favorable du CPP datant du 20/12/2010 et de l’Afssaps datant du 2/11/2010 50](#__RefHeading___Toc321477378)

[F. Annexe 6 : Questionnaires TFEQ et d’activité physique 50](#__RefHeading___Toc321477379)

[G. Annexe 7 : Carnet alimentaire 50](#__RefHeading___Toc321477380)

[H. Annexe 8 : Annonce « Métro » 50](#__RefHeading___Toc321477381)

[I. Annexe 9 : Déclaration d’Helsinki 50](#__RefHeading___Toc321477382)

[J. Annexe 10 : BPC 50](#__RefHeading___Toc321477383)

[A. Annexe 1 : Lettre d’information version 1.2 51](#__RefHeading___Toc321477384)

[B. Annexe 2 : Consentement éclairé version 1.3 52](#__RefHeading___Toc321477385)

[C. Annexe 3 : Copie de l’attestation d’assurance 53](#__RefHeading___Toc321477386)

[D. Annexe 4 : Autorisation du lieu de recherche 54](#__RefHeading___Toc321477387)

[E. Annexe 5 : Copies de l’avis favorable du CPP datant du 20/12/2010 et de l’Afssaps datant du 2/11/2010 55](#__RefHeading___Toc321477388)

[F. Annexe 6 : Questionnaires TFEQ et d’activité physique 56](#__RefHeading___Toc321477389)

[G. Annexe 7 : Carnet alimentaire 57](#__RefHeading___Toc321477390)

[H. Annexe 8 : Annonce « Métro » 58](#__RefHeading___Toc321477391)

[I. Annexe 9 : Déclaration d’Helsinki 59](#__RefHeading___Toc321477392)

[J. Annexe 10 : BPC 60](#__RefHeading___Toc321477393)

# Justification de l’essai

## Dénomination et description de la maladie

Le diabète de type 2 est une maladie dont l’étiopathogénie et la physiopathologie sont complexes. Il survient incontestablement sur un terrain génétique de prédisposition, et est favorisé par un mode de vie inapproprié. Le diabète de type 2 se caractérise par une hyperglycémie résultant de déficits de l’action de l’insuline ou de sa sécrétion, ou des deux conjugués (1). Cette hyperglycémie est associée, à des degrés divers et par des mécanismes encore mal connus, à des complications à long terme, touchant en particulier les yeux, les reins, les nerfs, le cœur et les artères. Cette maladie reste longtemps asymptomatique et survient typiquement après la cinquantaine, tout particulièrement chez des sujets en surpoids ou qui ont des antécédents familiaux de la même maladie.

L'absence de symptômes dans le diabète de type 2, fait qu'il est très difficile de déterminer la prévalence de cette pathologie, néanmoins, c'est une maladie qui ne cesse de progresser et le nombre de patient augmente d'année en année dans le monde entier y compris dans les pays en voie de développement.

Sur le plan mondial, le diabète de type 2 représente 90% des patients diabétiques, et ce chiffre doit passer de 135 à 300 millions entre 1995 et 2025 (2).

Aujourd'hui, la prévalence du nombre de diabétiques est plus élevée dans les pays industrialisés et elle le restera, mais l'augmentation prévue de cette prévalence d'ici 2025 est de 171% dans les pays en voie de développement et 41% dans les pays industrialisés. Les pays qui comptent le plus de diabétiques sont : l'Inde, la Chine et les Etats Unis, et cette proportion restera valable dans les 20 ans à venir (3).

A partir des demandes de remboursements pour médicaments antidiabétiques, la prévalence du diabète traité a été estimée à 3.95% en 2007, soit 2 500 000 personnes. Elle est plus importante dans les classes d’âge élevées, avec un maximum de 18,2% chez les hommes âgés de 75 à 79 ans, et de 13,2% chez les femmes de même âge. Il existe de grandes variations régionales à âge égal avec des taux élevés dans les départements d’Outre-Mer (entre 6 et 7%) et dans le Nord et le Nord-Est du pays (4,4% en Lorraine, 4,5% en Alsace et Champagne-Ardennes, 4,7% en Picardie, et 4,8% dans le Nord-Pas-De-Calais).

Cette prévalence augmente rapidement, en s’accroissant en moyenne de 5,7% par an (4). Elle résulte de la conjugaison de deux facteurs : les effets du vieillissement démographique et ceux de l’augmentation de la prévalence de l’obésité en France (5).

On peut considérer aujourd’hui comme établi que le diabète de type 2 est, sauf dans de rares cas, une maladie caractérisée par la conjonction d’une insulinorésistance (moindre sensibilité à l’action de l’insuline) et d’un défaut de sécrétion béta pancréatique. Plus précisément, l’installation d’un diabète apparaît comme un processus en deux temps (6) :

d’abord insulinorésistance, mais normoglycémie maintenue au prix d’une hyperinsulinémie « compensatrice » ; puis basculement dans le diabète proprement dit, quand le pancréas n’arrive plus à fournir la quantité d’insuline nécessaire à l’homéostasie des métabolismes insulinodépendants.

La première période, « prédiabétique », s’accompagne en général de diverses anomalies métaboliques potentiellement athérogènes ou thrombogènes, probablement dérivées de l’association insulinoresistance – hyperinsulinémie, et regroupées sous le nom de « syndrome métabolique ».

Le « pré-diabète » est donc un état intermédiaire entre une homéostasie du glucose normale et le diabète de type 2 avéré, incluant deux entités cliniques : l’hyperglycémie modérée à jeun (HMJ) et l’intolérance au glucose (IAG).

La glycémie à jeun anormale est définie par une glycémie à jeun ≥1,0 g/l, mais < 1,26 g/l. selon l'IDF (International Diabetes Federation) en partenariat avec l'AHA (American Heart Association), le NHLBI (National Heart Lung and Blood Institute), la société internationale d'athérosclérose et l'association internationale pour l'étude de l'obésité (6bis).

L’intolérance au glucose est défini quant à elle par une glycémie 2H après une charge orale de 75 g de glucose ≥1,40 g/l et < 2 g/l.

## Les traitements ou priseS en charge déjà existant et leurs limites

### Modification des habitudes de vie et de l’alimentation

Trois études prospectives ont démontré l’efficacité de la perte de poids et de l’activité physique sur la réduction du risque du diabète chez les patients avec intolérance au glucose.

**L’étude américaine  du DPP**, faite sur 3234 sujets intolérants au glucose, a comparé l’effet de la Metformine (traitement pharmacologique hypoglycémiant) à celui de modifications des habitudes de vie (réduction de l’apport calorique + exercice physique). Les résultats ont permis d’établir que la perte de poids (de 5 à 7%)  grâce à des modifications alimentaires (diminution des graisses saturées (<10% de l’apport total, augmentation de la consommation de fibres (de 15g/jour) soutenues par l’exercice physique (au moins 30 min/jour) permet de diminuer le risque de diabète de 58% en trois ans contre 31% avec la Metformine (7)

**L’étude Da Qing en Chine** (1997), menée sur 577 sujets intolérants au glucose, a montré que les interventions diététiques et/ou l’exercice physique suivi(es) pendant 6 ans, diminuaient globalement le risque de diabète de 39 % (8).

**L’étude de prévention finlandaise (DPS)** faite sur 522 sujets insulino-résistants a montré qu’une perte de poids de 5 % obtenue grâce à un programme intensif de diète (diminution de l’apport de graisses saturées et apport de fibres) et d’exercices a permis de réduire l’incidence du diabète de 58%.

En 2007, une méta-analyse [10] a évalué l'efficacité de diverses interventions, changement du mode de vie (régime et exercice) et prise de médicaments sur le risque de développer un diabète de type 2. Les modifications d'un mode de vie, régime et exercice physique, visant à prévenir ou à réduire l'obésité, ont nettement diminué le risque de diabète, sans avoir d'effets indésirables.

### Interventions pharmacologiques

Les médicaments qui réduisent l’apport du glucose (Metformine et Acarbose) ne modifient pas la progression de la maladie mais retardent seulement son apparition.

Par ailleurs, toutes les interventions pharmacologiques ont des effets secondaires dont il faut tenir compte.

#### La Metformine

Dans l’étude américaine du DPP, un tiers des patients présentant une intolérance au glucose a pris pendant trois ans de la metformine. La progression vers le diabète de type 2 semble avoir diminué de 31% par rapport au groupe contrôle. La médication semblant plus efficace dans les sous-groupes de patients de moins de 60 ans et avec un IMC de plus de 30 kg/m². L’arrêt du médicament entraîne une perte de l’efficacité de prévention de plus de 75 %. Les principaux effets secondaires sont les nausées, la diarrhée et les douleurs abdominales qui se retrouvent chez plus de 25 % des sujets. Le médicament est contre-indiqué en présence d’insuffisance rénale (clearance < 30 ml/min).

#### L’acarbose

L’acarbose est un hypoglycémiant oral dont le mode d’action est de retarder l’absorption du glucose au niveau intestinal par inhibition de l’alphaglucosidase. Dans l’étude STOP-NIDDM, la prise d’acarbose réduit significativement le risque d’évolution vers le diabète de type 2 de patients présentant une intolérance au glucose de 25% et ceci quelque soit l’âge ou le BMI (9). Ce médicament semble diminuer aussi les complications cardiovasculaires chez les prédiabétiques. Son utilité est limitée par les flatulences et les diarrhées associées à son usage régulier. Dans l’étude STO-NIDDM, 25% des sujets traités ont arrêté leur traitement du fait des effets secondaires du médicament.

#### Les thiazolidinediones

L’étude DREAM a voulu démontrer chez plus de 1500 prédiabétiques l’utilité soit d’un médicament utilisé généralement pour le traitement de l’insuffisance cardiaque ou de l’hypertension (le ramipril) ou encore d’un hypoglycémiant oral (la rosiglitasone : médication stimulant les PPAR gamma, ce qui amène une répartition des graisses viscérales et du foie vers le muscle et qui augmentent les transporteurs du glucose au niveau musculaire entre autres modes d’action de cette médication complexe ). Le ramipril, bien que diminuant légèrement les glycémies,  n’a pas permis de diminuer l’apparition du diabète. La rosiglitasone a diminué le risque d’évolution d’une intolérance au glucose vers un diabète de type 2 de 62 % par rapport au groupe contrôle. La rosiglitasone semblait plus efficace chez les patients  au tour de taille élevé. Cependant, sur les 5 ans de suivi, seuls 20% des sujets évoluaient vers un diabète de type 2. Par ailleurs, une faible augmentation du risque cardiovasculaire  et une **augmentation certaine du risque** de fractures  distales semblent tempérer pour le moment  l’utilisation de la rosiglitasone chez de jeunes patients en prévention. De plus cette  prise en charge thérapeutique est associée à un gain de poids constant (10).

#### Orlisat

Ce médicament est un inhibiteur de lipase utilisé pour traiter l’obésité qui améliore la glycémie en stimulant la perte de poids. Les mécanismes possibles seraient une amélioration de la sensibilité à l’insuline, un ralentissement et une digestion incomplète des lipides, une diminution des acides-gras non estérifiés post-prandiaux plasmatiques, une réduction du tissu adipeux viscéral et une stimulation de la sécrétion du peptide-1 glucagon-like dans l’intestin grêle (11). Les principaux effets secondaires sont les selles huileuses, l’incontinence fécale, des diarrhées impérieuses : selles fréquentes ou urgentes, un météorisme, des douleurs abdominales à type coliques et des flatulences.

### Interventions nutritionnelles

#### La cannelle dans la prévention de l’hyperglycémie

La cannelle est une épice traditionnellement utilisée dans certains pays asiatiques dans le traitement du diabète. Depuis 1990, différentes études in vitro et in vivo chez l’animal ont montré son intérêt dans la régulation du métabolisme du glucose (12-16). In vitro dans des lignées de cellules adipocytaires, la cannelle sous forme d’extrait stimule la captation du glucose et la synthèse de glycogène en stimulant la phosphorylation du récepteur de l’insuline et la glycogène synthase (14). L’extrait de cannelle est également capable de moduler l’expression du facteur de transcription PPARγ et de ses gènes cibles, dont LPL, CD-36 et GLUT-4 qui sont impliqués dans le métabolisme des lipides (17). In vivo chez le rat, l’extrait de cannelle stimule l’utilisation du glucose de façon dose-dépendante en stimulant la phosphorylation de la sous-unité beta du récepteur de l’insuline, celle d’IRS-1 (Insulin Receptor Substrate-1) et l’association d’IRS-1 avec la PI3K (Phosphatidyl Inositol 3 Kinase) (15;18).

Un des principes actifs des extraits de cannelle serait un polymère de méthylhydroxychalcones capable de mimer l’insuline et d’induire des effets « insulin-like » (14), en particulier les OPC-A (polymère polyphénolique de type A, soluble dans l’eau) (12).

Chez l’homme, 6 essais cliniques et une méta-analyse concernant les effets de la cannelle sur le métabolisme du glucose ont été publiés. La plupart ont recherché un effet de la cannelle chez des diabétiques de type 2 (19-24) (tableau). Une seule étude s’est intéressée aux personnes souffrant du syndrome métabolique (25) tandis que deux autres études se sont intéressées à des sujets sains (26;27). Les résultats des études sur le diabète de type 2 sont controversés, certains concluant à un effet de la cannelle sur la glycémie à jeun, l’HbA1c et éventuellement le profil lipidique et d’autres ne montrant aucun effet significatif (tableau). Il est à noter que tous ces essais présentent des biais méthodologiques. Ils portent assez souvent sur un nombre restreint de sujets, les données biologiques à l’état basal ne sont pas toujours standardisées, la durée de la supplémentation ne permet pas toujours d’observer des variations du taux d’HbA1c, etc. Par ailleurs, la supplémentation en cannelle était toujours différente que ce soit en termes de dose ou en termes de durée de complémentation. Or, les effets de la cannelle semblent à la fois dépendre de la dose ingérée, de la durée de la supplémentation et des paramètres glucido-lipidiques initiaux des patients. D’autres études randomisées, contrôlées en double aveugle contre placebo seront donc nécessaires pour déterminer le rôle exact de la cannelle sur les paramètres glucido-lipidiques des diabétiques de type 2.

Une étude en double aveugle contrôlée contre placebo a déterminé les effets d’une supplémentation avec un extrait de cannelle hydrosoluble^15^ sur la glycémie à jeun d’hommes et de femmes avec un syndrome métabolique ou prédiabétique. Après ajustement sur l'âge, la glycémie a jeun, la pression artérielle et les niveaux d'activité physiques habituels, 12 sujets ont consommé 500 mg par jour d’un extrait aqueux de cannelle contenant 1% d’OPCA et 10 un placebo pendant 12 semaines. Les résultats montrent une diminution significative de la glycémie à jeun (- 8,4 % : 116,3+/-12,8 mg/dl au début vs 106,5+/-20,1 mg/dl à la fin de l’étude) chez les sujets ayant consommé l’extrait de cannelle. Les sujets ont également vu leur pression sanguine systolique baisser (-3,8%) ainsi que leur masse grasse corporelle (-0.7%) tandis que leur masse maigre avait augmenté de 1,1%.

Récemment, l’équipe de Salomon a évalué l’effet de l’ingestion de 3 g de cannelle par jour pendant 14 jours sur la tolérance au glucose et la sensibilité à l’insuline de 8 hommes en bonne santé âgés de 25 ± 1 ans et avec un IMC de 24± 0,7. Les 14 sujets ont consommé soit le Placebo soit le supplément de cannelle pendant 14 jours alternativement. La supplémentation avec le Placebo a été poursuivie 5 jours au-delà des 14 jours de complémentation. Les tests de tolérance au glucose ont été réalisés à J0, 1, 14, 16, 18 et 20. L’ingestion de cannelle réduit significativement la réponse glycémique au test de tolérance au glucose à J1 (-13 ,1±6.3% vs J0, p<0,05) et J14 (-5,5±8.1% vs J0, p=0,09). L’ingestion de cannelle permet également de réduire la réponse insulinique à J14 (-27,1± 6,2% vs J0, p <0,05) et la sensibilité à l'insuline (p<0.05 à J14 vs J0). Ces effets positifs s'arrêtaient après l'arrêt de la supplémentation suggérant que la cannelle peut améliorer le contrôle glycémique et la sensibilité à l’insuline mais que ces effets sont réversibles(28). Enfin, une autre étude sur 15 sujets sains a montré un effet bénéfique de l'ingestion de 3 g de cannelle mélangé à un gâteau de riz, sur l’insulinémie postprandiale (vs gâteau de riz sans cannelle). Aucun effet n'a été retrouvé avec 1g de cannelle et aucun effet n’a été retrouvé sur la glycémie(29).

En conclusion, on manque encore de données scientifiques probantes pour conclure à un quelconque effet de la cannelle sur la glycémie bien qu’il semble que la cannelle ait un léger effet hypoglycémiant. Pour ce qui est du taux d’HbA1c, son effet semble négligeable mais d’autres études cliniques randomisées contre placebo sont nécessaires pour en évaluer l’impact réel.

| **Références bibliographiques** | **Type d’étude et nombre de sujets (N)** | **Population cible** | **Intervention** | **Résultats** | **Commentaires** |
| --- | --- | --- | --- | --- | --- |
| Khan A. et al. *Diabetes Care* 26 :3215-3218, 2003(30) | Essai clinique randomisée contre placebo  N=60 | Hommes et femmes diabétiques de type 2 (DT2) âgés de 52.2±6.32 ans | 6 groupes de 10 sujets :  Gr 1 : 1g de cannelle/j  Gr 2 : 2g de cannelle/j  Gr 3 : 3g de cannelle/j  Gr 4 : 1g de placebo/j  Gr 5 : 2g de placebo /j  Gr 6 : 3g de placebo /j  Supplémentation pendant 40 jrs puis 20 jrs sans complément | La glycémie à jeun est significativement diminuée de 18 à 29%, les triglycérides de 23 à 30%, le cholestérol total de 13 à 26 % et le cholestérol LDL de 10 à 24 % en réponse à la supplémentation en cannelle. Aucune modification significative des niveaux de cholestérol HDL n’est observée. Les niveaux de glucose et de triglycérides augmentent modestement après l'arrêt du traitement mais restent en dessous des valeurs basales. Ceux du cholestérol total et du cholestérol LDL continuent de baisser. Aucun effet secondaire n’a été rapporté. | L’étude à été réalisée avec des patients Pakistanais ayant un diabète mal contrôlé et pour lesquels les taux de glucoses sanguins basaux étaient significativement plus élevés que ceux généralement observés dans les autres études cliniques sur le diabète de type 2. Les patients diabétiques ont généralement un meilleur contrôle glycémique. Il est donc probable que les résultats observés dans cette étude surestiment les effets positifs de la cannelle. Par ailleurs, l’étude est limitée par la taille de l’échantillon (60 sujets au total : 10 par groupe) et l’absence de standardisation des 6 groupes sur les apports alimentaires. |
| Mang B. et al. *Eur J Clin Invest* 2006 ; 36(5) :340-344(31) | Etude prospective, randomisée, en double aveugle versus placebo  N=79 | Hommes et femmes allemands DT2 | 2 groupes :  Gr 1 : 112 mg d’extrait aqueux de C. cassia équivalent à 1g de cannelle, 3 fois/jr pendant 4 mois  Gr 2 : placebo 3 fois/jr pendant 4 mois | La supplémentation à entraînée une diminution significative de la glycémie à jeun (- 10,3% ± 13.2%) chez les diabétiques qui ont reçu l’extrait aqueux de cannelle comparé à ceux qui ont consommé le placebo (- 3.37 ± 14.2%) (p=0.046).  Aucune variation significative du taux d’HbA1c n’a été observée. Aucun effet secondaire n’a été rapporté. | Bien que les auteurs concluent a un effet modéré de la cannelle sur la glycémie à jeun de diabétiques de type 2 mal contrôlé, il est a noté que cette étude comporte différents facteurs limitant. Tout d’abord, la taille de l’échantillon (n=65). Puis, la fréquence des mesures de la glycémie. Il aurait pu être opportun de faire des mesures rapprochées pour confirmé la tendance observée. |
| Vanschoonbeek K. et al.*J. Nutr.* 136 :977-980. 2006(32) | Etude, contrôlée contre placebo, mais non randomisée  N=25 | Femmes DT2, ménopausées âgées de 62,9+/1,5 ans avec un BMI de 30,4+/- 0, 9kg/m2 | 2 groupes :  Gr 1 (n=13) : 1g de cannelle/j pendant 6 semaines  Gr 2 (n=12) : 1g de placebo/j pendant 6 semaines | Après 6 semaines de complémentation, aucune différence significative entre les groupes complémenté et placebo n’a été observée sur les niveaux d’HbA1c ni la glycémie à jeun. | Certains biais méthodologiques peuvent être soulignés. Tout d’abord, il s’agissait d’une étude ouverte, non randomisée. Ensuite, l’étude porte uniquement sur des femmes ménopausée diabétiques de type 2 et non sur l’ensemble de la population de diabétiques de type 2 et sur un petit nombre de sujets (25 en tout). Enfin, la durée de l’étude (6 semaines) n’était peut-être pas suffisante pour voir des variations de l’HbA1c. Par ailleurs, la glycémie à jeun initiale des patientes incluses dans l’étude était inférieure à celle observée dans les autres études. |
| Altschuler JA. Et al. *Diabetes Care* 30 :813-816, 2007.(33) | Etude randomisée en double aveugle contre placebo  N=75 | Adolescents diabétiques de type 1 | Ingestion d’1g de cannelle ou de placebo/j pendant 90 jours | Aucune différence significative n’a été observée sur les niveaux d’hémoglobine glyquée (8.8 vs 8.7, p=0.88) ni sur l’insulinémie entre le début et la fin de la complémentation. | L’absence de production d’insuline chez les diabétiques de type 1 pourrait être à l’origine de l’absence d’effet de la cannelle. En effet, de nombreuses études in vitro et in vivo chez le rongeur suggèrent que la cannelle pourrait agir en stimulant la production endogène d’insuline. Par ailleurs, il est possible que les sujets aient consommé une quantité insuffisante de cannelle. Les études chez les diabétiques de type 2 montrent un effet intéressant de la cannelle pour des doses élevées, de l’ordre de 1.5 à 3g de cannelle par jour. La durée de la supplémentation peut également être en cause puisque le renouvellement des globules nécessite 120 jours et que la supplémentation n’a durée que 90 jours. |
| Blevins SM et al. *Diabetes Care* 30 :2236-2237, 2007. (34) | Etude randomisée en double aveugle contre placebo  N=57 | Diabétiques de type 2, américains en moyenne âgés de 63 ans. | 2 groupes :  Gr 1 (n=29) : 1g de cannelle/j pendant 3 mois  Gr 2 (n=28) : 1g de placebo/j pendant 3 mois | Aucune différence significative n’a été observée sur la glycémie à jeun et le taux d’HbA1c. |  |
| Baker WL. Et al. *Diabetes Care* 31 :41-43, 2008.(35) | Méta-analyse des essais cliniques sur l’effet de la cannelle chez de sujets DT2  N=282 | Diabétiques de type 2 | 5 essais cliniques | Les résultats montrent que l’ingestion de cannelle ne modifie pas significativement l’HbA1c, la glycémie à jeun ou les variables lipidiques plasmatiques. Les auteurs en concluent que la cannelle n’a pas d’effet significatif sur les paramètres glucido-lipidiques des diabétiques de type 1 et 2. | Sur les 5 essais cliniques sélectionnés (N=282), une correspondait à un essai non randomisé tandis qu’une autre avait pour cible des adolescents diabétiques de type 1. les 3 autres études cliniques randomisées contre placebo incluaient 296 patients en tout. Les doses de cannelle ingérées et durées de complémentation variaient d’une étude à l’autre de même que les populations cibles (diabète plus au moins bien équilibré, etc.) |
| Ziegenfuss TN. Et al. J Int Soc Sports Nutr 3 : 45-53, 2006.(36) | Etude randomisée en double aveugle contre placebo  N=22 | Sujets pré-diabétiques, polymétaboliques | 2 groupes :  Gr 1 (n=12) : 500mg d’extrait de cannelle titré à 1% d’OPCA/j pendant 3 mois  Gr 2 (n=10) : 500mg de placebo/j pendant 3 mois | Les résultats montrent une diminution significative de la glycémie à jeun (- 8.4 % : 116,3+/-12,8 mg/dl au début vs 106,5+/-20,1 mg/dl à la fin de l’étude) chez les sujets ayant consommé l’extrait de cannelle, p<0.05. Les sujets ont également vu leur pression sanguine systolique (-3.8%) ainsi que leur masse grasse corporelle (-0.7%) baisser tandis que leur masse maigre avait augmenté de 1.1%. |  |

#### Le chrome et le métabolisme glucidique

Le chrome est un élément essentiel pour les métabolismes glucidique et lipidique. Chez l'animal, il est nécessaire pour maintenir une tolérance au glucose normale. Chez l'humain, les signes cliniques associés à une déficience en chrome sont multiples : glycémie et taux d'insuline élevés, hypertriglycéridémie et hypercholestérolémie, mais aussi troubles nerveux et mnésiques (patient en nutrition parentérale).

Des patients diabétiques présentent des taux de chrome dans le sang inférieurs de 33 % à ceux des sujets sains, mais en revanche ont des taux d'excrétion urinaire plus élevés (100 %)(37). Cette excrétion est peut-être liée à une hyper filtration glomérulaire ou des lésions rénales (phénomène connu chez les diabétiques de type 1 mais controversé chez les DNID). Chez des patients atteints de rétinopathies et autres complications liées au diabète, les taux de chrome semblent être inférieurs à ceux de diabétiques ne présentant pas ces troubles associés. De plus, le dosage du chrome dans le cristallin de patients présentant une rétinopathie fait apparaître des taux semblables à ceux observés chez les personnes âgées, et plus faibles que les contrôles(38).

La relation inverse entre taux de chrome sérique et pathologie diabétique n'est pas causale mais souligne l'essentialité du chrome dans la gestion de l'homéostasie glycémique. Il est fort probable que sans être la cause unique des signes cliniques observés, la déficience en chrome puisse aggraver ou accélérer l'évolution de la maladie. Par ailleurs, différents facteurs peuvent affecter l'absorption et l'excrétion du chrome : le stress (via le cortisol), la consommation de sucres rapides, l'activité physique peuvent induire une perte accrue en chrome. Ainsi, les patients présentant des dysfonctionnements glycémiques seraient donc davantage exposés à une altération du métabolisme du chrome. Par conséquent, un apport en chrome suffisant pour restaurer un taux "normal" dans le sang pourrait être bénéfique, comme le suggère les études de complémentation.

Le chrome semble agir comme régulateur de l'homéostasie glycémique : chez des patients en hypoglycémie réactive, le chrome (200 µg de chlorure/jour) permet d'augmenter la glycémie et de réduire les symptômes hypoglycémiants(39). Chez des patients diabétiques, présentant une hyperglycémie postprandiale anormale, le chrome permet de réduire la glycémie et de moduler l'insulinémie. En revanche, chez des patients ne présentant pas d'altération de leur statut glycémique, la supplémentation en chrome reste inactive.

Des études de supplémentation en chrome rapportent des résultats variables sur la glycémie à jeun et la tolérance au glucose. Il n'y a pas eu d'étude dose-effet réalisée chez l'humain mais il semblerait que les études où l'apport en chrome est inférieur à 5 µmol/jour fournissent des résultats plus variables que celles utilisant des doses plus élevées. De plus, la forme sous laquelle est apporté le chrome semble être importante, le dérivé picolinate étant plus efficace que le dérivé chlorure(40;41). La revue de Mertz(41) montre qu'il n'existe que quelques études ne permettant pas de conclure sur les effets bénéfiques du chrome chez les diabétiques, mais que les études apportant une dose de chrome trivalent >400 µg par jour pendant plusieurs mois (min. 2-4 mois) réduisent l'insulino-résistance, améliorent la tolérance au glucose. Notamment, une étude récente de supplémentation de 2 à 4 mois chez 180 diabétiques et 83 contrôles montre une réduction de la glycémie à jeun et post-prandiale (-20 et 15 %). De même, l'insulinémie à jeun et post-prandiale sont abaissées (- 25-30 %). Il y a donc une régulation de la glycémie et une augmentation de la tolérance au glucose chez des DNID(42). En 2007, une grande méta-analyse a répertorié 41 essais cliniques (N=1198) ayant évalué l’effet de différentes formes de chrome sur le métabolisme glucido-lipidique. Les doses testées variaient de 200 à 1000µg par jour pendant 2 à 26 semaines. Parmi ces 41 essais, 14 (n=431) ont suivi des diabétiques de type 2 avec un taux d’HbA1c basal variant de 7,0% à 10,2%. Les résultats montrent que la supplémentation en picolinate de chrome ou la levure de bière à des doses variant de 200 à 1000µg par jour pendant 2 à 26 semaines réduit le taux d’HbA1c de 0,6% (IC_95%_ : -0,9% à -0,2%) et la glycémie à jeun d’1mmol/L (IC_95%_ : -1,4 à -0,5)(43). Il est cependant important de noter que dans cette méta-analyse, la moitié des études inclus sont de mauvaise qualité et utilisent différentes formes et doses de chrome dans des populations qui peuvent consommer du chrome dans leur alimentation. Parmi les 41 études répertoriées, aucun effet secondaire n’a été rapporté.

Le mode d'action du chrome dans l'homéostasie glycémique est mal connu. D'une part, il semble que le chrome forme avec l'acide nicotinique (vitamine B3), des acides aminés (cystéine, glycine, glutamine) et le glutathion un complexe, le Glucose Tolerance Factor, qui présente in vitro des effets bénéfiques sur l'utilisation du glucose et la liaison de l'insuline sur son récepteur(44). Chez l'humain, il n'a pu être clairement identifié, mais le manque d'acide nicotinique amoindrirait l'effet bénéfique du chrome sur la glycémie(45). Par ailleurs, le chrome agirait en augmentant l'efficacité insulinique : une supplémentation en chrome entraîne une augmentation de la liaison de l'insuline à son récepteur périphérique ce qui pourrait être dû à l'augmentation du nombre de récepteurs(42).

#### Carnosine

La carnosine (ou L-carnosine) est un dipeptide bêta-alanyl-L-histidine naturel trouvé principalement dans les tissus à longue durée de vie comme le muscle squelettique, le cerveau et le cristallin (plus de 20mM dans le muscle).. Elle se forme sous l’action de la carnosine synthétase qui réalise un pont entre les deux acides aminés alanine et histidine. Sa concentration dans les tissus résulte de l’équilibre entre sa synthèse par la carnosine synthétase et son inactivation par la carnisinase. Des taux élevés de carnosine sont présents dans les cellules à longue durée de vie (telles que les neurones).

La carnosine possède des propriétés antioxydante et antiglycante reconnues ainsi qu’une activité directe de limitation de liaison protéine–sucre : ou activité dite « anticrosslinking » (46).

La carnosine réagit avec les sucres comme le glucose, le galactose et le dihydroxyacétone pour former de la carnosine glyquée (non toxique pour l’organisme qui peut l’éliminer). Le plus réactif de ces trois sucres est le dihydroxyacétone : la carnosine réagit avec celui-ci plus rapidement qu’avec la lysine. De plus, la carnosine inactive les protéines glyquées par le dihydroxyacétone. Ainsi, la carnosine permet de réduire la glycation des protéines et la formation des AGEs (Advanced Glycation End products) en améliorant la reconnaissance de ceux-ci par leur récepteur. Par ailleurs, une étude très récente (47) a mis en évidence que la carnosine (20mM) est capable d’intervenir dans la déglycation des bases de Schiff (transglycation) catalysant la réaction de déglycation et génèrant des groupements aminés.

Boldyrev a montré que l'absorption de la carnosine dans l’alimentation est de 30 à 70 % (selon la quantité de divers acides aminés dans le repas) et celui de L-carnosine pure de plus de 70%. La plupart de l'absorption se produit dans le petit intestin (jéjunum, mais pas dans l'iléum) (48). Une étude sur le rat a mis en évidence qu’une supplémentation en carnosine (0,0875% du régime) associée à de la vitamine E (50 ppm) augmentait la concentration de carnosine dans le foie de 1,5 fois (49). Il en est de même chez la souris. Une étude réalisée sur des souris diabétiques (induit par streptozotocine) complémentées avec 0,5g/L ou 1g/L de carnosine dans leur eau pendant 4 semaines a révélé une augmentation de la concentration en carnosine, à la fois dans le plasma, dans le cœur et dans le foie et une diminution de la quantité de malone dialdéhyde (un produit terminal de la lipoperoxydation hautement réactif) produite (50).

Une supplémentation en carnosine pourrait donc permettre d’augmenter les concentrations plasmatiques et tissulaires en carnosine, et pourrait ainsi diminuer la glycation. A ce jour, il n’y a pas d’essai clinique publié sur l’effet d’une supplémentation en L-carnosine sur la glycation des protéines chez les diabétiques de type2.

## La nouvelle prise en charge proposée

Plusieurs études ont montré le bénéfice des mesures hygiéno-diététiques sur la progression d’une intolérance au glucose ou d’un prédiabète vers le diabète de type 2. Néanmoins, force est de constater que ces modifications du mode de vie (surveillance diététique étroite et pratique régulière de sport), sont souvent difficiles à tenir au long court. Les difficultés peuvent s’expliquer par le manque de conviction, de motivation et d’assiduité de certains patients, mais aussi pour des raisons d’incompatibilité ou d’impossibilité pour d’autres patients à suivre ces recommandations (personnes âgées, présence d’une maladie cardiaque, conditions physiques inadaptées).

Lorsque les modifications de mode de vie sont mal suivies ou ne suffisent pas à normaliser la glycémie, il peut parfois être nécessaire de mettre en place une complémentation nutritionnelle et/ou un traitement médicamenteux.

Les traitements médicamenteux s’accompagnent souvent d’effets secondaires non négligeables et ne font que retarder le délai d’apparition du diabète de type 2.

Les données de la littérature sur la cannelle et le chrome apportent des arguments en faveur de l’effet de chacun de ces ingrédients alimentaires sur le métabolisme glucidique de sujets prédiabétiques en surpoids. La carnosine pourrait réduire la glycation des protéines.

Le complément alimentaire Glycabiane permet des apports simultanés d’OPC-A de cannelle, de chrome et de carnosine, dans des proportions qui devraient permettre d’observer un effet significatif sur le métabolisme des glucides de sujets prédiabétiques.

L’apport de chrome sous forme de Guanylor® est particulièrement intéressant car il devrait permettre une meilleure absorption intestinale que sous la forme de levure classiquement utilisée dans les compléments alimentaires. Une étude cinétique comparative de l’absorption du chrome sous forme de Guanylor®-Cr ou de levure riche en chrome a été menée par le laboratoire Pileje chez le rat vigile non déficient. Les résultats montrent que l’administration de Guanylor*®* Cr entraîne une augmentation de la concentration plasmatique en chrome significativement plus importante que celle observée avec la levure riche en chrome (Voir dossier technique, données confidentielles non publiées). Par ailleurs, la quantité de chrome éliminée dans les matières fécales est significativement moins importante avec le Guanylor® Cr qu’avec la levure riche en chrome suggérant une meilleure absorption du chrome sous forme de Guanylor®-Cr. Parallèlement, ChalCinn® est un extrait de cannelle spécifiquement développé et sélectionné par le laboratoire Pileje. Particulièrement riche en OPC-A (au moins 5mg d’OPC-A pour 100mg d’extrait) et à teneur réduite en coumarine (< 15 ppm pour éviter les effets anti-coagulants) et huiles essentielles (<2% pour conserver le statut de complément alimentaire), il devrait permettre une complémentation sur le long court avec un effet significatif sur le métabolisme des glucides.

Glycabiane est donc formulé pour optimiser les effets du chrome et des OPC-A de cannelle sur le métabolisme des glucides de sujets prédiabétiques en surpoids.

L’ambition de cet essai clinique mené au CRNH Ile-de-France, Service Nutrition à la Pitié Salpêtrière, est d’évaluer l’effet d’une supplémentation nutritionnelle avec le complément alimentaire Glycabiane sur la glycémie à jeun de sujets prédiabétiques en surpoids.

## Bénéfices attendus et/ou risques prévisibles pour les personnes

La participation à cette étude peut faire progresser le corps médical dans la compréhension de la prévention du diabète et l’élévation de la glycémie cependant le bénéfice pour le participant ne peut pas être précisément évalué, puisqu'il s'agit de l'objet de la recherche. L'état actuel des connaissances permet d'émettre l'hypothèse d'un intérêt du Glycabiane en terme de régulation de la glycémie à jeun chez les sujets pré-diabétiques qui consomment Glycabiane et limiter ainsi leur évolution vers un diabète de type 2 (avec des complications micro et macrovasculaires). Au cas exceptionnel où, pour un individu donné, l’étude mettrait en évidence un risque sanitaire, les données appropriées seraient communiquées au médecin investigateur qui les communiquerait de la façon la plus pertinente à la personne concernée, dans le cadre du respect des personnes et du secret médical.

- **Risques liés aux traitements**

Le produit testé est un complément alimentaire présenté sous forme de gélules contenant un extrait de cannelle, de carnosine et de chrome. Il est déjà commercialisé sous la marque Glycabiane par le laboratoire PiLeJe depuis 2008 sans qu’aucun effet indésirable n’ait été rapporté. Glycabiane a notamment fait l’objet d’une enquête d’observation où aucun effet secondaire n’a été détecté. Il n’existe pas a priori d’effets indésirables.

- **Risques liés aux investigations métaboliques.**

***Aspiration à l’aiguille de tissu adipeux :*** Il est réalisé sous anesthésie locale, le désagrément éventuel pouvant être une ecchymose généralement de taille modeste qui se résorbe sous quelques jours, exceptionnellement un petit hématome (petite collection sanguine), se résorbant sous quelques jours et pouvant nécessiter la pose d’un pansement alcoolisé. La dose utilisée pour l’anesthésie locale (Xylocaïne 1% sans adrénaline) est non toxique et très rarement allergisant (allergie aux aminoamides)

***DEXA :*** Il est sans danger et apporte une radiation de l’ordre de 70-140 kV (moins qu’une radiographie pulmonaire). L’examen dure une dizaine de minutes.

- Contraintes de l’essai.

La réglementation en matière d’essai clinique chez l’homme interdit au sujet se prêtant à la recherche de participer à d’autres expérimentations simultanément. Si le sujet accepte de participer à cette étude, il ne devra participer à aucun autre essai clinique pendant la même période

# Protection des personnes

## Justification éthique du protocole

Il s’agit d’un essai clinique d’efficacité, monocentrique, national, contrôlé, randomisé, en double aveugle contre placebo.

## Dispositions éthiques et réglementaires

La recherche sera menée dans le respect de la réglementation française en vigueur, notamment des dispositions relatives à la recherche biomédicale du Code de la Santé Publique, article L 1121-1 et suivants, des lois de Bioéthique, de la loi Informatique, Fichiers et Libertés (et notamment de la méthodologie de référence MR-001 relative aux recherches biomédicales – annexe 6, de la Déclaration d’Helsinki (annexe9), ainsi que du présent protocole.

L’investigateur s’engage à mener la recherche conformément à ces dispositions éthiques et réglementaires. Il est conscient que tous les documents ainsi que toutes les données relatives à la recherche pourront faire l’objet d’audits et d’inspections réalisées dans le respect du secret professionnel et sans que puisse être opposé le secret médical. L’investigateur reconnaît que les résultats de la recherche sont la propriété du laboratoire Pileje, promoteur de la recherche.

## Comité de Protection des Personnes (CPP)

Avant la mise en œuvre de la recherche, le promoteur soumettra le projet à l’avis du CPP Ile de France VI et lui fournira pour cela tous les renseignements nécessaires : protocole de recherche, formulaire d’information (annexe 1) et de consentement (annexe2) et tout autre document pertinent devant être présenté au Comité.

L’essai ne pourra débuter que lorsque le laboratoire Pileje aura été informé de l’avis favorable sans réserve délivré par le CPP à propos du protocole soumis. Cet avis comportera le titre et le numéro du protocole attribué par le promoteur, les documents examinés, ainsi que la date de son examen et la liste des membres du CPP y ayant participé.

Le promoteur informera le CPP de toutes les modifications substantielles du protocole et de tous les évènements indésirables graves ou inattendus et faits nouveaux apparaissant au cours de la recherche et qui affecteraient vraisemblablement la sécurité des personnes s’y prêtant, conformément à la procédure du promoteur décrite dans le présent protocole.

## AFSSAPS

Préalablement à la mise en place d’un essai clinique ne portant pas sur un produit de santé, le demandeur doit :

1. Obtenir un numéro d’enregistrement national de la recherche auprès de l’Afssaps (numéro ID-RCB) ;

2. Transmettre un dossier de demande d’autorisation d'essai clinique (AEC) à l’Afssaps.

L’évaluation du dossier par l’Afssaps comporte :

- l'examen de sa complétude (recevabilité technico-réglementaire),

- son évaluation technique

Les recherches biomédicales ne portant pas sur un produit de santé mentionné à l’article L. 5311-1 du CSP sont soumises à un régime d’autorisation implicite : pour ces recherches, à l’expiration du délai d’évaluation imparti à l’Afssaps (60 jours), le silence gardé par celle-ci vaut autorisation.

Lorsque l’Afssaps requiert des informations complémentaires, et si le demandeur les lui transmet dans le délai qui lui est imparti, l’essai est autorisé de manière implicite si l’Afssaps ne lui a pas notifié sa décision avant l’expiration du délai d’évaluation de 60 jours. Dans ce cas, le promoteur peut solliciter auprès de l’Afssaps, à expiration du délai légal de 60 jours, la délivrance d’une attestation confirmant que l’essai visé par la demande a été autorisé par l’Afssaps.

Selon les dispositions de l’article L. 1123-9 du CSP, après le commencement de la recherche, toute modification substantielle de celle-ci à l'initiative du promoteur doit obtenir, préalablement à sa mise en oeuvre, un avis favorable du comité de protection des personnes (CCP) et une autorisation de l'autorité compétente (autorisation de modification substantielle ou AMS)

Au cours de l’évaluation de la demande d’autorisation de la recherche ou d’avis sur celle-ci, l'Afssaps et le CPP s'informent mutuellement des demandes d'informations complémentaires, des objections à la mise en oeuvre de la recherche, et des demandes de modifications que chacun formule auprès du demandeur. A cette fin, l’Afssaps et le CPP se transmettent mutuellement une copie des courriers qu’ils adressent au demandeur.

Selon les dispositions de l’article L. 1123-9 du CSP, après le commencement de la recherche, toute modification substantielle de celle-ci à l'initiative du promoteur doit obtenir, préalablement à sa mise en oeuvre, un avis favorable du comité de protection des personnes (CCP) et une autorisation de l'autorité compétente (autorisation de modification substantielle ou AMS). Les modifications non substantielles sont celles qui n’ont pas d’impact significatif sur quelque aspect de la recherche que ce soit. Ces modifications ne sont pas à soumettre à l’Afssaps, ni pour autorisation, ni pour information.

Le promoteur doit déclarer à l'Afssaps ainsi qu'au CPP :

De façon immédiate

- les **suspicions d’effets indésirables graves inattendus** (EIGI) survenus en France dans l’essai concerné

- tout **fait nouveau** ou toute donnée nouvelle susceptible de porter atteinte à la sécurité des personnes qui se prêtent à la recherche ou d’avoir un impact sur la conduite de l’essai

De façon périodique

- une **déclaration semestrielle** comprenant :

- une liste des suspicions d’EIGI survenus dans les autres essais conduits en France avec le même produit, acte ou méthode ainsi que ceux survenus hors du territoire national dans l’essai concerné ;

- une synthèse concise préparée par le promoteur mettant en exergue les principaux problèmes de sécurité soulevés.

- le **rapport annuel de sécurité**.

En outre, le promoteur communique :

- au CPP concerné : tous les renseignements complémentaires demandés par celui-ci sur les cas notifiés de décès

- à l'Afssaps : les faits nouveaux portés à sa connaissance après la fin de la recherche.

## Assurance et financement

Le laboratoire Pileje, en tant que promoteur, a souscrit pour toute la durée de l’essai un contrat d’assurance en responsabilité civile auprès de la compagnie HDI-Gerling Industries, dont l’adresse est 111, rue de Longchamp, 75115 Paris, sous le numéro (1680) 90712, conformément aux dispositions légales et réglementaires françaises sur les recherches biomédicales (attestation d’assurance relative au présent protocole en annexe 3 de ce dernier).

L’attestation d’assurance relative au présent protocole en constitue l’annexe 3.

Le financement de la recherche est assuré par le laboratoire Pileje.

## Modalités de recueil du consentement des personnes

Le consentement éclairé écrit de toutes les personnes qui se prêtent à la recherche biomédicale doit être obtenu par l’investigateur, inscrit à l’ordre des médecins, déclaré en tant qu’investigateur auprès du promoteur, avant tout acte pratiqué dans le cadre du protocole de la recherche et quel que soit cet acte, conformément au Code de la Santé Publique, article L1122-1.

L’information sera donnée oralement et par écrit dans la première partie du formulaire d’information et de consentement (annexes 1 et 2). Le formulaire sera paraphé en bas de chaque page afin que la personne se prêtant à la recherche atteste qu’elle a bien reçu cette information. L’information est rédigée dans un langage clair, parfaitement compréhensible pour la personne. Elle doit contenir l’ensemble des éléments dont doit être informée la personne, conformément au Code de la Santé Publique, article L 1122-1.

Le consentement de participation de la personne à la recherche est recueilli par écrit sur la seconde partie du formulaire d’information et de consentement. Cette seconde partie doit être rédigée dans un langage clair, parfaitement compréhensible pour la personne qui se prête à la recherche. Elle doit contenir l’ensemble des éléments auxquels la personne consent. Le consentement est donné par apposition de la signature et inscription, de la main de la personne qui se prête à la recherche, du prénom, du nom et de la date de consentement.

Par ailleurs, l’investigateur qui recueille le consentement date et signe le formulaire à l’emplacement qui lui est réservé et s’assure 1) que le formulaire est conforme et qu’aucune mention ou date n’est manquante, 2) qu’un exemplaire original de ce document est remis au patient. Il conserve un second exemplaire original dans un lieu sécurisé et dont l’accès est contrôlé et 3) place une copie de son exemplaire original dans une enveloppe sécurisée à l’attention du promoteur ; ce troisième exemplaire ne devra pas être ouvert par le promoteur, mais conservé avec le dossier permanent de l’étude.

L’investigateur devra s’assurer que la personne qui se prête à la recherche aura eu le temps de prendre sa décision librement et aura pu lire et comprendre le formulaire d’information et de consentement.

Le formulaire d’information et de consentement est un document qui aura été approuvé préalablement à la mise en œuvre de la recherche par le CPP, à l’occasion de l’examen du protocole.

## Indemnités

Elle est de 500 Euros ; cette somme sera versée à la fin de l’étude, soit après 24 semaines de participation. Si le sujet est amené à arrêter l’étude avant la fin, aucune indemnisation au prorata ne lui sera versée.

# Objectifs

## Objectif principal

Evaluer, l’influence d’une supplémentation de 4 mois avec Glycabiane sur la glycémie à jeun de sujets prédiabétiques en surpoids ou obèses.

## Objectifs secondaires

Etudier l’influence d’une supplémentation de 4 mois avec Glycabiane sur :

- le taux d’hémoglobine glyquée (HbA1c)
- le profil lipidique (Cholestérol total, LDL-c, HDL-c, TG)
- Les marqueurs de l’inflammation : les taux circulants de l’IL6 et la CRPus
- L’insulinoresistance (HOMA, QUICKI révisé)

HOMA IR : I_0_/(22.5Xe^-ln(gly^_0_))

QUICKI révisé: 1/[Log(I_0_)+ Log(gly_0_) + Log(FFA_0_)]

● Marqueurs de l’adiposité : poids, IMC (poids/taille²), tour de taille, tour de hanche, composition corporelle (dont pourcentage de masse grasse et de masse maigre), taille adipocytaire

- le statut inflammatoire du tissu adipeux (sécrétion d’IL-6 et d’adiponectine )
- la sensibilité à l’insuline du tissu adipeux par la mesure de l’adiponectine sécrétée et par l’étude de la phosphorylation d’Akt

Evaluer la tolérance d’une supplémentation de 4 mois avec Glycabiane

Evaluer les apports alimentaires avant et après la complémentation

# Plan expérimental

## Type d’essai

Il s’agit d’un essai clinique d’efficacité, monocentrique, national, randomisé en double aveugle contre placebo et mené en groupes parallèles*.*

Etude longitudinale avec suivi planifié de 5 visites : J-14 à -1 J0, J60±7, J120±7 et J180±7.

## Catégorie de recherche :

Recherche biomédicale ne portant pas sur un produit mentionné à l’article L. 5311-1 du code de la santé publique.

Recherche biomédicale portant sur un complément alimentaire.

## Exposé du plan expérimental

Une population de 60 sujets prédiabétiques en surpoids ou obèses sera sélectionnée après un examen de pré inclusion.

Après l'inclusion, les sujets seront randomisés en 2 groupes pour suivre une complémentation de 4 mois soit de Glycabiane soit de Placebo (2 gélules par jour). Trois visites de suivis seront également programmées : une après 2 mois de complémentation, une autre à la fin de la période de complémentation à 4 mois, et une dernière 2 mois après l’arrêt de la complémentation à 6 mois.

Schéma général

| Visites | V1  Inclusion | V2  J0-Tr | V3  2M | V4  4M  fin de tr. | V5  6M  follow-up |
| --- | --- | --- | --- | --- | --- |
| Jours | -14 à -1 | 0 | 60±7 | 120±7 | 180±7 |
| Critères inclusion/non-inclusion | X | X |  |  |  |
| Traitement Exp. |  | X | x | x |  |
| Information/  Signature consentement | X | ~~X~~ |  |  |  |
| TA | X | X | X | X | X |
| Poids (kg) | X | X | x | X | x |
| Périmètre abdominal | X | X | x | x | x |
| **Bilan somatique et nutritionnel**  **-** Mesures anthropométriques  - Mésure de la composition corporelle (DEXA)  - Carnet nutritionnel (voir annexe 7) | X  X | x  x  x | x  x | x  x  x | x  x |
| **Bilan de sélection :**  -Bilan hépatique :  ASAT, ALAT, gammaGT  - Bilan rénal: Ionno Elargi  - NFS + plaquettes  - Sérologie : HIV, Hépatite C  - Test de grossesse : Beta-hCG | X |  |  |  |  |
| **Bilan métabolique**  - Glycémie  - Insulinémie,  -Bilan lipidique (Cholesterol, LDL-, HDL- Cholesterol, TG)  - AGL  - % HbA1c | x  X | x  x  x  X  x |  | x  x  x  X  x | x  x  x  X  x |
| **Bilan inflammatoire**  - CRPus, IL6 |  | X |  | x | x |
| **Signaux d’adiposité**  - Leptine, adiponectine  - Ponction à l’aiguille du tissu adipeux abdominal sous-cutané - Taille adipocytaire:  - Etude sur les adipocytes en culture (in vitro) |  | X  x |  | X  x | X |
| **Questionnaires de qualité de vie (TFEQ, activité physique)**  **Questionnaire alimentaire (carnet)** | X | X  X | X | X  X | X  X |

# Critères d’évaluation

## Critère principal

Quantification de l’amélioration de la glycémie à jeun.

## Critères secondaires :

Evaluation des changements du pourcentage d’HbA1c, du profil lipidique, de l’insulinémie, de la leptinémie, des caractéristiques anthropométriques, de la taille des adipocytes à la fin de la complémentation, du statut inflammatoire et de la sensibilité à l’insuline du tissu adipeux

Evaluation de la qualité de vie

# Sélection des personnes

## Population étudiée participante:

### Durée de la participation des personnes à la recherche :

La durée totale de participation de chaque sujet sera de 6 mois consécutifs.

### Description des personnes participantes :

Critères d’inclusion

- Hommes et femmes âgés de 25 à 65 ans

- Répondant aux critères suivants :

- 1,0g/L ≤glycémie à jeun<1,26g/L

- Surpoids ou obésité (IMC≥25)

- Ne souhaitant pas modifier leurs habitudes alimentaires et d’activité physique

- Bilans rénaux et hépatiques normaux

- Affilié à un régime de sécurité sociale française,

- Suffisamment coopérant pour se conformer aux impératifs de l'étude,

- Immunocompétent (pas de VIH ni d’hépatite C connu),

- Utilisation d’une méthode de contraception efficace chez les femmes en âge de procréer

Critères d’exclusion

- maladie thyroïdienne
- hypogonadisme

- antécédent de maladie musculo-squelettique, auto-immune ou neurologique

- hépatite C, HIV

- traitement médicamenteux contre une maladie thyroïdienne hypoglycémie, ou anti-coagulant

- prise médicamenteuse pouvant influer sur :

- la prise/perte de poids

- le contrôle glycémique

- l’insulinémie

- femme enceinte ou allaitante

- perte de poids >5% au cours des 6 derniers mois

- consommation de complément alimentaire à base de cannelle ou de chrome

- sujet faisant l’objet d’une mesure de sauvegarde de justice
- sujet ayant perçu 4500€ d’indemnités compensatrices au cours des 12 derniers mois. Cette information est à vérifier par l’investigateur, les sujets étant indemnisés dans la recherche
- antécédent d’allergie à l’un des composants du produit expérimental
- contre-indication à l’utilisation de xylocaïne comme anesthésiant local

### Nombre de personnes à inclure :

Calcul d’effectif : 25 sujets par groupe sont nécessaires pour montrer une variation de la glycémie à jeun d'au moins 10% et un écart type de 0,13g/L (moyenne des écarts-type observés dans les études cliniques publiées sur le diabète de type 2 et la cannelle) en fixant le risque α à 0,05 et le risque β à 0,10. 60 sujets seront sélectionnés dans l’étude selon les critères d’inclusion et de non inclusion, pour assurer l’inclusion et le suivi du protocole dans sa globalité de 50 sujets.

## Recrutement et sortie de l’essai

### Recrutement

Les sujets seront recrutés par différentes voies de campagne de communication affichée:

- dans les cabinets médicaux de médecins exerçant à Paris ou en Ile-de-France, via des affichettes éditées par le CRNH (voir annexe 8)
- Les médecins qui le souhaitent pourront également être amenés à proposer l’étude en cours de consultation. Leur contribution à l’étude se limitera à la pré-sélection de patients prédiabétiques en surpoids et ou obèses, sur la base du volontariat de sujets présentant une glycémie anormale (1,0g/L≥glycémie à jeun>1,26g/L), répondant aux critères de sélection, ayant lu la note d’information et acceptant de se rendre au service de Nutrition du GH Pitié Salpêtrière pour valider ou non leur inclusion dans l’étude.
- dans des laboratoires d’analyses biomédicales, des pharmacies, des centres de dépistage du diabète via les affichettes éditées par le CRNH (voir annexe 8)
- dans le journal « Métro », édition de Paris (voir copie de l’affiche en annexe 8)
- via « Volterys », « site de rencontre » pour volontaires et chercheurs

Les sujets seront sélectionnés après un interrogatoire médical, un examen clinique complet effectué par le médecin investigateur ou co-investigateur et un bilan biologique.

### Sortie du protocole

**Critères d’arrêt ou de sortie prématurée de l’étude :**

- effet indésirable grave, c’est-à-dire « tout événement ou effet indésirable qui entraîne la mort, met en danger la vie de la personne qui se prête à la recherche, nécessite une hospitalisation ou la prolongation de l’hospitalisation, provoque une incapacité ou un handicap important ou durable, ou bien se traduit par une anomalie ou une malformation congénitale, quelle que soit la dose administrée » (ICH E6 : BPC, 1.50).
- non respect du protocole.

**Décision délibérée d’une personne :**

La sortie du protocole est possible à tout moment si elle est demandée par le participant. Plus particulièrement, si les participants ne souhaitent pas continuer les tests, il leur suffira de le signaler à l’examinateur.

**Décision imposée** :

Les participants ne pouvant pas réaliser les tâches relatives à la recherche seront exclus du protocole.

### Période d’exclusion et participation à une autre recherche

La période d’exclusion définie dans le cadre de cette étude est de 3 mois, période pendant laquelle le sujet ne peut participer à un autre protocole de recherche clinique.

Cette exclusion se justifie pour éviter tout risque d’interférence dans l’interprétation des résultats.

L’investigateur s’assure que les personnes susceptibles de se prêter à la recherche biomédicale ne participent pas déjà à une recherche qui pourrait rendre impossible leur inclusion dans la recherche proposée. Il s’assure également qu’elles n’ont pas participé à une recherche pour laquelle actuellement une période d’exclusion est requise, notamment à l’aide du Fichier National des personnes qui se prêtent à des recherches biomédicales.

# Réalisation pratique du protocole

## Description détaillée des actes pratiqués sur les personnes :

Visite dite de sélection/inclusion J-14 à J-1

- Information de la personne se prêtant à la recherche et recueil du consentement
- Visite médicale et bilan nutritionnel
- Bilan biologique d’inclusion spécifique comportant :
  - une numération/formule sanguine (NFS-) et vitesse de sédimentation (VS),
  - une sérologie HIV et HCV,
  - un bilan rénal (ionogramme sanguin (sodium plasmatique, potassium plasmatique, calcium, réserve alcaline, phosphorémie), créatinine, urée)
  - un bilan hépatique (ALAT, ASAT, Gamma GT)
  - un bilan lipidique (Cholestérol total, HDL et LDL cholestérol, triglycérides)
  - une glycémie
  - un test de grossesse si volontaire non ménopausée et sans contraception efficace
  - carnet nutritionnel (voir annexe 7)

Visite V2-J0 -randomisation:

- Vérification des critères d’inclusion
- Examen clinique, bilan somatique et nutritionnel (tension artérielle, poids, périmètre abdominal, mesures anthropométriques incluant la composition corporelle, carnet nutritionnel)
- Bilan biologique après une nuit de jeûne de 8h00 comprenant glycémie, insulinémie, cholestérol, LDL-, HDL-, triglycérides, CRPus, IL6, leptinémie, HbA1c
- Aspiration à l’aiguille du tissu adipeux sous-cutané
- Remise du carnet nutritionnel et du carnet de bord pour la prochaine visite
- Remise et récupération des questionnaires de qualité de vie TFEQ*, questionnaire d’activité physique (voir annexe 6) et carnet nutritionnel (annexe 7)
- Remise du traitement (Glycabiane)
- Mesure de la composition corporelle (DEXA))
- Prise de rendez-vous pour la visite 3 (J60±7)

*Le Three Factor Eating Questionnaire (TFEQ) est un auto questionnaire qui permet d’étudier trois dimensions du comportement alimentaire : la faim, la restriction et la désinhibition. Celle-ci est définie par la survenue d’accès incontrôlés d’hyperphagie, qui sont le plus souvent secondaires à la levée d’une restriction alimentaire.

Visite 3 (V3 au 2^ème^ mois) J 60 ± 7

- Examen clinique, bilan somatique et nutritionnel (tension artérielle, poids, périmètre abdominal, mesures anthropométriques, carnet nutritionnel)
- Récupération des boites et blisters vides des produits consommés
- Prise de rendez-vous pour la visite de fin de complémentation (J120±7)

Visite 4 (V4 au 4^ème^ mois) de fin de complémentation J 120 ± 7

- Récupération des boites et blisters vides des produits consommés et des produits restants non consommés
- Remise du carnet nutritionnel et du carnet de bord pour la prochaine visite
- Remise et récupération des questionnaires de qualité de vie TFEQ,questionnaire d’activité physique et carnet nutritionnel (annexe 7)
- Examen clinique, bilan somatique et nutritionnel (tension artérielle, poids, périmètre abdominal, mesures anthropométriques incluant la composition corporelle, carnet nutritionnel)
- Bilan biologique après une nuit de jeûne, à 8h00 comprenant glycémie, insulinémie, cholestérol, LDL-, HDL-, triglycérides, CRPus, IL6, leptinémie, HbA1c
- Aspiration à l’aiguille du tissu adipeux sous-cutané
- Mesure de la composition corporelle (DEXA)
- Prise de rendez-vous pour la visite de « follow-up » (J194± 7)

Visite 5 (V5-au 6^ième^ mois) de « folow-up »  J 194 ± 7

- Examen clinique, bilan somatique et nutritionnel (tension artérielle, poids, périmètre abdominal, mesures anthropométriques, carnet nutritionnel)
- Bilan biologique après une nuit de jeûne, à 8h00 comprenant glycémie, insulinémie, cholestérol, LDL-, HDL-, triglycérides, CRPus, leptinémie, HbA1c
- Recueil du carnet nutritionnel et du carnet de bord
- Remise et récupération des questionnaires de qualité de vie
- Fin de l’étude

**Bilan nutritionnel et inflammatoire**

Nous allons caractériser le profil lipidique et le contrôle glycémique des sujets: avant et après chaque période. Au début et à la fin de chaque période, après une nuit de jeûne à 8h00 un prélèvement sanguin à jeun sera pratiqué (pour les dosages de la glycémie, l'insulinémie, les choléstérols total, LDL, HDL, les triglycérides, la leptine, adiponectine). Les facteurs inflammatoires : CRPus et IL6 seront mesurés (ELISA).

La masse grasse et sa répartition seront mesurées à l'aide de l'absorptiométrie biénergétique (DEXA). Un prélèvement à l’aiguille de tissu adipeux sous-cutané abdominal sera pratiquée par aspiration sous vide après une anesthésie locale à la xylocaïne 10 % sans isoprénaline.

Nous allons caractériser le profil alimentaire tout au long de l’étude au moyen d’un carnet nutritionnel, de type « semainier » sur 3 jours.

**Prélèvement du tissu adipeux :**

Les prélèvements de tissu adipeux sous cutané abdominal (environ 500 mg) seront réalisés à l’aiguille au moment de l’inclusion et au décours du suivi. Ce prélèvement, réalisé par un médecin investigateur, est réalisé en routine dans le service de nutrition de l’Hôpital Pitié Salpetrière. Après une anesthésie locale (Xylocaïne 1% sans adrénaline), une aspiration de tissu adipeux à l’aiguille fine est réalisée. Une partie du prélèvement permettra de mesurer la taille adipocytaire avant et après complémentation. Une autre servira à évaluer :

1) le statut inflammatoire du tissu adipeux par dosage Elisa de la cytokine IL-6 et de l’adipokine, adiponectine.

2) la sensibilité à l’insuline par étude de la phosphorylation d’Akt par Western Blot et par la sécrétion d’adiponectine.

Ces analyses 1) et 2) seront sous-traitées à la société AdipoPhYt.

**Traitement des échantillons biologiques**

L’ensemble des sérums destinés aux dosages des signaux d’adiposité et des facteurs inflammatoires sera analysé à l’Unité l’INSERM U872 (Eq 7). La sérothèque sera conservée par l’Unité Inserm U872 (Eq. 7). Pour chaque prélèvement de tissu adipeux (sous cutané) la taille cellulaire sera évaluée avant et après traitement. Les études expérimentales du tissu adipeux in vitro en culture (statut inflammatoire et sensibilité à insuline) seront réalisées par la société AdipoPhYt.

## Description de l’organisation logistique générale de l’essai

Les investigateurs du laboratoire INSERM U872 (Eq 7), Pôle d’Endocrinologie, CRNH-Ile de France à la Pitié Salpêtrière auront la responsabilité du recrutement (sous-traité à AdipoPhYt) et de la sélection des patients, de la dispensation des produits, de la mesure et de l’enregistrement sur le cahier d’observation des critères d’évaluation.

Le laboratoire PiLeJe fournira les produits (placebo et actif). Un attaché de recherche clinique (ARC) promoteur réalisera le suivi de l’étude afin de vérifier les données sources et la conduite de l’étude selon les bonnes pratiques cliniques. Le personnel d’AdipoPhYt contacte le sujet la veille de l’expérience pour lui rappeler le rendez vous et l’obligation de respecter un jeûne de 8h.

Le laboratoire INSERM U872 (Eq 7) est en charge de la rédaction des chapitres statistiques du protocole, du data-management, des analyses statistiques et de la rédaction du rapport statistique.

Les produits et placebo seront mis en aveugle par le promoteur (Laboratoire Pileje)

## Calendrier prévisionnel de l’essai (Flow-Chart)

- Durée de la période d'inclusion : 6 mois

Dans le cas de sorties du protocole pour raison médicale intercurrente, le sujet sortant ne sera pas remplacé

- Durée totale de l'étude : 24 mois
- La durée totale de participation de chaque sujet sera de 194 jours consécutifs.

# Produit à l’étude

## Description du produit à l’étude

### Glycabiane

- - Spécialité : complément alimentaire
  - Substances actives : OPC-A de cannelle, guanylor chrome et carnosine
  - Galénique : gélules
  - Composition : une gélule de 595 mg de Glycabiane contient :

| **Ingrédients** | **Quantité / gélule** |
| --- | --- |
| Extrait de cannelle | 228,000 mg |
| Carbonate de calcium | 136,750 mg |
| L-carnosine | 100,000 mg |
| Gélules HPMC transparente (t. 0) | 95,000 mg |
| SIPERNAT | 16,000 mg |
| Talc | 7,000 mg |
| Stéarate de magnésium | 6,000 mg |
| Silice hydratée | 5,000 mg |
| Guanylor chrome | 1,250 mg |
|  |  |
| **TOTAL** | 595mg |

En bleu, les substances actives

- Mode d’administration : voie orale
- Posologie : 2 gélules par jour à prendre pendant le repas du midi pendant 4 mois
- Conditionnement : blisters de 20 gélules
- Etiquetage : conforme à l’arrêté du 24 mai 2006 (JO du 30 mai 2006 – texte n°41) fixant le contenu de l’étiquetage des médicaments expérimentaux.

### Placebo

- - Spécialité : placebo
  - Galénique : gélules
  - Composition : une gélule de 595 mg de Placebo contient :

| Ingrédients | Quantité / gélule |
| --- | --- |
| Carbonate de calcium | 136,750 mg |
| Gélules HPMC transparente (t. 0) | 95,000 mg |
| Talc | 7,000 mg |
| Stéarate de magnésium | 6,000 mg |
| Silice hydratée | 5,000 mg |
| Dioxyde de silicium | 16,000 mg |
| Cellulose microcristalline | 230,25 mg |
|  |  |
| TOTAL | **496 mg** |

- Mode d’administration : voie orale
- Posologie : 2 gélules par jour à prendre pendant le repas du midi pendant 4 mois
- Conditionnement : blisters de 20 gélules
- Etiquetage : conforme à l’arrêté du 24 mai 2006 (JO du 30 mai 2006 – texte n°41) fixant le contenu de l’étiquetage des médicaments expérimentaux.

## Délivrance et compliance

Le produit à l’étude et le placebo seront directement livrés au laboratoire INSERM U872 (Eq7).

La température de stockage des produits doit être inférieure à 25°C.

Les produits non utilisés seront récupérés et collectés par le promoteur qui s’engage à éliminer ces déchets conformément à la réglementation en vigueur.

Des documents traçant l’élimination de ces déchets (bordereaux de suivi de déchets, bons de collecte, etc…) seront émis par le promoteur.

La compliance sera évaluée grâce à 2 paramètres :

- l’ « interrogatoire » du patient ;
- le retour des produits non utilisés ;

Aucun médicament ou traitement susceptible d’avoir une action sur le contrôle glycémique des sujets se prêtant à la recherche ne sera autorisé pendant l’essai

# Dispositifs médicaux

- Mesure de la composition corporelle

Le poids corporel sera mesuré sur une balance en sous-vêtements, à ± 0,1 kg. La taille sera mesurée pieds-nus à ±0,1 cm. Les périmètres seront mesurés avec un centimètre de couturière. Le tour de taille est le plus petit périmètre à hauteur de l’ombilic et le tour de hanches est le plus grand périmètre en regard des têtes fémorales. Ces méthodes sont standardisées au CRNH et réalisées par le même investigateur.

La composition corporelle sera mesurée par absorptiométrie biphotonique (DEXA). Cette méthode est habituellement utilisée pour la mesure de la densité minérale osseuse. Elle repose sur un balayage du corps par un faisceau de rayon X à 2 niveaux d’énergie différents, qui permettent d’individualiser 3 compartiments : la masse minérale, la masse tissulaire active et la masse grasse. Cette méthode peu irradiante (moins qu’une radiographie thoracique classique) est utilisée chez l’adulte comme chez l’enfant et est désormais considérée comme une méthode de référence pour mesurer précisément les compartiments corporels. Pour cet examen, le sujet est allongé sur une table, en sous vêtements. La durée totale de l’étude est d’environ 10 minutes.

- Prélèvements sanguins à jeun

Le matin à jeun (8 heures de jeûne), les volontaires subiront un prélèvement de sang d’environ 40 ml qui sera immédiatement centrifugé, aliquoté et congelé pour l’analyse des substrats et des hormones. Nous allons doser les taux circulants :

- de glucose et d’insuline
- des lipides : cholestérol, triglycérides, acides gras libres et les lipoprotéines de haute et basse densité (HDL et LDL- cholestérol)
- de la leptine et de l’adiponectince
- des facteurs inflammatoires : CRPus,et IL6.

Environ 7 ml de sang seront recueillis pour obtenir du plasma ou du sérum qui seront conservés afin d’effectuer des dosages complémentaires au regard des résultats des dosages précédents.

L’ensemble des sérums destinés aux dosages des signaux d’adiposité et des facteurs inflammatoires sera analysé dans l’Unité l’INSERM U872 (Eq 7). La sérothèque sera conservée par l’Unité Inserm U872 (Eq. 7) Le volume total de sang, prélevé en quatre temps de visite, sera donc d’environ 160 mL pour la totalité de l’étude. Les prélèvements seront conservés à -80°C jusqu’à la fin de l’étude, puis ils seront détruits.

- Prélèvement de tissu adipeux péri-ombilical

Un prélèvement par aspiration à l’aiguille de tissu adipeux d’environ 500 mg sera réalisé sur les volontaires. Ce prélèvement, réalisé par un médecin investigateur, est réalisé en routine dans le service de nutrition de l’Hôpital Pitié Salpetrière pour mesurer les tailles cellulaires. Après une anesthésie locale avec 2 ml de xylocaïne 1% sans isoprénaline, le tissu adipeux péri-ombilical sera prélevé par aspiration à l’aiguille fine pour suivre l’évolution de la population et de morphologie cellulaire. Pour chaque prélèvement de tissu adipeux (sous cutané), la taille cellulaire sera évaluée avant et après le traitement.

Les études expérimentales d’exploration du tissu adipeux (statut inflammatoire, sensibilité à l’insuline) in vitro en culture seront réalisées par la société AdipoPhyt.

- Consignes particulières lors de la période expérimentale

Les personnes se prêtant à la recherche ne devront pas modifier leurs habitudes de consommation alimentaires. Enfin, toute forme de traitement médicamenteux initié pendant la période expérimentale devra être rapportée par le volontaire.

# Collection d’Echantillons biologiques d’origine humaine

- Description de la nature des échantillons :

Prélèvements sanguins et de tissu adipeux.

- Description des conditions de prélèvement :

Les prélèvements sanguins seront effectués à J-1 à -14, J0, J 120 et J180 après 8 heures de jeûne, entre 8 heures et 9h30 le matin. Les tubes prélevés seront mis à 4°C dès le prélèvement, centrifugés à 4°C, aliquotés et conservés à – 80°C jusqu’à leur utilisation pour les analyses prévues.

Le prélèvement de tissu adipeux sera réalisé après une anesthésie locale avec 2 ml de xylocaïne 1% sans isoprénaline, le tissu adipeux péri-ombilical sera prélevé par aspiration à l’aiguille fine pour mesurer immédiatement la taille adipocytaire et réaliser l’étude de culture cellulaire in vitro.

Les prélèvements sanguins seront conservés par l’INSERM U872 (Eq7) à -80°C jusqu’à la fin de l’étude (dernières analyses du dernier patient inclus), puis ils seront détruits. Ceux de tissu adipeux péri-ombilical seront conservés à -80°C par ADIPOPHYT jusqu’à la fin de l’étude (dernières analyses du dernier patient inclus), puis ils seront détruits.

# vigilance de l’essai

## DEFINITIONS RELATIVES A LA VIGILANCE

Cf. Article R. 1123-39 1° ; 2° ; 6° ; et 8° du CSP

Evénement indésirable

Toute manifestation nocive survenant chez une personne qui se prête à une recherche biomédicale que cette manifestation soit liée ou non à la recherche ou au produit sur lequel porte cette recherche.

Effet indésirable

Tout événement indésirable dû à la recherche.

Pour chaque événement indésirable, l’investigateur et le promoteur évaluent son lien de causalité avec la recherche. Tout événement indésirable considéré soit par l’investigateur soit par le promoteur comme ayant un lien de causalité scientifiquement raisonnable avec la recherche est qualifié d’effet indésirable. L’expression « lien de causalité scientifiquement raisonnable » signifie généralement qu’il existe une preuve ou un argument permettant de suggérer, sur le plan scientifique, une relation de cause à effet entre la réaction nocive et non désirée observée et la recherche (à savoir la procédure, la méthode, l’acte ou le produit faisant l’objet de la recherche). L’événement indésirable dû à la recherche peut être lié, par exemple, aux procédures, aux méthodes, aux actes pratiqués ou aux produits faisant l’objet de la recherche ou utilisés pour les besoins de la recherche.

Évènement ou effet indésirable grave

Tout évènement ou effet indésirable répond à la définition de "grave" s'il :

- entraîne la mort ;

- met en danger la vie de la personne qui se prête à la recherche ;

- nécessite une hospitalisation ou la prolongation de l’hospitalisation ;

- provoque une incapacité ou un handicap importants ou durables ;

- se traduit par une anomalie ou une malformation congénitale.

Pour chaque événement /effet indésirable, l’investigateur et le promoteur évaluent son lien de gravité.

L’expression «met en danger la vie de la personne» correspond à un événement / effet entraînant une menace vitale immédiate pour la personne qui se prête à la recherche au moment de l’événement indésirable, et non à un événement / effet qui aurait pu éventuellement entraîner le décès s’il avait été plus sévère. Le jugement médical doit être exercé pour déterminer si l’événement ou l’effet indésirable est grave dans d’autres situations. Les effets ou événements indésirables importants, qui ne mettent pas immédiatement la vie en danger ou qui n’aboutissent pas à un décès ou à une hospitalisation, mais peuvent mettre le sujet en danger ou nécessiter une intervention pour éviter d’évoluer vers l’un des critères ci-dessus, doivent aussi être considérés comme graves.

## Rôle des investigateurs

### Evaluation de la gravité/lien de causalité des évènements indésirables

L’investigateur doit évaluer pour chaque événement indésirable recueilli au cours de la recherche :

- sa gravité,

- le lien de causalité entre l’événement indésirable grave et la recherche,

, puis transmet au promoteur le résultat de son évaluation.

Notification au promoteur des événements indésirables graves et non graves

L'investigateur notifiera sans délai à compter du jour où il en a connaissance tous les EIG survenus dans la recherche :

A l’aide du formulaire de déclaration EIG qu’il adressera par fax au par e-mail au promoteur au 01.45.51.12.74 . à l’attention de : Mme Séverine BIEUVELET

Lors de cette notification, l’investigateur transmet au promoteur son évaluation du lien de causalité de l’événement indésirable grave avec la recherche (c’est-à-dire avec la procédure, la méthode, l’acte ou le produit faisant l’objet de la recherche).

L’investigateur fournira également toute information nécessaire à l’évaluation médicale de l’observation au promoteur (compte-rendu d’examens complémentaires, anatomopathologie, radiologie).

Le promoteur pourra demander à l’investigateur des compléments d’informations.

Tous les documents transmis par l’investigateur doivent être anonymisés.

Les EIG survenant après l’essai doivent être signalés au promoteur et suivis jusqu’à leur résolution définitive.

## Rôle du promoteur

Le promoteur devra déclarer toutes les suspicions d'effets indésirables graves liés au produit ou à la recherche au CPP Ile-de-France VI et à la Direction Générale de la Santé (DGS)l’AFSSAPS sans délai et au plus tard dans un délai initial de 7 jours calendaires à compter du jour où il en a eu connaissance pour les effets indésirables graves ayant entraîné le décès ou mis en jeu le pronostic vital, et dans un délai de 15 jours pour les autres effets indésirables graves. Toute déclaration initiale sera complétée d’une déclaration de suivi détaillant les informations complémentaires pertinentes concernant les effets indésirables graves dans un nouveau délai de 8 jours à compter des délais mentionnés ci-dessus.

Le promoteur se prononcera sur la signification des effets indésirables graves qu’il déclare et les conséquences qu’il en tire, notamment en ce qui concerne la conduite de la recherche.

Le promoteur se prononcera également sur l’imputabilité de l’effet indésirable au moyen d’une analyse conjointe avec l’équipe des dispositifs de vigilance PiLeJe.

Le caractère inattendu sera établi par le promoteur à partir des données de nutrivigilance de Pileje concernant le produit à l’étude d’une part et de la littérature scientifique et médicale concernant les ingrédients constitutifs du produit d’autre part. ~~.~~

Le promoteur tiendra des registres détaillés de tous les événements indésirables (EI, EIG, EIGI)

Le promoteur déclarera de façon périodique à l’AFSSAPS ainsi qu’au CPP :

- Semestriellement :
- une liste des suspicions d’EIGI survenus dans les autres essais conduits en France avec le même produit, acte ou méthode
- une synthèse concise mettant en exergue les principaux problèmes de sécurité soulevés.
- Annuellement :
- Un rapport annuel de sécurité tenant compte de toutes les informations de sécurités disponibles.

Le promoteur tient des registres détaillés de tous les évènements indésirables qui lui sont notifiés par les investigateurs.

## Suivi des patients en cas de survenue d’un évènement indésirable grave

Le suivi des patients ayant eu un effet indésirable grave sera assuré par le médecin responsable de l’étude.

## Comité de surveillance indépendant

Il n’y a pas de comité de surveillance car cela n’est pas nécessaire compte tenu du produit : risque quasi nul car c’est une association d’ingrédients alimentaires connus et déjà utilisés dans d’autres formulations de compléments alimentaires commercialisés par le groupe Pileje (OligobianeCr (mis sur le marché début 2001) et Multibiane Age Protect (mis sur le marché en mars 2008) pour le Guanylor®-Cr et Glycabiane (mis sur le marché en mars 2008) pour l’association Glycabiane et la nutrivigilance est vierge de tout effet indésirable.

# Recueil et traitement des données

## Recueil des données

Les données sous forme de questionnaires seront recueillies dans un cahier d’observation par les investigateurs mandatés dans cette étude. Les données concernant les prélèvements biologiques des patients seront insérées dans le dossier médical du sujet se prêtant à la recherche. Une copie anonymisée sera insérée dans le CRF dans la pochette plastique prévue à cet effet.

## Description du circuit des données avant la saisie informatique

Les données sont stockées en armoires fermées à clé sur place.

## Recueil des données

Les données seront recueillies dans le respect du secret de l'identité des personnes participant à l'étude et de toute information à caractère personnel ou médical les concernant. L'anonymat des patients sera assuré par la mention au maximum des 3 premières lettres du nom et des 2  premières lettres du prénom du patient sur tous les documents nécessaires à la recherche, ou par effacement par les moyens appropriés (blanc, correcteur…) des données nominatives sur les copies des documents sources, destinés à la documentation de la recherche.

Les données cliniques seront notées ou agrafées dans les cahiers d'observation à partir des données de base après anonymisation.

Le recueil des données sera effectué par l’intermédiaire d’un cahier d’observation spécifique à l’étude. Chaque dossier sera complété au fur et à mesure du recueil des informations. En cas de données manquantes, celles-ci seront indiquées par le sigle “N.D.” (non disponible). En cas de données non applicables au sujet inclus dans l’étude, les emplacements des données seront annotés du sigle “N.A.” (non applicable). Si des corrections doivent être apportées au cahier d’observation, la donnée erronée devra être barrée d’un trait (sans être masquée), la nouvelle donnée doit être écrite à côté avec le paraphe de l’investigateur et la date à laquelle la correction aura été effectuée. Les investigateurs signeront les pages du cahier correspondant à leurs examens.

Les sujets auront un droit d’accès aux données et documents sources auprès des investigateurs de l’étude. Les données seront archivées confidentiellement pour une durée de 15 ans à l’issue de la recherche. Les volontaires sains, conformément à la législation, seront informés également des résultats globaux de la recherche.

Les données sont stockées en armoires fermées à clé sur place.

## Saisie informatique des données

Les données sont numérisées en saisie simple à double lecture. Cela sera fait sous la responsabilité de Dr Salwa Rizkalla, médecin coinvestigateur de l’étude qui valide les data après chaque saisie. La saisie est réalisée dans un classeur Excel où chaque feuille correspond à un type de paramètre. Une feuille récapitulative permet de reporter l'ensemble des données sur une seule feuille, ce qui permet l'export vers le logiciel d'exploitation statistique (SAS).

- La traçabilité sera assurée par un tableau, ci-joint. Chaque modification sera notée, datée en citant la raison et l’auteur de cette modification.

− Les données seront conservées dans un ordinateur dans le bureau du DR S Rizkalla, cet ordinateur est sur le réseau sécurisé de l’hôpital Pité Salpêtrière, hôpital de l’APHP, avec un ID spécifique et un mot de passe.

− Une sauvegarde hebdomadaire sur une clé dédiée à l’étude ainsi que la conservation des documents « papier » sera également réalisé au sein du laboratoire. L’ensemble de ces données sera conservé dans une armoire fermée à clé.

− De même, les procédures, les coordonnées des médecins participant à la recherche et les comptes-rendus des réunions de pilotage et de suivi de l’étude seront conservées dans une pièce et meuble fermés à clé. L’accès sera réglementé et restreint à une liste de participants établie par le responsable scientifique.

- Enfin toutes ces données seront transférées sur un serveur de sauvegarde de l’Unité U872 (équipe 7) qui est sécurisé selon les règles en vigueur à l'Inserm.

La confidentialité relative à chaque volontaire participant à l’étude est garantie. L’accès aux données cliniques et sources sera direct en cas d’audits commandés par le promoteur ou inspections menées par les autorités administratives compétentes.

# Analyse statistique des données

## Nombre de sujets nécessaires

D’après la littérature on peut s’attendre à une diminution de la glycémie à jeun de 10% dans le goupe Glycabiane comparé au groupe Contrôle, après 4 mois de complémentation. L’hypothèse statistique est une différence du critère principal de jugement (Glycémie à jeun) de 10% entre les sujets recevant le placebo et ceux recevant Glycabiane®. Le nombre de sujets à inclure dans cet essai, avec un risque alpha de 5% et une puissance de 90%, est donc de 25 par groupe*,* soit au total 50 sujets. 60 sujets seront sélectionnés dans l’étude selon les critères d’inclusion et de non inclusion, pour assurer l’inclusion et le suivi du protocole dans sa globalité de 50 patients.

**Considérations statistiques**

Toutes les données seront listées par groupe et par sujet.

Des statistiques descriptives seront réalisées suivant la nature du critère analysé :

- Variable quantitative : taille de l’échantillon, moyenne, écart-type, minimum et maximum des données brutes et si nécessaire des variations par rapport à la baseline, ainsi que des pourcentages de variation.
- Variable qualitative : taille de l’échantillon, fréquence absolue (N) et relative (%) par classe.

Ces statistiques descriptives seront réalisées par groupe.

Des graphiques seront fournis suivant la nature des variables :

Variable quantitative : Représentation par groupe de la moyenne +/- SEM au cours des visites.

Variable qualitative : Représentation par groupe des pourcentages par classe

Les variations des paramètres cliniques et biologiques seront analysées pour chaque individu avant et lors du suivi cinétique. Des tests cinétiques de type Manova et Anova seront utilisés. Nous mettrons en relation les paramètres cliniques, biologiques, les données morphologiques et les données d’expression par des analyses de régression logistique. L’INSERM U872 (Eq7) a acquis lors des années précédentes les compétences pour l'analyse de ces données (utilisation de logiciel type JMP (issu de SAS). Pour les traits quantitatifs, des analyses de variance en mesure répétée seront utilisées (Anova, Manova, modèle de régression logistique). Les tests statistiques seront réalisés avec un risque alphade conclure à une différence à tort, fixé à 5%.

L’analyse statistique sera réalisée par l’INSERM U872 (Eq7).

Description de l’échantillon

La description des sujets non inclus, inclus ayant terminé l’étude et inclus sortis prématurément sera présentée à l’aide d’un tableau de fréquence absolue (N) et relative (%).

Les raisons des sorties prématurés seront présentées dans un listing.

La description des sujets à la baseline, c'est-à-dire avant la prise de produit, sera réalisée par la statistique descriptive des paramètres démographiques disponibles (âge, sexe, taille, poids et IMC).

**Analyse principale**

Le critère principal de jugement sera la glycémie à jeun avant et après la complémentation. Elle sera comparée en intra-groupe et entre les deux groupes au moyen d’un test de comparaison des moyennes.

**Analyses secondaires**

Les autres comparaisons (critères secondaires de jugement) seront les suivantes :

- le taux d’HbA1c avant et après la complémentation. La comparaison sera réalisée en inter et intra-groupe

- les concentrations en cholestérol total, HDL, LDL, TG, leptine avant et après la complémentation. La comparaison sera effectuée inter- et intra-groupe

- les valeurs d’insulinémie et HOMA avant et après la complémentation. Comparaison inter et intra-groupe

- la qualité de vie avant et après complémentation

- le statut inflammatoire systémique (IL-6, CRPus)

- la taille adipocytaire avant et après complémentation

- le statut inflammatoire du tissu adipeux in vitro par dosage Elisa (IL-6, adiponectine)

- la sensibilité à l’insuline du tissu adipeux in vitro par étude de la sécrétion de l’adiponectine et de la phosphorylation d’Akt

# Cnil

Cette étude entre dans le cadre de la « Méthodologie de Référence » (MR-001) en application des dispositions de la loi du 6 août 2004 relative à la protection des personnes physiques à l’égard des traitements de données à caractère personnel et modifiant la loi du 6 janvier 1978 relative à l’informatique, aux fichiers et aux libertés. Ce changement a été homologué par décision du 5 janvier 2006.

# Archivage

Le laboratoire PiLeJe, en tant que promoteur, conserve les documents et données relatifs à l’essai conformément aux dispositions législatives et réglementaires et vigueur, notamment l’arrêté du 8 novembre 2006 fixant la durée de conservation par le promoteur et l’investigateur des documents et données relatifs à une recherche biomédicale portant sur un médicament à usage humain.

Cette durée de conservation des documents est fixée à 15 ans.

Les documents concernés sont notamment :

- la brochure pour l’investigateur ;
- le protocole et les amendements éventuels signés, spécimens du cahier d’observation et des carnets de bord et tout document/support servant au recueil des données ;
- information communiquée à la personne qui se prête à la recherche et formulaire de recueil de son consentement ;
- aspects financiers de la recherche ;
- contrat d’assurance garantissant la responsabilité civile du promoteur et attestation délivrée par l’assureur ;
- contrats ou conventions signés entre les différentes personnes (promoteur, organisme prestataire de services, investigateurs, etc…) ;
- dossier de demande d’avis auprès du CPP, échanges de correspondances et avis du CPP ;
- nom des personnes du CPP ayant délibéré sur le projet de la recherche ;
- *Curriculum vitae* des investigateurs et des collaborateurs impliqués dans la recherche, liste des collaborateurs de l’investigateur ;
- Cahiers d’observation complétés, datés et signés ;
- Les formulaires d’EI, EIG, EIGI.

Le laboratoire PiLeJe, en tant que promoteur, informe par écrit les investigateurs de la nécessité de conserver les documents et données relatifs à l’essai jusqu’à ce qu’ils leur notifient que cela ne sera plus nécessaire.

# Communication

Les données ne seront divulguées qu’après accord conjoint préalable de l’investigateur et du promoteur. Les résultats feront l’objet de communications et de publications.

De plus, à l’issue de la recherche, les personnes qui s’y prêtent ont le droit d'être informées des résultats globaux de cette recherche, selon les modalités qui leur seront précisées dans le document d'information (article L. 1121-1 du Code de la Santé publique).

# Modifications substantielles du protocole

Les modifications apportées au protocole devront être qualifiées de substantielles ou non.

Elles feront, selon leur nature, l’objet d’un nouvel avis du Comité de Protection des Personnes et de l’AFSSAPS.

Toute modification substantielle du protocole, concernant les objectifs de l'étude, son plan, la population, les examens ou des aspects administratifs significatifs, nécessitera l’approbation de l'investigateur, du promoteur, l’avis favorable du CPP. Un nouveau consentement des personnes participant à la recherche sera recueilli si nécessaire.

# Confidentialité

## Accès aux données

Le promoteur est chargé d’obtenir l’accord de l’ensemble des parties impliquées dans la recherche afin de garantir l’accès direct à tous les lieux de déroulement de la recherche, aux données source, aux documents source et aux rapports dans un but de contrôle de qualité et d’audit par le promoteur.

Les investigateurs mettront à disposition les documents et données individuelles strictement nécessaires au suivi, au contrôle de qualité et à l’audit de la recherche biomédicale, à la disposition des personnes ayant un accès à ces documents conformément aux dispositions législatives et réglementaires en vigueur (articles L.1121-3 et R.5121-13 du Code de la Santé Publique).

## Données source

Les documents source étant définis comme tout document ou objet original permettant de prouver l'existence ou l'exactitude d'une donnée ou d'un fait enregistrés au cours de l’étude clinique seront conservés pendant 15 ans par l'investigateur ou par l'hôpital s'il s'agit d'un dossier médical hospitalier. Dans cette étude seul le dossier médical est considéré comme donnée source.

## Confidentialité des données

Conformément aux dispositions concernant la confidentialité des données auxquelles ont accès les personnes chargées du contrôle de qualité d’une recherche biomédicale (article L.1121-3 du Code de la Santé Publique), conformément aux dispositions relatives à la confidentialité des informations concernant notamment la nature des médicaments expérimentaux, les essais, les personnes qui s'y prêtent et les résultats obtenus (article R. 5121-13 du Code de la Santé Publique), les personnes ayant un accès direct prendront toutes les précautions nécessaires en vue d'assurer la confidentialité des informations relatives aux médicaments expérimentaux, aux essais, aux personnes qui s'y prêtent et notamment en ce qui concerne leur identité ainsi qu’aux résultats obtenus.

Ces personnes, au même titre que les investigateurs eux-mêmes, sont soumises au secret professionnel (selon les conditions définies par les articles 226-13 et 226-14 du Code Pénal).

Pendant la recherche biomédicale ou à son issue, les données recueillies sur les personnes qui s’y prêtent et transmises au promoteur par les investigateurs (ou tous autres intervenants spécialisés) seront rendues anonymes.

Elles ne doivent en aucun cas faire apparaître en clair les noms des personnes concernées ni leur adresse.

Seules les trois premières lettres du nom du sujet et les deux premières lettres de son prénom seront enregistrées, accompagnées d’un numéro codé propre à l’étude indiquant l’ordre d’inclusion des sujets.

Le promoteur s’assurera que chaque personne qui se prête à la recherche a donné son accord par écrit pour l’accès aux données individuelles la concernant et strictement nécessaires au contrôle de qualité de la recherche.

## Inscription au fichier national des personnes se prêtant à une recherche biomédicale

En application de l’article L. 1121-16 du code de la santé publique, les personnes qui se prêtent à la recherche biomédicale seront inscrites au fichier national des personnes qui se prêtent à des recherches biomédicales : VRB (Volontaires Pour la Recherche biomédicale)

Les investigateurs seront les seules personnes habilitées à inscrire les volontaires dans ce fichier.

# Assurance qualité

## Engagement des investigateurs et du promoteur

L'investigateur s'engage à ce que cette étude soit réalisée en conformité avec la loi de santé publique n°2004-806 du 9 août 2004 concernant les recherches biomédicales, le décret d’application n° 2006-477 du 26/04/2006 modifiant le chapitre I^er^ du titre II du livre 1^er^ de la première partie du Code de la Santé Publique relatif aux recherches biomédicales ainsi que les arrêtés en vigueur.

Les règles de bonnes pratiques cliniques (BPC) pour les recherches biomédicales portant sur des médicaments à usage humain, mentionnées à l’article L. 1121-3 du Code de la Santé Publique et à l’arrêté du 24 novembre 2006 seront également appliquées (cf. Annexe 10).

L’investigateur s’engage également à travailler en accord avec la Déclaration d'Helsinki de l’Association Médicale Mondiale (Tokyo 2004, révisée). (Cf. Annexe 9).

## Assurance de Qualité

Un Attaché de Recherche Clinique (ARC) mandaté par le promoteur s’assurera de la bonne réalisation de l’étude, du recueil des données générés par écrit, de leur documentation, enregistrement et rapport, en accord avec les Procédures et conformément aux Bonnes Pratiques Cliniques ainsi qu’aux dispositions législatives et réglementaires en vigueur.

## Contrôle de la Qualité

L’investigateur se porte garant de l’authenticité des données recueillies dans le cadre de l’étude et accepte les dispositions légales autorisant le promoteur de l’étude à mettre en place un contrôle de qualité.

L'investigateur et les investigateurs associés acceptent donc de se rendre disponibles lors des visites de Contrôle de Qualité effectuées à intervalles réguliers par l’Attaché de Recherche Clinique. Lors de ces visites, les éléments suivants seront revus :

- consentement éclairé et formulaire d’information ;
- respect du protocole de l'étude et des procédures qui y sont définies ;
- qualité des données recueillies dans le cahier d'observation : exactitude, données manquantes, cohérence des données avec les documents "source" (dossiers médicaux, carnets de rendez-vous, originaux des résultats de laboratoire, etc.…) ;
- gestion des produits éventuels.

## Cahier d’observation

Toutes les informations requises par le protocole doivent être consignées sur les cahiers d’observation et une explication doit être apportée pour chaque donnée manquante. Les données devront être recueillies au fur et à mesure qu'elles sont obtenues, et transcrites dans ces cahiers de façon nette et lisible.

Les données erronées relevées sur les cahiers d'observation seront clairement barrées et les nouvelles données seront copiées, à côté de l'information barrée, accompagnées des initiales, de la date et éventuellement d’une justification par l’investigateur ou la personne autorisée qui aura fait la correction.

# Références bibliographiques

Reference List

1. Report of the Expert Committee on the Diagnosis and Classification of Diabetes Mellitus. Diabetes Care 1997;20:1183-97.

2. King H, Rewers M. Global estimates for prevalence of diabetes mellitus and impaired glucose tolerance in adults. WHO Ad Hoc Diabetes Reporting Group. Diabetes Care 1993;16:157-77.

3. Wild S, Roglic G, Green A, Sicree R, King H. Global prevalence of diabetes: estimates for the year 2000 and projections for 2030. Diabetes Care 2004;27:1047-53.

4. Drewnowski A, Specter SE. Poverty and obesity: the role of energy density and energy costs. Am J Clin Nutr 2004;79:6-16.

5. Charles MA, Eschwege E, Basdevant A. Monitoring the obesity epidemic in France: the Obepi surveys 1997-2006. Obesity (Silver Spring) 2008;16:2182-6.

6. Report of the Expert Committee on the Diagnosis and Classification of Diabetes Mellitus. Diabetes Care 1997;20:1183-97.

6 bis. Alberti GK, Eckel RH, Grundy SM et coll. Harmonizing the Metabolic Syndrome. A Joint Interim Statement of the International Diabetes Federation Task Force on Epidemiology and Prevention; National Heart Lung and Blood Institute; American Heart Association; World Heart Federation; International Atherosclerosis Society; and International Association for the Study of Obesity. *Circulation* 2009;120:1640-45.

7. The Diabetes Prevention Program (DPP): description of lifestyle intervention. Diabetes Care 2002;25:2165-71.

8. Pan XR, Li GW, Hu YH et al. Effects of diet and exercise in preventing NIDDM in people with impaired glucose tolerance. The Da Qing IGT and Diabetes Study. Diabetes Care 1997;20:537-44.

9. Chiasson JL. Acarbose for the prevention of diabetes, hypertension, and cardiovascular disease in subjects with impaired glucose tolerance: the Study to Prevent Non-Insulin-Dependent Diabetes Mellitus (STOP-NIDDM) Trial. Endocr Pract 2006;12 Suppl 1:25-30.

10. Hoerger TJ, Hicks KA, Sorensen SW et al. Cost-effectiveness of screening for pre-diabetes among overweight and obese U.S. adults. Diabetes Care 2007;30:2874-9.

11. Jacob S, Rabbia M, Meier MK, Hauptman J. Orlistat 120 mg improves glycaemic control in type 2 diabetic patients with or without concurrent weight loss. Diabetes Obes Metab 2009;11:361-71.

12. Anderson RA, Broadhurst CL, Polansky MM et al. Isolation and characterization of polyphenol type-A polymers from cinnamon with insulin-like biological activity. J Agric Food Chem 2004;52:65-70.

13. Broadhurst CL, Polansky MM, Anderson RA. Insulin-like biological activity of culinary and medicinal plant aqueous extracts in vitro. J Agric Food Chem 2000;48:849-52.

14. Jarvill-Taylor KJ, Anderson RA, Graves DJ. A hydroxychalcone derived from cinnamon functions as a mimetic for insulin in 3T3-L1 adipocytes. J Am Coll Nutr 2001;20:327-36.

15. Qin B, Nagasaki M, Ren M, Bajotto G, Oshida Y, Sato Y. Cinnamon extract (traditional herb) potentiates in vivo insulin-regulated glucose utilization via enhancing insulin signaling in rats. Diabetes Res Clin Pract 2003;62:139-48.

16. Qin B, Nagasaki M, Ren M, Bajotto G, Oshida Y, Sato Y. Cinnamon extract prevents the insulin resistance induced by a high-fructose diet. Horm Metab Res 2004;36:119-25.

17. Sheng X, Zhang Y, Gong Z, Huang C, Zang YQ. Improved Insulin Resistance and Lipid Metabolism by Cinnamon Extract through Activation of Peroxisome Proliferator-Activated Receptors. PPAR Res 2008;2008:581348.

18. Qin B, Nagasaki M, Ren M, Bajotto G, Oshida Y, Sato Y. Cinnamon extract prevents the insulin resistance induced by a high-fructose diet. Horm Metab Res 2004;36:119-25.

19. Altschuler JA, Casella SJ, MacKenzie TA, Curtis KM. The effect of cinnamon on A1C among adolescents with type 1 diabetes. Diabetes Care 2007;30:813-6.

20. Baker WL, Gutierrez-Williams G, White CM, Kluger J, Coleman CI. Effect of cinnamon on glucose control and lipid parameters. Diabetes Care 2008;31:41-3.

21. Blevins SM, Leyva MJ, Brown J, Wright J, Scofield RH, Aston CE. Effect of cinnamon on glucose and lipid levels in non insulin-dependent type 2 diabetes. Diabetes Care 2007;30:2236-7.

22. Khan A, Safdar M, Ali Khan MM, Khattak KN, Anderson RA. Cinnamon improves glucose and lipids of people with type 2 diabetes. Diabetes Care 2003;26:3215-8.

23. Mang B, Wolters M, Schmitt B et al. Effects of a cinnamon extract on plasma glucose, HbA, and serum lipids in diabetes mellitus type 2. Eur J Clin Invest 2006;36:340-4.

24. Vanschoonbeek K, Thomassen BJ, Senden JM, Wodzig WK, van Loon LJ. Cinnamon supplementation does not improve glycemic control in postmenopausal type 2 diabetes patients. J Nutr 2006;136:977-80.

25. Ziegenfuss TN, Hofheins JE, Mendel RW, Landis J, Anderson RA. Effects of a water-soluble cinnamon extract on body composition and features of the metabolic syndrome in pre-diabetic men and women. J Int Soc Sports Nutr 2006;3:45-53.

26. Hlebowicz J, Hlebowicz A, Lindstedt S et al. Effects of 1 and 3 g cinnamon on gastric emptying, satiety, and postprandial blood glucose, insulin, glucose-dependent insulinotropic polypeptide, glucagon-like peptide 1, and ghrelin concentrations in healthy subjects. Am J Clin Nutr 2009;89:815-21.

27. Solomon TP, Blannin AK. Changes in glucose tolerance and insulin sensitivity following 2 weeks of daily cinnamon ingestion in healthy humans. Eur J Appl Physiol 2009;105:969-76.

28. Solomon TP, Blannin AK. Changes in glucose tolerance and insulin sensitivity following 2 weeks of daily cinnamon ingestion in healthy humans. Eur J Appl Physiol 2009;105:969-76.

29. Hlebowicz J, Hlebowicz A, Lindstedt S et al. Effects of 1 and 3 g cinnamon on gastric emptying, satiety, and postprandial blood glucose, insulin, glucose-dependent insulinotropic polypeptide, glucagon-like peptide 1, and ghrelin concentrations in healthy subjects. Am J Clin Nutr 2009;89:815-21.

30. Khan A, Safdar M, Ali Khan MM, Khattak KN, Anderson RA. Cinnamon improves glucose and lipids of people with type 2 diabetes. Diabetes Care 2003;26:3215-8.

31. Mang B, Wolters M, Schmitt B et al. Effects of a cinnamon extract on plasma glucose, HbA, and serum lipids in diabetes mellitus type 2. Eur J Clin Invest 2006;36:340-4.

32. Vanschoonbeek K, Thomassen BJ, Senden JM, Wodzig WK, van Loon LJ. Cinnamon supplementation does not improve glycemic control in postmenopausal type 2 diabetes patients. J Nutr 2006;136:977-80.

33. Altschuler JA, Casella SJ, MacKenzie TA, Curtis KM. The effect of cinnamon on A1C among adolescents with type 1 diabetes. Diabetes Care 2007;30:813-6.

34. Blevins SM, Leyva MJ, Brown J, Wright J, Scofield RH, Aston CE. Effect of cinnamon on glucose and lipid levels in non insulin-dependent type 2 diabetes. Diabetes Care 2007;30:2236-7.

35. Baker WL, Gutierrez-William G, White CM, Kluger J, Coleman CI. Effect fo Cinnamon on Glucose Control and Lipid Parameters. Diabetes Care 8 A.D.;31:41-3.

36. Ziegenfuss TN, Hofheins JE, Mendel RW, Landis J, Anderson RA. Effects of a water-soluble cinnamon extract on body composition and features of the metabolic syndrome in pre-diabetic men and women. J Int Soc Sports Nutr 2006;3:45-53.

37. Morris BW, Macneil S, Hardisty CA, Heller S, Burgin C, Gray TA. Chromium homeostasis in patients with type II (NIDDM) diabetes. Journal of Trace Elements in Medicine and Biology 1999;13:57-61.

38. Pineau A, Guillard O, Risse JF. A study of chromium in human cataractous lenses and whole blood of diabetics, senile and normal population. Biological Trace Element Research 1992;32:133-8.

39. Anderson RA, Polansky MM, Bryden NA, Bhatena SJ, Canary JJ. Effects of supplemental chromium on patients with symptoms of reactive hypoglycemia. Metabolism 1987;36:351-5.

40. Anderson RA. Chromium, glucose intolerance and diabetes. Journal of the American College of Nutrition 1998;17:548-55.

41. Mertz W. Chromium in human nutrition: a review. Journal of Nutrition 1993;123:626-33.

42. Anderson RA, Cheng N, Bryden NA et al. Elevated intakes of supplemental chromium improve glucose and insulin variables in individuals with type 2 diabetes. Diabetes 1997;46:1786-91.

43. Balk EM, Tatsioni A, Lichtenstein AH, Lau J, Pittas AG. Effect of chromium supplementation on glucose metabolism and lipids: a systematic review of randomized controlled trials. Diabetes Care 2007;30:2154-63.

44. Zima T, Mestek O, Tesar V, Tesarova P, Zak A, Zeman M. Chromium levels in patients with internal diseases. Biochemistry and Molecular Biology International 1998;46:365-74.

45. Urberg M, Remel MB. Evidence for synergism between chromium and nicotinic acid in the control of glucose tolerance in elderly humans. Metabolism 1987;36:896-9.

46. Reddy VP, Garrett MR, Perry G, Smith MA. Carnosine: a versatile antioxidant and antiglycating agent. Sci Aging Knowledge Environ 2005;2005:e12.

47. Szwergold BS. Carnosine and anserine act as effective transglycating agents in decomposition of aldose-derived Schiff bases. Biochem Biophys Res Commun 2005;336:36-41.

48. Ferraris RP, Diamond J, Kwan WW. Dietary regulation of intestinal transport of the dipeptide carnosine. Am J Physiol 1988;255:G143-G150.

49. Chan WK, Decker EA, Chow CK, Boissonneault GA. Effect of dietary carnosine on plasma and tissue antioxidant concentrations and on lipid oxidation in rat skeletal muscle. Lipids 1994;29:461-6.

50. Lee YT, Hsu CC, Lin MH, Liu KS, Yin MC. Histidine and carnosine delay diabetic deterioration in mice and protect human low density lipoprotein against oxidation and glycation. Eur J Pharmacol 2005;513:145-50.

# Annexes

## Annexe 1 : Lettre d’information version 1.2

## Annexe 2 : Consentement éclairé version 1.3

## Annexe 3 : Copie de l’attestation d’assurance

## Annexe 4 : Autorisation du lieu de recherche

## Annexe 5 : Copies de l’avis favorable du CPP datant du 20/12/2010 et de l’Afssaps datant du 2/11/2010

## Annexe 6 : Questionnaires TFEQ et d’activité physique

## Annexe 7 : Carnet alimentaire

## Annexe 8 : Annonce « Métro »

## Annexe 9 : Déclaration d’Helsinki

## Annexe 10 : BPC
