# Supplementary material for: A Dietary Supplement Containing Cinnamon, Chromium and Carnosine Decreases Fasting Plasma Glucose and Increases Lean Mass in Overweight or Obese Pre-Diabetic Subjects: A Randomized, Placebo-Controlled Trial
Source: PLoS One. 2015 Sep 25;10(9):e0138646. doi: 10.1371/journal.pone.0138646 (PMC4583280; doi:10.1371/journal.pone.0138646)
Supplement: S1 File — (DOC) [file pone.0138646.s004.doc]

**Online supporting information S1**

**Table A. Transformations used for each variable**

NA: not applicable; -: no transformation; Log: logarithm transformation; Ansc: Anscombe transformation; BMI: body mass index; HbA1c: glycated hemoglobin; HOMA-IR: homeostatic model assessment-insulin resistance; HOMA-B (%): β cell function; HOMA-S (%): insulin sensitivity; QUICKI: quantitative insulin sensitivity check index; HDL: high-density lipoprotein; LDL: low-density lipoprotein; FFA: free fatty acids; hs-CRP: high-sensitivity C-reactive protein; PAI-1: plasminogen activator inhibitor-1; IL-6: interleukin-6; Akt: serine/threonine protein kinase B.

**Table B. Baseline characteristics of complier subjects and
subjects not considered in the per-protocol efficacy analysis**

Values are mean±SD. BMI: body mass index. Compliers included in the per-protocol efficacy analysis and subjects not considered in this analysis were compared using Student’s t-tests for quantitative variables and Fisher's exact test for sex. No statistically significant difference was found between these two subject categories.

**Table C. Physical activity scores at baseline and after 4 Month treatment in both the dietary supplement and placebo groups**

|  | **Placebo (n=26)** | | | **Dietary supplement (n=26)** | | | **Change in**  **placebo *vs*. dietary supplement**  ***P* value** |
| --- | --- | --- | --- | --- | --- | --- | --- |
|  | **Baseline**  (Day 0) | **After treatment** (M4) | **P value**  Day 0  *vs*. M4 | **Baseline**  (Day 0) | **After treatment** (M4) | **P value**  Day 0 *vs*. M4 |
| **Dietary intake** | | | | | | | |
| WI | 1.7 ± 1.3 | 1.9 ± 1.2 | 0.58 | 1.6 ± 1.3 | 1.5 ± 1.3 | 0.45 | 0.63 |
| SI | 3.1 ± 1.0 | 2.8 ± 1.0 | 0.11 | 2.8 ± 0.8 | 2.6 ± 1.0 | 0.07 | 0.78 |
| LI | 2.8 ± 0.8 | 2.8 ± 0.8 | 0.85 | 2.5 ± 0.7 | 2.5 ± 0.6 | 0.64 | 0.51 |

Values are expressed as mean±SD. M4: Month 4; WI: walking index based on physical activity questionnaire; SI: sport index based on physical activity questionnaire; LI: leisure index based on physical activity questionnaire. Baseline data did not differ between groups using unpaired Student t-tests. Baseline and 4-month data in each group were compared using paired Student’s t-tests; Changes (100*[values at 4 months – values at baseline]/values at baseline) in the placebo and dietary supplement groups were compared using unpaired Student’s t-tests. Habitual physical activity was evaluated by the Baecke questionnaire.

*Baecke JAH Burema J Frijters ER. A short questionnaire for the measurement of habitual physical activity in epidemiological studies. Am J Clin Nutr. 1982; 36: 936-942*.

**Table D. Efficacy analysis in the intention-to-treat population**

Values are mean±SD. M4: Month 4; BMI: body mass index; HbA1c: glycated hemoglobin; HOMA-IR: homeostatic model assessment-insulin resistance; HOMA-B (%): β cell function; HOMA-S (%): insulin sensitivity; QUICKI: quantitative insulin sensitivity check index; HDL: high-density lipoprotein; LDL: low-density lipoprotein; FFA: free fatty acids; hs-CRP: high-sensitivity C-reactive protein; PAI-1: plasminogen activator inhibitor-1; IL-6: interleukin-6; Akt: serine/threonine protein kinase B. Baseline and 4-month data in each group were compared using paired Student’s t-tests; Changes (values at 4 months – values at baseline) in the placebo and dietary supplement groups were compared using unpaired Student’s t-tests.

**Table E**. Adverse events

| **No. of patients with at least one event (%)** | **Placebo (n=32)** | **Dietary supplement**  **(n=30)** |
| --- | --- | --- |
| Adverse event | 24 (75.0) | 27 (90.0) |
| Severe | 2 (6.3) | 2 (6.7) |
| Related to study treatment | 4 (12.5) | 4 (13.3) |
| Serious adverse event | 4 (12.5) | 5 (16.7) |
| Related to study treatment | 0 (0.0) | 0 (0.0) |

**Table F. Comparisons of baseline characteristics of good *vs*. poor responders
to the dietary supplement***

*The definitions of good and poor responders are stated in the body text and Figure 2A of the printed manuscript. Values are mean ± SD. Paired Mann-Whitney t-tests were used for comparisons. BMI: body mass index; HbA1c: glycated hemoglobin; HOMA-IR: homeostatic model assessment-insulin resistance; HOMA-B (%): β cell function; HOMA-S (%): insulin sensitivity; QUICKI: quantitative insulin sensitivity check index; HDL: high-density lipoprotein; LDL: low-density lipoprotein; FFA: free fatty acids; hs-CRP: high-sensitivity C-reactive protein; PAI-1: plasminogen activator inhibitor-1; IL-6: interleukin-6; Akt: serine/threonine protein kinase B.

**Figure A. FPG (mmol/L) over time**

Data are mean±sem. FPG (fasting plasma glucose) at Month 0 (beginning of treatment), at Month 4 (end of treatment period) and at Month 6 (end of follow-up period). A linear mixed model showed a p-value of 0.05 for the interaction between time and treatment effects. We compared variations among treatments between baseline and M4 (**p=0.022**), and between M4 and M6 (p=0.063).

**Figure B. Profiles of changes in FPG (%) in the dietary supplement and placebo groups**

Data are mean±sem.

Study duration (months)

B

a

b

0

4

6

Study duration (months)

B

a

b

0

4

6

In an attempt to understand the effect of the dietary supplement on fasting plasma glucose (FPG), and to monitor FPG responses during treatment and follow-up, subjects in both the dietary supplement group and placebo group were clustered according to their FPG profiles. The use of K-Means for Longitudinal Data (KmL) [1] was thought to be adequate because of its ability to take into account individual trajectories in longitudinal data**. Figure A:** in the dietary supplement group, three different clusters were identified**:** Cluster a (n=6) showed a decline in FPG during treatment (-5%) and then again till -11% at the end of follow-up; in Cluster b (n=11), FPG decreased during treatment (-9.7%) and then returned to baseline (-0.2%); Cluster c (n=9) manifested a slight elevation of FPG during treatment (4.7%) that remained constant during follow-up. Figure B: in the placebo group only two clusters were identified: cluster “a” (n=17 subjects) declined slightly (-2.7%) during the treatment period and remained stable throughout follow-up. The second cluster (cluster b) consisted of nine subjects and showed an elevation of 11% during the treatment period then a reduction at the end of follow-up. Clusters in the placebo group were totally different from those in the treatment group, : while two clusters in the dietary supplement group (representing 65% of included subjects) showed a decrease of either -5 or -10 of FPG, in the placebo group only one cluster showed a slight decrease of -2.7% in 65% of the subjects.

In the dietary supplement group (Figure A), in an attempt to find if special clinical characteristics at baseline may predict the different trajectories of FPG, the baseline characteristics of the 3 clusters were compared. Sex, age, and adiposity markers were similar, as were physical activity and psychological scores. Of importance, total calorie intake, nutrients, food items and clinical parameters were similar in the 3 clusters.

*Genolini C, Falissard B (2011) KmL: a package to cluster longitudinal data. Comput Methods Programs Biomed 104:e112-21.*

**Figure C. Relationship between change in FPG (%) and baseline FPG (mmol/L)
in the dietary supplement and placebo groups**

**A**

**B**

Relationship between change in FPG (fasting plasma glucose) and baseline FPG during 4 months of dietary supplementation (A, n=26) and placebo (B, n=26) (Pearson correlation).

**Figure D. Relationship between change in FPG (%) and baseline
plasma IL-6 level (pg/mL) in the dietary supplement and placebo groups**

**A**

**B**

Relationship between change in FPG (fasting plasma glucose) during 4 months and baseline plasma IL-6 level; in the dietary supplement (n=26) and placebo group (n=26) (Pearson correlation).
